# Piwil2 (Mili) sustains neurogenesis and prevents cellular senescence in the postnatal hippocampus

**DOI:** 10.15252/embr.202153801

**Published:** 2022-12-06

**Authors:** Caterina Gasperini, Kiril Tuntevski, Silvia Beatini, Roberta Pelizzoli, Amanda Lo Van, Damiano Mangoni, Rosa M Cossu, Giovanni Pascarella, Paolo Bianchini, Pascal Bielefeld, Margherita Scarpato, Meritxell Pons‐Espinal, Remo Sanges, Alberto Diaspro, Carlos P Fitzsimons, Piero Carninci, Stefano Gustincich, Davide De Pietri Tonelli

**Affiliations:** ^1^ Neurobiology of miRNA Laboratory Istituto Italiano di Tecnologia Genoa Italy; ^2^ The Open University Affiliated Research Centre at Istituto Italiano di Tecnologia (ARC@IIT) Genoa Italy; ^3^ Central RNA Laboratory Istituto Italiano di Tecnologia Genoa Italy; ^4^ Division of Genomic Technologies RIKEN Center for Life Science Technologies Yokohama Japan; ^5^ Nanoscopy, CHT Erzelli Istituto Italiano di Tecnologia Genoa Italy; ^6^ Swammerdam Institute for Life Sciences, Faculty of Science University of Amsterdam Amsterdam The Netherlands; ^7^ Area of Neuroscience SISSA Trieste Italy; ^8^ Human Technopole Milan Italy

**Keywords:** hippocampal neurogenesis, Mili, Piwi‐interacting RNAs, Piwil2, senescence, Neuroscience, RNA Biology

## Abstract

Adult neural progenitor cells (aNPCs) ensure lifelong neurogenesis in the mammalian hippocampus. Proper regulation of aNPC fate has thus important implications for brain plasticity and healthy aging. Piwi proteins and the small noncoding RNAs interacting with them (piRNAs) have been proposed to control memory and anxiety, but the mechanism remains elusive. Here, we show that Piwil2 (Mili) is essential for proper neurogenesis in the postnatal mouse hippocampus. RNA sequencing of aNPCs and their differentiated progeny reveal that Mili and piRNAs are dynamically expressed in neurogenesis. Depletion of Mili and piRNAs in the adult hippocampus impairs aNPC differentiation toward a neural fate, induces senescence, and generates reactive glia. Transcripts modulated upon Mili depletion bear sequences complementary or homologous to piRNAs and include repetitive elements and mRNAs encoding essential proteins for proper neurogenesis. Our results provide evidence of a critical role for Mili in maintaining fitness and proper fate of aNPCs, underpinning a possible involvement of the piRNA pathway in brain plasticity and successful aging.

## Graphical abstract

Functions of the Piwi proteins and Piwi‐interacting RNAs have been related to memory and neurodegeneration, but the mechanisms remain elusive. This study reports that Piwil2 safeguards aNPC fitness and proper neurogenesis in the postnatal mouse hippocampus.

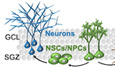

## Introduction

A regulated balance of aNPC quiescence, proliferation, and differentiation guarantees lifelong neurogenesis in the adult hippocampus (Altman, 1962; Doetsch *et al*, 1999), prevents the generation of reactive glia (Encinas *et al*, 2011; Sierra *et al*, 2015; Clarke *et al*, 2018), and curbs neurodegeneration (Toda *et al*, 2019). Understanding the molecular control of aNPCs fate is pivotal to develop novel therapies aimed at preventing or delay age‐dependent loss of neurogenesis and related pathological conditions.

The *Piwi* genes encode for an evolutionary conserved subfamily of Argonaute proteins that bind to Piwi‐interacting RNAs (piRNAs), a class of single‐stranded noncoding RNAs of 21–35 nucleotides. Piwi proteins and piRNAs (henceforth referred to as the piRNA pathway) are highly abundant in gonads, where they mainly target transposable elements (TEs) for degradation to maintain germline stem cell pools and male fertility (Czech *et al*, 2018; Ozata *et al*, 2019). Since its initial discovery, the piRNA pathway has been also implicated in regulating gene expression outside gonads, particularly in somatic stem cells (Rojas‐Rıós & Simonelig, 2018). In fact, Piwi proteins are present in human hematopoietic stem cells, but their functions are dispensable for normal hematopoiesis in the mouse (Nolde *et al*, 2013), suggesting a possible role of the piRNA pathway in the control of self‐renewal, rather than differentiation of these cells (Sharma *et al*, 2001).

Besides gonads, the highest piRNA expression in the adult mouse has been observed in the hippocampus (Perera *et al*, 2019), and proposed to control synaptic plasticity, memory, and anxiety (Lee *et al*, 2011; Zhao *et al*, 2015; Nandi *et al*, 2016; Leighton *et al*, 2019). piRNA abundance in neurons, however, is low compared to that of germline cells (Lee *et al*, 2011; Ghosheh *et al*, 2016; Nandi *et al*, 2016). Moreover, TE expression increases following the differentiation of NPC (Muotri *et al*, 2005), in parallel with the number of somatic TE insertions found in hippocampal neurons (Upton *et al*, 2015), arguing against functions of the piRNA pathway in postmitotic nerve cells. Given that the hippocampus is one of the niches in which neurogenesis persists beyond embryonic age, we hypothesize that the piRNA pathway may be present in aNPCs, possibly contributing to maintain the neurogenesis capacity lifelong (Penning *et al*, 2022).

Here, we studied the piRNA pathway in aNPCs of the postnatal mouse hippocampus *in vivo* and *in vitro*. By knockdown (KD) of Mili (i.e., one of the essential endoribonucleases for piRNA biogenesis and function) and Mili‐dependent piRNAs, we investigated the consequences of their depletion for proliferation, survival, differentiation, and fate of aNPCs. With this approach, we aim to address functions of the piRNA pathway in adult neurogenesis, hence providing a system‐level biological understanding of scientific and therapeutic value for brain plasticity and successful aging.

## Results

### Mili is preferentially expressed in aNPCs and depleted in neurogenesis

**Figure 1:**
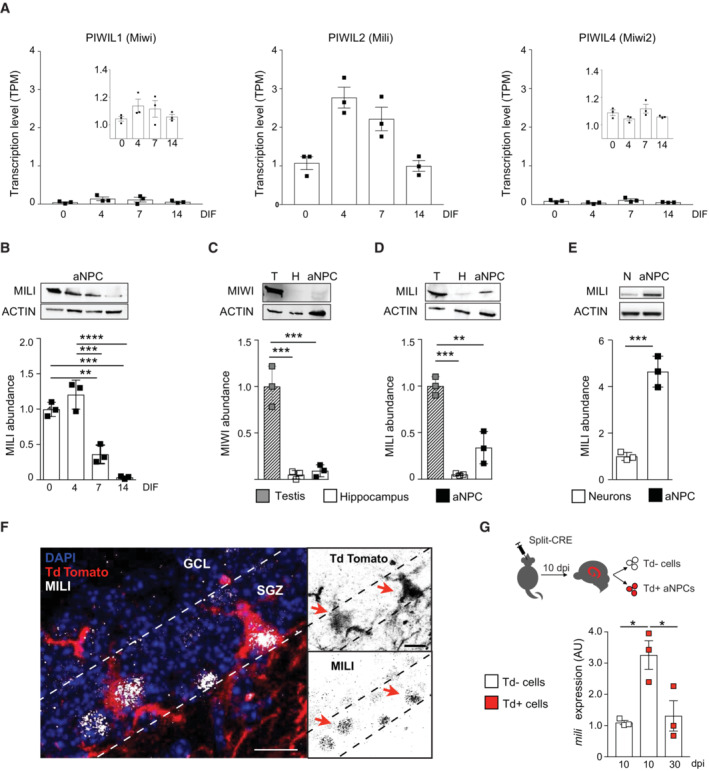
Mili is preferentially expressed in aNPCs and depleted in neurogenesis ALevels of *Piwil1 (Miwi)*, *Piwil2 (Mili)*, and *Piwil4 (Miwi2)* transcripts in RNA seq. data from undifferentiated aNPCs (Days of differentiation—DIF—0) and differentiating neuroblasts (DIF4‐14); insets in left and right panels show the same data with smaller scales in the ordinate axes.BWestern blot (inset) and quantification (bar graph) of Mili protein abundance in DIF0 aNPCs and differentiating neuroblasts upon viral‐induced neurogenesis (DIF4‐14).C, DWestern blot (insets) and quantification (bar graphs) of Miwi and Mili protein abundance in lysates from postnatal mouse testis, hippocampus, and undifferentiated aNPCs cultures.EWestern blot (inset) and quantification (bar graph) of Mili protein abundance in lysates from cultured mouse hippocampal neurons and undifferentiated aNPCs.FRepresentative immunofluorescence micrograph of Mili (white) and Td^+^ NSCs (red) in hippocampal subgranular zone (SGZ); arrows indicate Td^+^ Mili^+^ double‐positive cells.GScheme of the experiment (top) and *Mili* mRNA expression in sorted Td^+^ and Td^−^ cells after *in vivo* transduction with split‐Cre viruses in the hippocampus (bottom). Data information: data are expressed as mean ± SEM, *n* = 3 biological replicates. *P < 0.05, **P < 0.01, ***P < 0.001, ****P < 0.0001 as assessed by one‐way ANOVA with the Bonferroni test (in B–D, G) and the two‐tailed Student's *t*‐test (in E). In (F): GCL, granular cell layer; H, Hilus. The scale bars represent 10 μm. Source data are available online for this figure.

As an entry point to investigate the piRNA pathway in aNPCs we quantified the expression of *Piwil1* (Miwi) *Piwil2* (Mili) and *Piwil4* (Miwi2) transcripts, encoding the three main Piwi proteins present in the adult mouse (Czech *et al*, 2018; Ozata *et al*, 2019). Analysis of deep RNA sequencing (RNA seq) of total RNA from cultured aNPCs derived from neural stem cells (NSC) of the adult mouse Dentate Gyrus (DG) (Walker & Kempermann, 2014; Pons‐Espinal *et al*, 2017) indicated that *Mili* is the most abundantly expressed of the three *Piwi* genes in neurogenesis (Fig 1A). In addition, other genes encoding for piRNA biogenesis cofactors were expressed in neurogenesis, including the transcription factors Zic2, Mybl1, and Meis1, the Tudor and KH domain‐containing protein Tdrkh, and the helicase Mov10 (Dataset EV1). Interestingly, *Mili* expression showed a dynamic trend in neurogenesis. Indeed, its expression increased transiently from proliferating aNPCs (here referred to as days of differentiation (DIF0)) showing a peak at DIF4 upon onset of their spontaneous differentiation, whereas it decreased in differentiated progeny (DIF7‐14) (Fig 1A). Similarly, the Mili protein abundance was higher in undifferentiated aNPCs (DIF0), or early upon onset of vector‐induced neurogenesis (DIF4), compared with neuroblasts and neurons (DIF7 and 14, respectively) (Fig 1B). Next, we quantified the abundance of Miwi and Mili proteins in the mouse testis, whole hippocampus, and undifferentiated aNPCs (Fig 1C and D). As expected, the Miwi protein was very abundant in the testis but almost undetectable in the whole hippocampus or aNPCs (Fig 1C), whereas the Mili protein abundance in aNPCs was about 40% of the one in the testes (Fig 1D), and about four‐fold higher than in primary hippocampal neurons (Fig 1E).

To validate this finding *in vivo*, we used the split‐Cre viral approach to selectively label NSCs and their progeny in the postnatal hippocampus of Td‐Tomato Cre‐reporter mice (Pons‐Espinal *et al*, 2017). Five days postviral injection (dpi), we found Mili protein in Td‐Tomato positive (Td^+^) NSCs of the subgranular zone (SGZ) of the DG (Fig 1F). To corroborate the immunofluorescence result, and to follow *Mili* expression during neurogenesis *in vivo*, we sorted Td^+^ NSCs and their differentiated progeny at 10 and 30 dpi in the postnatal mouse hippocampus, respectively, and quantified *Mili* by real‐time quantitative PCR (qPCR). The *Mili* transcript was significantly more abundant in Td^+^ NSCs (10 dpi) than in adult‐born Td^+^ neurons (30 dpi) or Td^−^ cells (Fig 1G). These results indicate that Mili expression is dynamic in neurogenesis, being enriched in neural stem/progenitor cells and depleted in their differentiated progeny.

### Identification and validation of piRNAs in aNPCs

**Figure 2:**
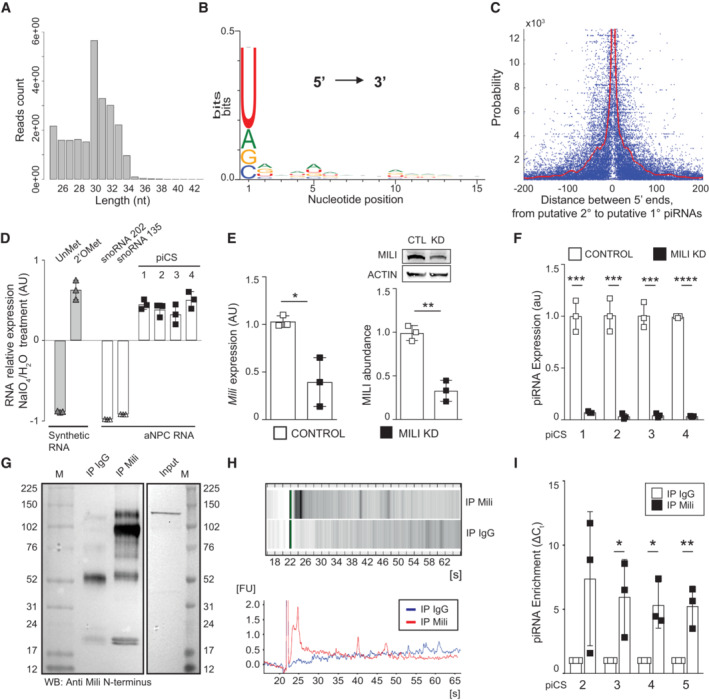
Identification and validation of piRNAs in aNPCs A–C(A) Size distribution of the piRNA reads showing (B) uridine bias at piRNA 5′ ends and (C) probability of distances from the 5′ ends of putative secondary piRNAs to the 5′ ends of putative primary piRNAs. Note in panel C that 5′ termini of putative primary and secondary piRNAs derived from the same cluster tend to concatenate around the “0” mark, as reported in other animals. Distance probability was assayed for unique piRNAs (length between 15–35 nucleotides), without taking into account abundance, by locally weighted smoothing linear regression (LOWESS).DRelative expression of transcripts bearing piRNA‐cluster consensus sequences (piCS) of the top abundant piRNAs, and two control snoRNAs (202 and 135) in aNPC, upon treatment with sodium periodate (NaIO_4_) or water and alkaline ß‐elimination. Synthetic RNA oligos were used as negative (Unmethylated, UnMet) and positive (2′‐O‐methylated, 2′OMet) controls, respectively. Note that the presence of 3′‐end 2′‐O‐methylation in positive control and piRNAs confers them resistance to periodate oxidation and alkaline ß‐elimination, in contrast to the depletion in UnMet negative control and snoRNAs.E
*Mili* mRNA expression (left bar graph); western blot (inset) and quantification (right bar graph) of Mili protein abundance in aNPCs upon viral transduction of scrambled shRNA (Control) or shRNA targeting *Mili* (Mili KD).FExpression of four of the most abundant piCS in control and Mili KD aNPCs.G–I Western blot (G), analysis by capillary electrophoresis (H), or quantification by qPCR (I) of the endogenous piCS after co‐immunoprecipitation (IP) with endogenous Mili (IP Mili), or control IgG (IP IgG) in lysates of DIF4 aNPCs. In the qPCR abundance of the indicated piCS in the IP Mili was normalized to its respective level in the control co‐immunoprecipitation (IgG); error bars in (I) represent standard deviation. Data information: data are expressed as mean ± SEM unless differently indicated, *n* = 2 biological replicates (A–C) and *n* = 3 biological replicates (D–F, I). *P < 0.05, **P < 0.01, ***P < 0.001, ****P < 0.0001, as assessed by the two‐tailed Student's *t*‐test. Source data are available online for this figure.

**Figure EV1:**
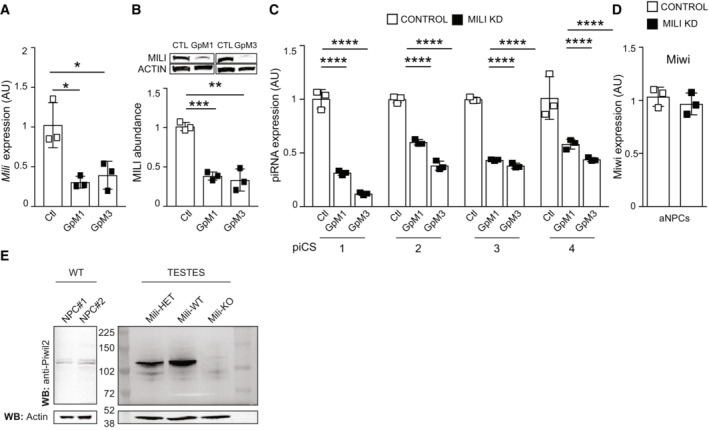
Mili KD depletes piRNAs and does not affect Miwi. Relative to Fig 2 A, B
*Mili* mRNA expression (A); western blot (B, inset) and quantification of Mili protein abundance (B, bar graph) in lysates from aNPCs upon transfection with control GapmeR (Ctl) or two different GapmeRs (GpM1, GpM3) targeting *Mili*.CExpression of transcripts of piRNA‐cluster consensus sequences (piCS) in control and Mili KD (two independent GapmeRs) aNPCs.DRelative expression of *Miwi* transcript in undifferentiated aNPCs transduced with viruses transcribing a Scrambled short hairpin (Control) and short hairpin against *Mili* (Mili KD).EWestern blot of Mili protein in lysates from aNPCs (*n* = 2 biological replicates) and from testes of mice wildtype (WT), heterozygous (HET) or knockout (KO) for the *Mili* gene. Data information: data are expressed as mean ± SEM, *n* = 3 biological replicates (A–C). *P < 0.05, **P < 0.01, ***P < 0.001, ****P < 0.0001, as assessed by the two‐tailed Student's *t*‐test (A, B) or One‐way ANOVA, *post‐hoc* Bonferroni (C). Source data are available online for this figure.

Next, we used RNA seq. to investigate the presence of endogenous piRNAs in undifferentiated aNPCs (DIF0), or upon onset of vector‐induced neurogenesis (DIF4‐7). To eliminate the possibility of ribosomal RNA (rRNA) or full‐length transfer RNA (tRNA) contaminations, we performed small RNA size selection during library preparation. Following a previously published analysis pipeline (Ghosheh *et al*, 2016), we identified a total of 725,472 putative piRNAs, and using stringent criteria, we focused the subsequent analyses on the 571,439 small noncoding RNAs that perfectly aligned (i.e., no mismatch) with mouse piRNA previously annotated in the piRNA database (piRBase, Zhang *et al*, 2014). Putative piRNAs in aNPCs had a peak length of 30 nt (Fig 2A) and bore a 5′ uridine (U) bias (Fig 2B), in agreement with previous reports in the brain of adult mice (Ghosheh *et al*, 2016). Moreover, the nucleotide‐pair distance probability between the 5′ termini of putative primary and secondary piRNAs was distributed similarly to that of other animals (Gainetdinov *et al*, 2018), with asymptotic convergence around the “0” mark on the abscissa (Fig 2C). Mature piRNAs typically bear 2′‐*O*‐methylation at their 3′ termini, which confers them stability and enables stronger binding to Piwi proteins (Czech *et al*, 2018; Ozata *et al*, 2019). Thereby, we asked whether the endogenous piRNAs isolated from aNPCs were also methylated by evaluating their resistance to periodate oxidation and alkaline ß‐elimination, as previously reported (Kirino & Mourelatos, 2007). As controls, we used a synthetic piRNA bearing or lacking, a 2′‐O‐methylation in its 3′ end. As expected, the unmethylated synthetic piRNA was degraded after sodium periodate treatment, whereas the methylated one was preserved, as indicated by (qPCR)‐based small RNA assay (TaqMan) (Fig 2D). Small RNAs isolated from aNPCs were subject to periodate treatment in the same experiment. As expected, endogenous unmethylated small noncoding RNAs, such as snoRNA‐202 and ‐135, were degraded; whereas four of the most abundant endogenous piRNA‐cluster consensus sequences (piCS, i.e., extended by qPCR primers bearing specificity for shared sequences among different clusters) identified in aNPCs exhibited resistance to periodate treatment, thus indicating their methylation (Fig 2D). We then addressed the Mili‐dependence of the endogenous piRNAs. To this aim, we used three independent strategies to achieve *Mili* KD in aNPCs (Figs 2E and EV1A and B). Specifically, we transduced short‐hairpin RNAs targeting *Mili* transcripts through a lentiviral vector, and transfected two different synthetic antisense oligonucleotides (GapmeRs) targeting distinct *Mili* regions (Fig 4A). Indeed, KD of *Mili* in aNPCs was sufficient to deplete four of the most abundant endogenous piCS (Figs 2F and EV1C), in agreement with the observation that Mili is the main Piwi protein in these cells (Fig 1). Of note, this manipulation did not affect *Miwi* expression, excluding possible compensatory effects on piRNA biogenesis in aNPCs (Fig EV1D). PiRNAs interact with Piwi proteins to form functional complexes. To examine whether endogenous Mili and piRNAs associate in aNPCs, we immunoprecipitated (IP) the Mili protein from differentiating aNPCs (DIF4, i.e., at the peak of Mili expression; the specificity of the anti‐Mili antibody used for the IP was validated by western blotting of lysates from the testis of Mili null and control adult mice, Fig EV1E). IP with IgG was also included as a control for nonspecific binding (Fig 2G). To determine the size distribution of RNAs co‐precipitated with Mili, we used capillary electrophoresis on microfluidic chips. This analysis indicated that the peak size distribution of Mili‐bound RNAs in aNPCs was 25 nt (Fig 2H), in agreement with the known size of Mili‐bound piRNAs in the mouse testis (Ding *et al*, 2017). Real‐time qPCR confirmed that five of the most abundant endogenous piCS identified in aNPCs were enriched in the Mili‐IP compared with the control IP (Fig 2I). Together these results indicate that Mili and piRNAs are co‐expressed and interact in aNPCs.

### Expression of piRNAs parallels Mili abundance in neurogenesis

**Figure 3:**
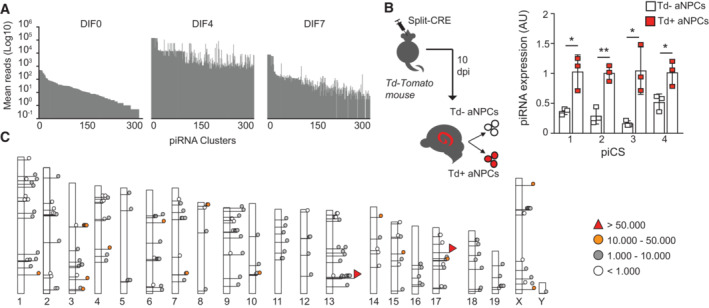
Expression of piRNAs parallels Mili abundance in neurogenesis Mean reads of the piRNA clusters in undifferentiated aNPCs (DIF0) and neuroblasts (DIF4‐7) upon viral‐induced neurogenesis, where the total number of filtered reads in each library ranges between 1.4–4.4 × 10^6^.(left) Schematic representation of the experiment; (right) expression of four of the most abundant piCS in sorted Td^+^ and Td^−^ cells 10 dpi of split‐Cre viruses in hippocampus.Genomic location of the 298 piRNA clusters found in the *Mus musculus* genome (assembly MGSCv37; mm9). Data information: data are expressed as mean ± SEM, *n* = 2 biological replicates (A, C) and *n* = 3 biological replicates (B). *P < 0.05, **P < 0.01, as assessed by the two‐tailed Student's *t*‐test carried out for each piCS between Td^+^ and Td^−^ cells.

**Figure EV2:**
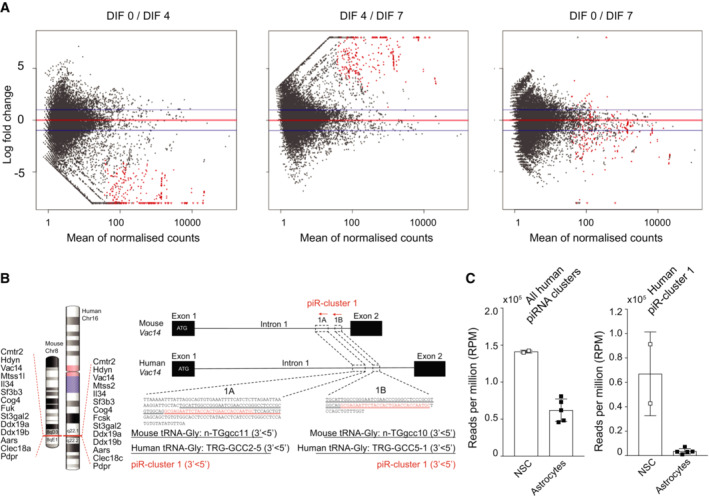
Expression of piRNAs in mouse and human NSC and progeny. Relative to Fig 3 Pairwise comparison of 298 piRNA clusters differentially expressed in undifferentiated aNPCs (DIF0) or neuroblasts upon viral‐induced neurogenesis (DIF4‐7). *n* = 2 biological replicates.Chromosomal location of piR‐cluster 1 in mouse and human; (Right) genomic location and sequences (underlined red text) of piR‐cluster 1 corresponding to tRNAGly genes (underlined black text).Expression of piRNA clusters (left) and piR‐cluster 1 (right) in human NSC and astrocytes. *n* = 2 biological replicates of human NSCs; *n* = 5 biological replicates of human astrocytes. Data information: data in C are expressed as mean ± SEM.

Analysis of small RNAs seq in undifferentiated aNPCs (DIF0), or upon onset of vector‐induced neurogenesis (DIF4‐7) showed that piRNAs were dynamically expressed, peaking at the onset of differentiation (DIF4) (Figs 3A and EV2A), in agreement with the Mili expression pattern (Fig 1). To validate this observation, we sorted Td^+^ NSCs from the adult hippocampus and quantified levels of four of the most abundant piCS, confirming their expression *in vivo* (Fig 3B). Genomic mapping of the piRNA reads from aNPCs and their progeny identified 298 clusters perfectly aligning to the mouse genome (Fig 3C and Dataset EV2). These clusters had an average length of 168 bases, with some exceeding 2,000 bases, as previously seen in mouse testis (Aravin *et al*, 2006; Girard *et al*, 2006). The piRNA raw reads/cluster averaged around 4,700 reads, with two clusters, one located in chromosome 13 and one in the 17, giving rise to more than 80,000 piRNA reads (Fig 3C). Analysis of small RNA seq data for directionality suggested a strand bias, where the majority of the piRNAs arise unidirectionally, although some piRNAs were found to be homologous to both strands and at different loci. Of note, one of the clusters in our dataset (hereafter referred to piR‐cluster 1, Dataset EV2) is homologous to the human piR‐61648 that was recently shown to be selectively expressed in somatic tissues but depleted in gonads (Torres *et al*, 2019; Fig EV2B). In agreement, analysis of small RNA datasets from the RIKEN FANTOM5 project (De Rie *et al*, 2017) showed an enriched expression of the piRNAs bearing sequence homology to piR‐cluster 1, as well as of many additional piRNA clusters in human NSCs compared with differentiated brain cells (Fig EV2C and Dataset EV3). Together, these results indicate that Mili‐dependent piRNAs are more abundant in neural stem/progenitor cells than in their differentiated progeny, thus matching the expression of Mili.

### Depletion of Mili and piRNAs impairs neurogenesis and increases astrogliosis

**Figure 4:**
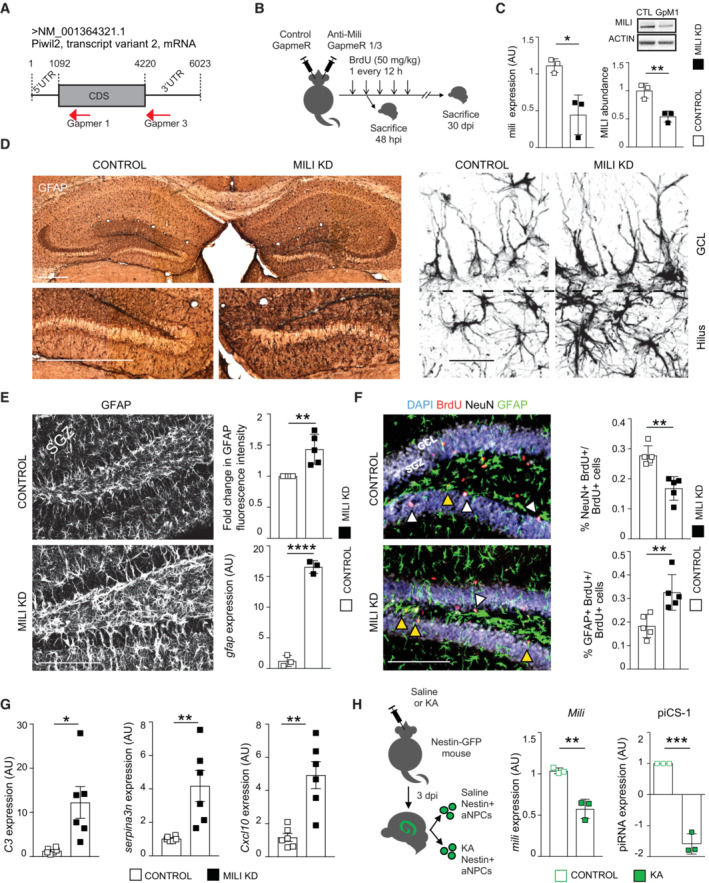
Depletion of Mili and piRNAs impairs neurogenesis and increases astrogliosis Representation of the targeting regions of GapmeR1 and 3 on the *Mili* transcript.Scheme of the *in vivo* experiment.
*Mili* mRNA expression (left bar graph); western blot (inset) and quantification (right bar graph) of Mili protein abundance in lysates from the DG of mouse hippocampi 48 h after the injection of scrambled (Control) or GapmeR1 against *Mili* (Mili KD).Representative light microscopy (left) and confocal (right) micrographs of postnatal hippocampal sections, immunostained for GFAP at 30 dpi of scrambled (Control, left hemisphere) and GapmeR1 against *Mili* (Mili KD, right hemisphere).Representative immunofluorescence micrograph of postnatal hippocampal sections immunostained for GFAP at 30 dpi of scrambled (Control) and GapmeR1 against *Mili* (Mili KD); Right panels: Fold change in GFAP fluorescence intensity level (upper graph) in a hippocampal region of interest (ROI) of 500 μm^2^ in brain slices upon Mili KD compared with Control; *Gfap* mRNA levels (lower graph) in the DG from mouse hippocampi 48 h after the injection of scrambled (Control) or GapmeR1 (Mili KD).(left) Representative immunofluorescence micrograph of postnatal hippocampal sections immunostained for GFAP (green), BrdU (red), NeuN (white), and nuclear DNA (blue) at 30 dpi of scrambled (Control) or GapmeR1 against *Mili* (Mili KD); (right graphs) percentages of NeuN^+^BrdU^+^ (white arrowheads in the images), or GFAP^+^BrdU^+^ (yellow arrowheads in the images) double‐positive cells over total BrdU^+^ cells.Relative mRNAs expression of reactive astrocyte markers in the hippocampus 48 h upon injection of scrambled (control, *n* = 6 mice) or GapmeRs against *Mili* (Mili KD, *n* = 3 mice GapmeR1 and *n* = 3 GapmeR3).(left) Schematic representation of the experiment; *Mili* mRNA (left graph) and piCS1 (right graph) expression in sorted GFP^+^ NSCs from *Nestin*‐GFP mice treated with Saline (Control) or Kainic Acid (KA). Data information: data are expressed as mean ± SEM, *n* = 3 (C, D, E mRNA, H) and 5 (E, F) biological replicates. *P < 0.05, **P < 0.01, ***P < 0.001, ****P < 0.0001, as assessed by the two‐tailed Student's *t*‐test. UTR, untranslated region; CDS, coding sequence; GCL, granular cell layer; SGZ, subgranular zone. The scale bars represent 1 mm (D, left), 10 μm (D, right), and 100 μm (E, F). Source data are available online for this figure.

**Figure EV3:**
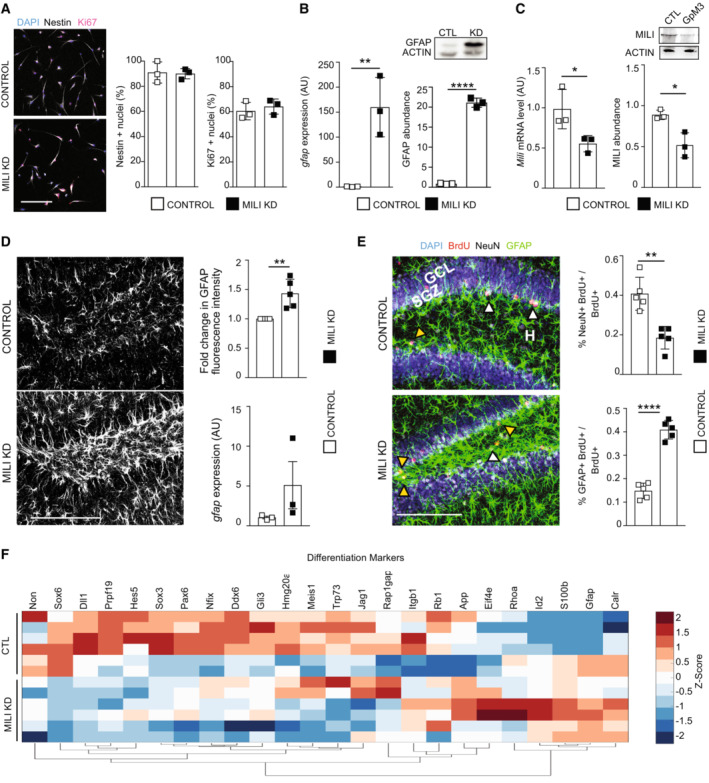
Depletion of Mili and piRNAs does not alter stemness and proliferation and induces the expression of genes involved in astrogliogenesis. Related to Fig 4 (left) Confocal microscopy images of undifferentiated aNPCs transduced *in vitro* with viruses transcribing a Scrambled (Control) and shMILI (Mili KD), immunostained with anti‐Nestin (white), or anti‐Ki67 (purple) antibodies and stained for nuclear DNA with DAPI (blue); (right) Percentage of Nestin or Ki67^+^ cells. over total cells.
*Gfap* mRNA expression (left graph), western blot (inset), and quantification of Gfap protein abundance (right graph) in lysates from control and Mili KD neuroblasts at DIF7.
*Mili* mRNA expression (left graph), western blot (inset), and quantification of Mili protein abundance (right graph) in lysates from mouse hippocampi 48 h after the injection of scrambled (Control) or GapmeR3 against *Mili* (Mili KD).Representative immunofluorescence micrograph of postnatal hippocampal sections immunostained for GFAP; (top right) quantification of GFAP fluorescence intensity level (top) in hippocampal section 30 dpi of GapmeR3 (Mili KD) compared with scrambled (Control). Expression of *Gfap* mRNA (bottom right) in the DG from mouse hippocampi 48 h after the injection of scrambled (Control) or GapmeR3 (Mili KD).(left) Representative immunofluorescence micrograph of postnatal hippocampal sections immunostained for GFAP (green), BrdU (red), NeuN (white), and nuclear DNA (blue) at 30 dpi of scrambled (Control) or GapmeR3 against Mili (Mili KD); (right) percentages of NeuN^+^BrdU^+^ (white arrowheads), or GFAP^+^BrdU^+^ (yellow arrowheads) double‐positive cells over total BrdU^+^ cells.RNA seq. expression data of genes encoding for proteins involved in astrogliogenesis and regulation of neuronal fate in Mili KD neuroblasts at DIF7, compared with Scrambled control. Expression heatmap correlation plots were computed by the k‐means clustering method. Scale bar indicates *Z*‐scores. Data information: data are expressed as mean ± SEM, *n* = 3 (A–C, F) and *n* = 5 (D, E) biological replicates (in F each biological replicate was sequenced with two separate flow cells). *P < 0.05, **P < 0.01, ****P < 0.0001, as assessed by the two‐tailed Student's *t*‐test. GCL, granular cell layer, SGZ, subgranular zone. The scale bars represent 50 μm (A) and 100 μm (D, E). Source data are available online for this figure.

To infer functions of the piRNA pathway in neurogenesis, we KD *Mili* in aNPCs and investigated the consequences of Mili and piRNA depletion for their proliferation, survival, differentiation, and fate. Mili KD did not alter aNPC stemness or proliferation (Fig EV3A), but it led to a dramatic increase in the expression of the astrocyte marker glial fibrillary acidic protein (GFAP) *in vitro* (Fig EV3B). Next, we KD *Mili in vivo* by injecting two different GapmeRs antisense to the *Mili* transcript (GapmeR1 shown in Fig 4; GapmeR3 shown in Fig EV3C), or a scrambled GapmeR (Control) in the DG of postnatal mouse hippocampus (Figs 4A–G and EV3D and E). Inspection of brain sections 30 days after bilateral injections indicated a marked increase in GFAP^+^ cells that showed enlarged somas in the ipsilateral hippocampus injected with GapmeR antisense to *Mili*, compared with the contralateral side injected with control GapmeR (Figs 4D and EV3D). Quantification of GFAP protein (fluorescence intensity) and transcript in Mili KD hippocampus (Figs 4E and EV3D) confirmed this observation. To ascertain whether GFAP^+^ cells were actively generated upon Mili KD, we labeled dividing cells by administration of bromodeoxyuridine (BrdU) in a third cohort of mice, immediately after GapmeRs injection (Figs 4F and EV3E). Thirty days after, we found that Mili KD led to a significant increase in adult‐born GFAP^+^BrdU^+^ glial cells at the expense of NeuN^+^BrdU^+^ neurons (Figs 4F and EV3E). This result was corroborated by RNA seq dataset analysis from differentiating aNPCs *in vitro*, showing an enrichment in the expression of astrocyte‐related genes and a concomitant deregulation of neuronal fate genes upon Mili KD (Fig EV3F). These results indicate that Mili sustains neurogenesis in the postnatal hippocampus, at the expense of gliogenesis. Increased GFAP expression is generally regarded as a hallmark of astrocytic reactivity (Escartin *et al*, 2021). In agreement, we observed a significant increase in the levels of known reactive glial markers (Liddelow *et al*, 2017; Clarke *et al*, 2018) upon Mili KD in the postnatal hippocampus (Fig 4G). To corroborate this result, we took advantage of Kainic Acid (KA) injection in the postnatal hippocampus of mice expressing GFP under the control of the NSCs/NPCs specific promoter *Nestin* (Fig 4H), a treatment previously shown to induce aNSC conversion into reactive glia (Sierra *et al*, 2015; Bielefeld *et al*, 2017). Indeed, this treatment reduced the levels of *Mili* and one of the most abundant piCS‐bearing sequence homology to piR‐cluster 1 in sorted *Nestin*‐GFP^+^ NSCs (Fig 4H). Altogether, these results demonstrate that Mili functions are essential for proper neurogenesis and prevent reactive gliogenesis in the postnatal mouse hippocampus.

### Depletion of Mili and piRNAs in aNPCs results in senescence‐associated phenotypes

**Figure 5:**
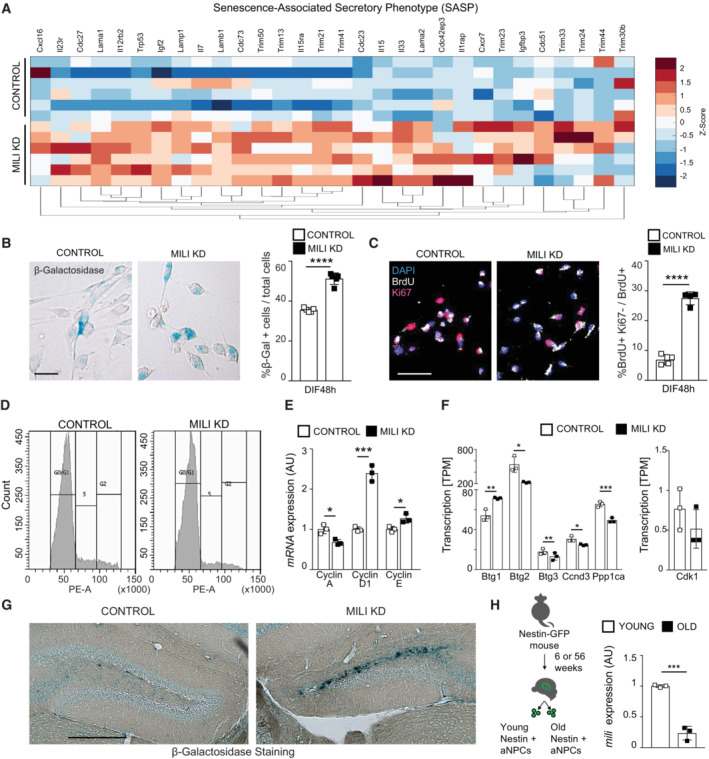
Depletion of Mili and piRNAs in aNPCs results in senescence‐associated phenotypes Heatmap illustrating the expression of genes encoding proteins involved in immune‐modulatory and senescence‐associated phenotype in differentiating neuroblasts (DIF4) upon Mili KD, compared with control cells. Expression heatmap correlation plots were computed by the k‐means clustering method. Scale bar indicates *Z*‐scores.Representative bright‐field microscopy images (left) and quantification (right) of ß‐galactosidase^+^ aNPCs as percent of total cells upon Mili KD, or control 48 h after induction of spontaneous differentiation.Representative fluorescence microscopy images (left) and quantification (right) of control or Mili KD neuroblasts 48 h after spontaneous differentiation, immunostained with anti‐BrdU (white) and Ki67 (purple) antibodies. (Right) Percentage of BrdU^+^ and Ki67^−^ cells over BrdU^+^ cells.Representative cell cycle analysis of propidium iodide staining by flow cytometry in neuroblasts 48 h after spontaneous differentiation; Percentage of cells in G0/G1 and S is reported in the text.Relative mRNA expression of genes encoding proteins involved in the regulation of cell cycle in control and Mili KD neuroblasts 48 h after spontaneous differentiation.Transcript abundance expressed in transcripts per million (TPM) of genes encoding proteins involved in the regulation of cell cycle and differentiation (left) or cell cycle (right) in DIF4 neuroblasts upon Mili KD, compared with control cells.Representative light‐microscopy images of the ß‐galactosidase staining of postnatal hippocampal sections, 30 dpi of scrambled (Control, left hemisphere) and GapmeR1 against *Mili* (Mili KD, right hemisphere). See Fig EV4E for a larger image of this section.(left) Scheme of the *in vivo* experiment; (right) *Mili* mRNA expression in *Nestin*‐GFP^+^ sorted cells from young (6 weeks) and old (56 weeks) mice. Data information: data are expressed as mean ± SEM, *n* = 3 (A, D–F, H) and 5 (B, C) biological replicates (in A each biological replicate was sequenced with two separate flow cells). *P < 0.05, **P < 0.01, ***P < 0.001, ****P < 0.0001, as assessed by the two‐tailed Student's *t*‐test. The scale bars represent 50 μm (B, C) and 500 μm (G).

**Figure EV4:**
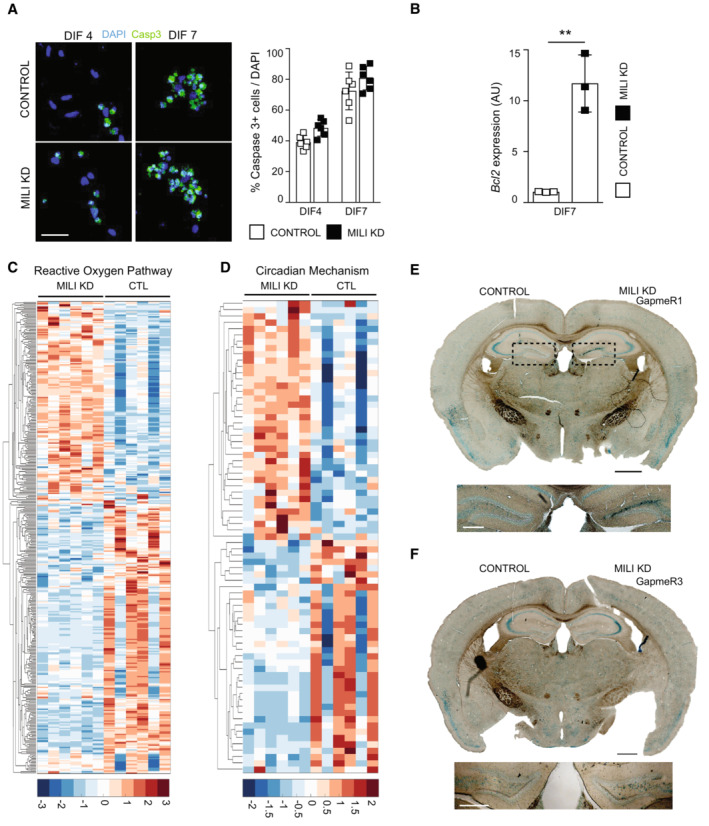
Depletion of Mili and piRNAs does not lead to apoptosis and alters the expression of genes involved in inflammatory responses. Related to Fig 5 ARepresentative fluorescence micrographs of control or Mili KD neuroblasts 4 or 7 days after spontaneous differentiation (DIF4, 7), immunostained with anti‐cleaved caspase‐3 (green) and for nuclear DNA with DAPI (blue). (Right) Percentage of cleaved Caspase‐3^+^ cells over total cells.B
*Bcl2* mRNA expression level in control or Mili KD neuroblasts at DIF7.C, DHeatmap of differentially expressed transcripts in RNA seq from Mili KD neuroblasts, encoding proteins involved in ROS production (C) or circadian regulation (D). Target genes are listed in Dataset EV4. Expression heatmap correlation plots were computed by the k‐means clustering method. Scale bar indicates *Z*‐scores.E, FRepresentative light‐microscopy images of the ß‐galactosidase staining in postnatal hippocampal sections, 30 dpi of scrambled (Control, left hemisphere) and GapmeR1 (E) or GapmeR3 (F) against *Mili* (Mili KD, right hemispheres). Dashed box in E indicates the areas shown in Fig 5. Bottom panels in (E, F) are higher magnification of the hippocampi shown in top panels. Data information: data are expressed as mean ± SEM, *n* = 6 (A) and *n* = 3 (B–D) biological replicates (in C and D each biological replicate was sequenced with two separate flow cells). **P < 0.01, as assessed by the two‐tailed Student's *t*‐test. The scale bars represent 50 μm (A), 1 mm (E, F top) and 500 μm (E, F bottom).

Conversion of hippocampal NSC into reactive glia at the expense of neurogenesis has been related to increased neuroinflammation and cellular senescence (Martín‐Suárez *et al*, 2019; Babcock *et al*, 2021), and it has been observed in normal aging (Clarke *et al*, 2018). The involvement of the piRNA pathway in these mechanisms is unknown. We investigated whether the depletion of Mili and piRNA induces senescence‐associated secretory phenotype (SASP), β‐galactosidase activity (β‐gal) and cell cycle exit, known hallmarks of an aged hippocampal niche (Ahlenius *et al*, 2009; Encinas *et al*, 2011; Jin *et al*, 2021). In agreement with the *in vivo* phenotypes, Mili KD in aNPCs cultures induced a significant increase in the expression of several genes encoding immune‐modulatory and SASP proteins in neuroblasts (DIF4), compared with control cells (Fig 5A). Moreover, Mili KD resulted in a higher proportion of cells positive for β‐gal as early as 48 h upon induction of their spontaneous differentiation (Fig 5B). At the same time point, we immunostained aNPCs with anti‐KI67 (a protein that is expressed in all phases of the cell cycle except G0 and early G1; Yu, 1992) and anti‐BrdU antibodies. Quantification of the BrdU^+^ and KI67^−^ cells over total BrdU^+^ indicated a premature cycle exit upon Mili KD (Fig 5C). Similarly, by propidium iodide incorporation and flow cytometry analysis we found a significant increase in the proportion of cells in G0/G1 phase and a concomitant decrease in S phase cells upon Mili KD (Fig 5D; Mili KD G0/G1 = 89.8%, S = 6.03%, G2/M = 4.03%; Control G0/G1 = 84.7%, S = 8.97% G2/M = 6.27%). Mili KD did not lead to apoptosis (Fig EV4A and B), whereas it led to altered expression of genes encoding proteins associated with oxidative stress, circadian mechanism (Fig EV4C and D, and Dataset EV4), and senescence‐induced cell cycle exit (Fig 5E and F) in agreement with previous reports (Schouten *et al*, 2020; Adusumilli *et al*, 2021; Babcock *et al*, 2021). Strengthening this evidence, we found increased β‐gal activity in the ipsilateral hippocampus injected with different GapmeRs antisense to *Mili*, compared with the contralateral side injected with control GapmeR (Figs 5G and EV4E and F). Next, we sorted *Nestin*‐GFP^+^ NSCs from the DG of young (6 weeks) and ~12 months old mice (i.e., when the majority of hippocampal NSCs turn into an aged phenotype; Martín‐Suárez *et al*, 2019) and found that *Mili* transcript was significantly reduced in aged NSCs compared with the young (Fig 5H). These results indicate that Mili functions likely prevent the senescence of aNPCs and their progeny.

### Identification of piRNA targets in neurogenesis

**Figure 6:**
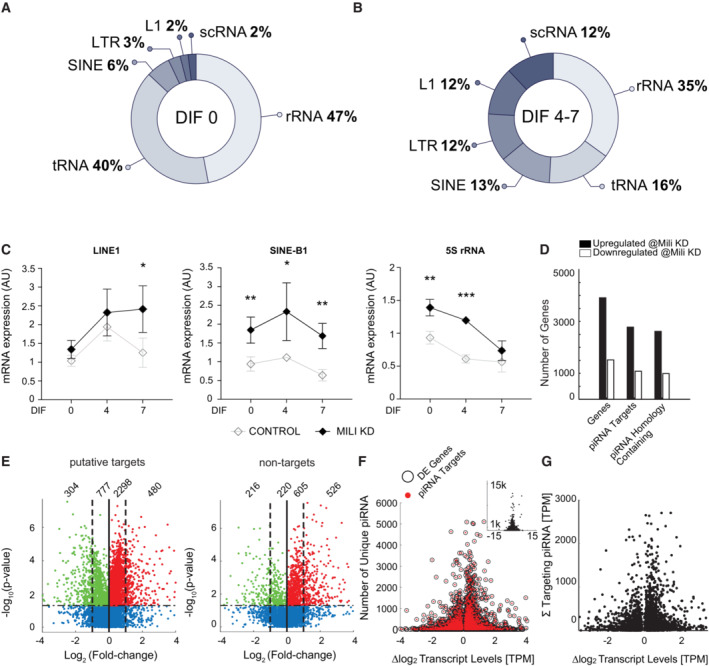
piRNAs target repetitive elements and mRNAs in neurogenesis A, BPie plots showing proportions of noncoding RNAs predicted targets of piRNAs in undifferentiated aNPCs (DIF0) and differentiating neuroblasts (DIF4‐7).CTranscript levels of *LINE1* 5′UTR, *SINEB1*, and *5S rRNA* in the Mili KD or control aNPCs (DIF0) and differentiating neuroblasts (DIF4‐7).DTotal counts of upregulated (black bars) and downregulated protein‐coding genes (white bars) in Mili KD versus scrambled control neuroblasts at DIF4.EVolcano plots showing the log2 fold change of significantly altered mRNA transcripts (numbers of each category indicated) that are target or nontarget of the piRNAs.FThe log2 fold change of significantly altered mRNA transcripts (abscissa) plotted with the raw number of unique piRNA sequences qualified as targeting molecules (ordinate) for all modulated genes (black circles), and piRNA‐targeted genes (red dots), identified by RNA seq. The modulated genes without piRNA target sequences concatenate at the bottom at the “*y* = 0” value; total range, inset.GThe log2 fold change of significantly altered piRNA‐targeted mRNA transcripts (abscissa) plotted with the summed levels of all mRNA‐targeted (complementary) piRNA molecules (ordinate), identified by RNA seq. Data information: data are expressed as mean ± SEM, *n* = 3 (C) biological replicates. Data are expressed in transcripts per million (TPM) as the mean levels of six sequencing runs (three biological replicates sequenced with two separate flow cells) profiles at DIF4 (D–G). Outliers (mean calculation) were detected by more than three mean absolute deviations, for a final “*n*” value between 4 and 6 samples. *P < 0.05, **P < 0.01, ***P < 0.001, as assessed by the two‐tailed Student's *t*‐test. (G) The two‐sample Kolmogorov–Smirnov test on the change in expression between piRNA target and nontarget genes, *P*‐value = 3.21e‐260.

Next, we sought to identify targets of piRNAs in aNPCs lineages. In contrast to germline piRNAs, which primarily target TEs, somatic piRNAs have also homology with, or pair by sequence complementarity, to a variety of noncoding RNAs including tRNAs and others from repetitive elements (Keam *et al*, 2014; Rojas‐Rıós & Simonelig, 2018). Thereby, we first performed *in silico* prediction of noncoding RNAs targeted by the piRNAs identified in our model. Accordingly, TEs were just a minor percentage of the predicted noncoding RNA targets in both undifferentiated aNPCs and progeny (Fig 6A and B), despite their proportion being increased upon induction of neurogenesis (Fig 6B). The latter finding is in agreement with the activation of TEs (e.g., LINE1) observed during neuronal differentiation (Muotri *et al*, 2005; Coufal *et al*, 2009; Upton *et al*, 2015). Interestingly, transcripts from repeats such as 5S rRNA and tRNAs were the main predicted targets in both undifferentiated (47% and 40%, respectively) and progeny (35% and 16%, respectively, Fig 6A and B). To ascertain whether these noncoding RNAs are modulated upon Mili and piRNA depletion, we quantified their levels in Mili KD aNPCs and progeny (Fig 6C). Indeed, Mili depletion significantly elevated levels of 5S rRNA and SINEB1 family of TEs in both undifferentiated aNPCs and progeny, compared with scrambled control (Fig 6C), whereas LINE1, here quantified with a qPCR assay detecting the full‐length transcript, was initially refractory to Mili depletion and its level only increased late in differentiation (Fig 6C).

To identify which protein‐coding transcripts are modulated upon Mili depletion in aNPCs lineages, we analyzed RNA seq data from Mili KD or scrambled control cells during their spontaneous differentiation, at the peak of Mili and piRNA expression (i.e., DIF4). Most of the transcripts modulated upon Mili depletion were upregulated (Fig 6D), and most of them bore sequences antisense to piRNAs and/or harbored homologous sequences to piRNAs (Fig 6D and E). More specifically, we observed that transcripts from individual genes are targeted by multiple unique piRNAs, with a maximum of 11,904 piRNAs targeting a single modulated gene (Fig 6F), and a maximum of 9,870 piRNA transcripts per million (TPM) targeting a single mRNA (Fig 6G).

**Figure 7:**
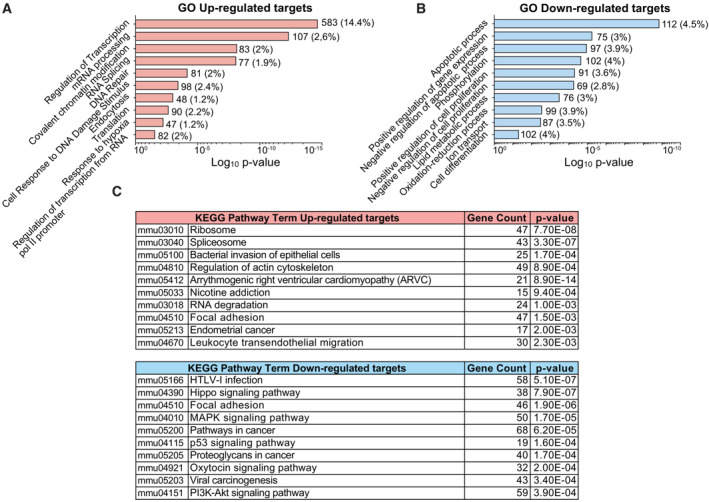
Analysis of genes and pathways modulated upon Mili and piRNA depletion A, BBar graph showing the top biological pathways of significantly upregulated (A) and downregulated (B) protein‐coding genes in Mili KD neuroblasts at DIF4; Numbers in each category indicate gene counts and percentages are normalized on the total number of genes for each category.CMost significant terms generated from KEGG pathway analysis of the modulated targets.

**Figure EV5:**
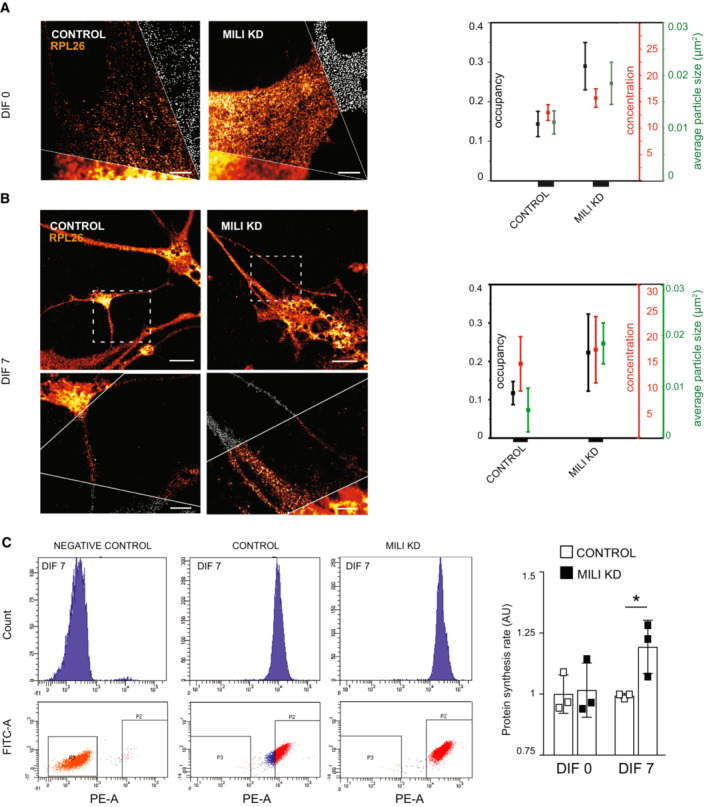
Depletion of Mili and piRNAs enhances polysome assembly and results in higher protein synthesis upon differentiation A, BRepresentative micrographs (Middle cut: g‐STED nanoscopy; Bottom: Confocal; Top: analysis) of control and Mili KD undifferentiated aNPCs (DIF0) and neuroblasts (DIF7) immunostained for the ribosomal protein RPL26. (Right) Normalized distributions of the occupancy, concentration and average particle size of each polyribosome particle in the indicated cells.CProtein synthesis rate (right) as determined by OPP incorporation assay with flow cytometry (left) in control and Mili KD undifferentiated aNPCs (DIF0) and neuroblasts (DIF7). Data information: data are expressed as mean ± SEM, *n* = 3 biological replicates. *P < 0.05, as assessed by the two‐tailed Student's *t*‐test. The scale bars represent 2 μm (A) and 10 μm (B).

To address possible functions of the piRNA targets in aNPCs we searched the Gene ontology (GO) and Kyoto Encyclopedia of Genes and Genomes (KEGG) databases. GO analysis of the upregulated protein‐coding targets upon Mili depletion indicated a prevalence of genes involved in the regulation of chromatin, transcription, mRNA processing, translation, and DNA repair (Fig 7A), which are well‐known functions regulated by the piRNA pathway in both germline and somatic tissues (Czech *et al*, 2018; Rojas‐Rıós & Simonelig, 2018; Ozata *et al*, 2019). Among the downregulated protein‐coding targets, we found genes involved in the regulation of apoptosis, cell proliferation, oxidative pathway, and differentiation (Fig 7B), in agreement with the main phenotypes that we observed upon Mili depletion in neurogenesis. “Ribosome” and “Spliceosome” were the top terms among upregulated genes in the KEGG pathway analysis; whereas, cancer‐related terms were common among the downregulated pathways (Fig 7C), in agreement with the known oncogenic role of Piwil2 in various human tumors (Lee *et al*, 2006). As 5S rRNA and SINEB1, both involved in the control of ribosome biogenesis and translation, are among the piRNA targets in aNPCs and because the dysregulation of ribosome biogenesis has been associated with cellular senescence (Liu & Sabatini, 2020), we investigated ribosome density and translation in Mili KD aNPCs and progeny. Accordingly, Mili depletion increased the density of polyribosomes in both undifferentiated and differentiating aNPCs compared with control cells (Fig EV5A and B), as revealed by immunostaining for the ribosomal protein RPL26 imaged with stimulated emission depletion (STED) nanoscopy (Viero *et al*, 2015). Furthermore, the protein synthesis rate, measured by OPP (O‐propargyl‐puromycin) labeling of nascent proteins, was significantly increased upon Mili depletion in differentiating neuroblasts (DIF7), but not in undifferentiated aNPCs (DIF0) (Fig EV5C), in agreement with the notion that the density of ribosomes over a transcript does not necessarily correlate with its translation (Mills & Green, 2017).

In sum, these results indicate that the piRNA pathway is present and preferentially expressed in adult hippocampal NSC/NPCs compared with their progeny, where it exerts a wide range of gene‐modulatory functions essential for their fitness and neurogenesis.

## Discussion

This study provides the first evidence of the role of the piRNA pathway in neurogenesis. By investigating the presence of Mili and Mili‐dependent piRNAs in aNPCs and by inferring functions of this pathway in the regulation of adult hippocampal neurogenesis, we provide evidence of an essential role for Mili in maintaining NSC fitness and proper fate. This finding adds a new layer of complexity to the understanding of adult brain plasticity and entails implications for aging and neuronal disorders, where dysregulated expression of the piRNA pathway has been reported, such as neurodegeneration (Jain *et al*, 2019; Wakisaka, 2019) and various psychiatric conditions (Page *et al*, 2021).

At the functional level, the Piwi proteins in gonads and bone marrow maintain stem cell pools by preserving their fitness (De Luca *et al*, 2016; Rojas‐Rıós & Simonelig, 2018). Similarly, our data indicate that Mili is required to maintain the fitness of NSC/NPCs in the adult hippocampus. We find that piRNAs are enriched in aNPCs and their expression is dynamic along neurogenesis, mirroring Mili. Moreover, Mili depletion in aNPCs leads to premature cell cycle exit, increases generation of reactive glia, and alters the expression of several inflammatory, reactive oxygen, and circadian‐related genes, which are known hallmarks of an aged hippocampal niche (Bonaguidi *et al*, 2011; Encinas *et al*, 2011; Clarke *et al*, 2018; Martín‐Suárez *et al*, 2019; Schouten *et al*, 2020); accordingly, we find a reduced expression of Mili in hippocampal NSCs of aged mice. In contrast to the dispensable role of Piwi proteins for hematopoiesis (Nolde *et al*, 2013), however, Mili is required for the proper neurogenesis fate. Thereby, our data support the idea that Mili regulates the fate choice in the hippocampal niche, implicating functions of the piRNA pathway in the maintenance of lifelong neurogenesis, possibly to prevent or delay its drift toward reactive gliogenesis. While some of these conclusions require further longitudinal investigations in *Mili* knockout mice (ideally, bearing Lox‐P sites to selectively restrict the deletion of *Mili* to distinct NSC subpopulations), this study underpins a possible involvement of the piRNA pathway in brain plasticity and aging.

At the mechanistic level, several unresolved questions arise from this study. Perhaps the most crucial one is whether Mili functions in neurogenesis are mediated through piRNAs. Indeed, Piwi proteins regulate targets at both the transcriptional and post‐transcriptional levels. However, these mechanisms are very complex and differ according to the cell type, the Piwi proteins involved, effectors, and their subcellular localization and do not necessarily require cleavage‐competent Piwi or piRNAs (Czech *et al*, 2018; Ozata *et al*, 2019). Our data indicate the presence of key players of the piRNA pathway, such as Mili, several cofactors, as well as piRNAs (fulfilling most of the criteria characterizing them, such as length, U‐Bias, 2′‐O‐Methylation at their 3′ ends, inter‐distance, Mili‐dependence and ‐interaction) in aNPCs. We also uncover that this pathway targets, either directly or indirectly, more than 6,000 genes in neurogenesis including repetitive elements, and several genes are known to be regulated by piRNAs in other tissues and cell types; however, given this level of complexity, the mechanisms mediating gene‐regulatory functions of the Mili‐piRNA complexes in our model certainly warrant further investigation. Future exploitation of the “simplified” aNPC model (where Mili is the main Piwi protein, while other cofactors such as the testis‐specific methyl transferase Hen1 are missing), would disentangle the mechanism of piRNA biogenesis and function in neurogenesis, providing the biological understanding of scientific and therapeutic value for brain plasticity and successful aging.

## Materials and Methods

### Reagents and Tools table

**Table:** 

Reagent/resource	Reference or source	Identifier or catalog number
Experimental models		
C57BL/6J (*M. musculus*)	Jackson Lab	B6.129P2Gpr37tm1Dgen/J
Td‐Tomato^flox/wt^ (*M. musculus*)	Jackson Lab	Madisen *et al* (2010)
Mili null mice (*M. musculus*)	European mouse mutant archive (EMMA)	Di Giacomo *et al* (2013)
Nestin‐GFP (*M. musculus*)	C. Fitzsimons' Lab	Mignone *et al* (2004)
Antibodies		
Mouse‐anti‐MILI	Santa Cruz 1:100 WB	Cat # sc‐377347
Mouse‐anti‐MILI	Santa Cruz 1:100 WB, 1:25 IP	Cat # sc‐377258
Rabbit‐anti‐MILI	G. Hannon's Lab 1:150 WB, 1:100 IF/IHC	
Rabbit‐anti‐MIWI	G. Hannon's Lab 1:200 WB	
Rabbit‐anti‐ACTIN	Abcam 1:1,000 WB	Cat # ab13970
Rabbit‐anti‐GADPH	Santa Cruz 1:1,000 WB	Cat # sc‐25778
Rabbit‐anti‐GFAP	Dako 1:1,000 WB, IF/IHC	Cat # Z‐0334
Rat‐anti‐BrdU	Abcam 1:200 IF/IHC	Cat # ab6326
Rabbit‐anti‐KI67	Abcam 1:250 IF/IHC	Cat # ab15580
Mouse‐anti‐NeuN	Millipore 1:250 IF/IHC	Cat # MAB377
Rabbit‐anti‐RPL26	Abcam 1:500 IF/IHC	Cat # ab59567
Mouse‐anti‐Nestin	Millipore 1:250 IF/IHC	Cat # MAB353
Rabbit‐anti‐cleaved caspase‐3	Cell Signaling Technology 1:400 IF/IHC	Cat # 9664
IgG	Millipore‐Sigma 1:100 IP	

### Methods and Protocols

#### Experimental mice

C57BL/6 and Td‐Tomato^flox/wt^ knock‐in reporter mice (Jackson Laboratory stock number 007908) (Madisen *et al*, 2010) and *Mili* null mice (Di Giacomo *et al*, 2013) were housed at *Istituto Italiano di Tecnologia* (IIT); Nestin‐GFP mice (Mignone *et al*, 2004) were housed at the Swammerdam Institute for Life Sciences, University of Amsterdam, The Netherlands. All animal procedures were approved by the IIT animal use committee and the Italian Ministry of health, or by the Commission for Animal Welfare at the University of Amsterdam (DEC protocol 4925, AVD1110020184925), respectively, and conducted in accordance with the Guide for the Care and Use of Laboratory Animals of the European Community Council Directives. All mice were group‐housed under a 12‐h light–dark cycle in a temperature and humidity‐controlled environment with *ad libitum* access to food and water.

#### Virus and GapmeR injection

Split‐cre viruses or GapmeRs injection was done as previously published (Pons‐Espinal *et al*, 2019); briefly, 8 weeks‐old mice Td‐Tomato^flox/wt^ or WT C57BL6/J were anesthetized with isoflurane and 1.5 μl of virus mix (Split‐Cre N‐Cre:C‐Cre), or 1.5 μl of 50 μM antisense GapmeR targeting *Mili* or negative control (custom design probes, MILI 339512, Control 339516, Qiagen), were stereotaxically injected in the dentate gyrus. Bilateral injection of Control GapmeR in the left hemisphere and Mili KD GapmeR in the right hemisphere allowed the analysis of the phenotypes within the same brain. To assess the GapmeRs uptake and the efficacy of *Mili* KD, the first group of mice (*n* = 3 for each GapmeR) was sacrificed 48 h after the injection and the DG and tissue processed for RNA or protein extraction. Another set of animals received 2 BrdU intraperitoneal injections per day for 5 days (50 mg/kg) (one every 12 h) starting 24 h after GapmeRs injection. Animals were sacrificed 30 days after GapmeRs injection (*n* = 5 for each oligo) and tissue processed for histological analysis (Pons‐Espinal *et al*, 2019). GapmeR sequences are listed in the Table EV1.

#### Kainic acid (KA) administration and aNPC collection by fluorescence‐activated cell sorting (FACS)

Kainic acid to elicit tonic, nonconvulsive epileptic seizures, was administered as described before (Bielefeld *et al*, 2019). Briefly, 50 nl of 2.22 mM Kainic Acid dissolved in PBS (pH 7.4) was injected bilaterally into the hippocampus at the following coordinates (AP −2.0, ML +/− 1.5, DV −2.0 mm) (between 9 AM and 1 PM). Control animals were administered saline (pH 7.4). Bilateral dentate gyri from three animals per condition were pooled to allow sufficient recovery of NSC/NPCs. A single‐cell suspension was created using a Neural Tissue Dissociation kit (Miltenyi Biotec), according to the manufacturer's protocol. In order to enrich aNPCs from the DG, we used the endogenous GFP expression driven by the Nestin promotor in combination with FACS. Propidium Iodide (5 μg/ml) was added to the single‐cell suspension to assess cell viability. Cells were sorted using a FACSAriatm III system (BD) with a 488 nm excitation laser. Cell duplets were removed based on forward and side scatters, and viable cells were selected based on PI negativity. GFP‐positive (corrected for autofluorescence) cells were sorted (≅ 50,000 cells/pool) and collected in PBS containing 1% FBS. Trizol LS (Thermo Scientific) was added and after resuspension samples were snap‐frozen and stored at −20°C.

For RNA extraction and cDNA preparation, Td‐Tomato^flox/wt^ or Nestin‐GFP mice were used. Six to ten Td‐Tomato^flox/wt^ mice were euthanized 10 or 30 days after the split‐Cre virus injection. DG cells were dissociated with the Neural Tissue Dissociation Kit P (Miltenyi Biotec) and FACS‐sorted as previously published (Pons‐Espinal *et al*, 2019). FACS‐sorted cells were immediately processed for RNA extraction. Cell cycle length was measured by propidium iodide (PI), which binds to DNA by intercalating between the bases, as previously described (Krishan, 1975). Briefly, cells were trypsinized, resuspended in PBS, and fixed with 70% of ethanol for 40 min on ice. Cells were then centrifuged, resuspended in PBS for 15 min, and then incubated for 1 h at 37°C with 60 μg/ml of PI (Sigma). Cells were collected by centrifuge and resuspended in ice‐cold PBS for FACS analysis.

#### Primary aNPC isolation and culture

Hippocampal NPCs were prepared and expanded as described previously and induction of spontaneous differentiation by growth factor removal was done as previously described (Pons‐Espinal *et al*, 2017, 2019); viral‐induced neuronal differentiation of aNPCs was done by transduction of a viral construct expressing Ascl1‐ERT2 as previously described (Braun *et al*, 2013).

#### Histology, immunofluorescence, and imaging

Immunofluorescence staining on brain slices was performed on sections covering the entire dorsal hippocampus as previously described (Pons‐Espinal *et al*, 2019). Forty micrometer‐tick brain sections were generated using a sliding microtome and were stored in a −20°C freezer as floating sections in 48 well plates filled with cryoprotectant solution (glycerol, ethylene glycol, and 0.2 M phosphate buffer, pH 7.4, 1:1:2 by volume).

To detect Ki67 staining, citrate buffer 10 mM pH = 6 treatment for 10 min at 95°C was used. Secondary fluorescent antibodies were diluted 1:1,000 (Goat Alexa 488, 568, and 647 nm, Invitrogen). Confocal stack images of brain slices (40 μm) were obtained with the Confocal A1 Nikon Inverted SFC with 20× objective (Nikon Instruments, Yokohama, Japan). Cell quantification and analysis were performed using NIS‐Elements software (Nikon Instruments) and the Cell‐counter plugin in Fiji. GFAP intensity fluorescence analysis was done on fluorescence microscopy images acquired by the Confocal A1 Nikon Inverted SFC with 20× objective (Nikon Instruments), with the same parameters for all the sections. Quantification of fluorescence intensity was performed using ImageJ measuring the integrated density of a region of interest (ROI) of 500 μm^2^ for each hippocampal slice. Fold change in fluorescence intensity of Mili KD hippocampi compared with control ones has been plotted in the graph. For all the quantification analysis, six sections were analyzed from each animal, and the mean of the measures from consecutive sections was used for that individual. DAB staining was performed as previously reported (Bielefeld *et al*, 2019). Briefly, sections were incubated with peroxidase block (Vectashield) and permeabilized with 0.3% PBS‐Triton X (PBS‐T) and 0.1% PBS‐T. Sections were blocked with 0.1% PBS‐T and 5% Normal Goat Serum (NGS), incubated with primary antibodies, and subsequently with the corresponding biotinylated secondary antibodies (1:1,000 Goat anti‐rabbit, Invitrogen). Signal amplification was performed using the ABC complex (Vectashield), according to the manufacturer's instructions. Sections were incubated with the solution for DAB reaction (Sigma) and counterstained with Hoechst (1:300), mounted, and coverslipped with Vectashield reagent (VECTOR Labs). ß‐galactosidase detection was obtained with the Senescence Cells Histochemical Staining Kit (Sigma‐Aldrich), according to the manufacturer's instructions. Briefly, cells were plated on coverslip in proliferating medium. Forty‐eight hours after the induction of spontaneous differentiation, cells were washed twice with PBS 1× and incubated with Fixation Buffer 1× for 7 min at RT. Next, cells were rinsed with PBS 1× and incubated with fresh senescence‐associated ß‐Gal (SA‐ß‐Gal) stain solution at 37°C (no CO_2_) for 4 h. Reaction was blocked with PBS 1×, and coverslips were mounted on slides using Vectashield reagent (Vector Labs). Images were obtained using the microscope Nikon Eclipse 80i (Nikon Instruments, Yokohama, Japan) and the percentage of cells expressing ß‐galactosidase was quantified over the number of total cells using a Cell‐counter plugin in Fiji software. To detect ß‐galactosidase *in vivo*, brain slices of 40 μm were collected from perfused animals, and the reaction was carried out in free‐floating sections as previously described for cells. Images (DAB and ß‐galactosidase) were obtained using the microscope Olympus BX51 equipped with Neurolucida software (MBF Bioscience), and a full brain slice was reconstructed from acquired fields. Immunofluorescence staining on cell cultures was performed as reported (Pons‐Espinal *et al*, 2019). To detect BrdU incorporation, cells were pretreated with 2 N HCl for 30 min at 37°C. Cells were mounted in a mounting medium and counterstained with fluorescent nuclear dye DAPI (Invitrogen). Images were obtained using the microscope Nikon Eclipse 80i at 20× or 40× magnification and quantification was performed using a Cell‐counter plugin in Fiji. For the quantification analysis, six fields were analyzed from each coverslip, and the mean of the measures was used for that experimental replica.

#### Mili knockdown (KD) *in vitro*

Adult neural progenitor cells were infected at MOI = 5 with a lentivirus encoding for a *Mili*‐targeted short hairpin (shMILI, plKO.1, Sigma) or for a short hairpin scramble lentivirus (Control, SHC202, Sigma) both decorated with an eGFP reporter. GFP‐positive cells were first selected by FACS after three passages and then plated in proliferating or differentiating media, as previously described. We also performed the knockdown using two different synthetic antisense LNA GapmeRs for Mili KD or negative control (as for the *in vivo*: Mili 339512, Control 339516, Qiagen). Cells were transfected with 150 pmol of GapmeRs 50 μM using Lipofectamin stem transfection reagent (STEM00015, Thermo Fisher) according to the manufacturer's protocol and collected 72 h after treatment. GapmeRs uptake and Mili knockdown were assessed by real‐time qPCR and Western Blot.

#### Protein extraction and Western blot

For total protein extraction, adult testes or hippocampus or cell pellets were homogenized in RIPA buffer and the protein concentration was determined using a Bradford Assay kit (Bio‐Rad). For blot analysis, equal amounts of protein (30 μg) were run on homemade 10% polyacrylamide gels and transferred on nitrocellulose membranes (GE Healthcare). Membranes were probed with the primary antibodies, followed by HRP‐conjugated secondary antibody anti‐rabbit or mouse (Invitrogen, A16104, A16072; 1:2,000). LAS 4000 Mini Imaging System (GE Healthcare) was used to digitally acquire chemiluminescence signals, and the band intensities were quantified using Fiji software (Macbiophotonics) (Schindelin *et al*, 2012).

#### Co‐immunoprecipitation of Mili and piRNA

Adult NPCs were suspended in ice‐cold lysis buffer supplemented with protease inhibitor (Roche) and RNAse inhibitor (Promega) and lysed with a 30G syringe needle. Equal concentrations of cell lysate were incubated overnight at 4°C in rotation with either IgG (Millipore‐Sigma), or the anti‐Mili primary antibody (Santa Cruz, sc‐377258) and UV cross‐linked. Dnase I (Sigma) was added, and the suspensions were incubated with Dynabeads Protein G (Thermo Fisher) to immunoprecipitate the antigen–antibody complex for 2 h at room temperature in rotation. The magnetic beads were captured on a magnetic rack and washed five times each with buffers at increasing salt concentrations. Ten percent of the final wash solution was removed for RNA extraction and subject to real‐time qPCR or analysis with the Bioanalyzer RNA chips (Agilent). The remainder of the beads was captured on a magnetic rack, and the protein content was eluted in RIPA buffer supplemented with 150 mM TCEP, incubated on ice for 10 min, and resolved on a 4–12% polyacrylamide gel. Proteins were transferred and processed for Western blotting as above.

#### RNA extraction and real‐time qPCR

Total RNA was extracted from aNPCs (proliferating and differentiating conditions), or DG dissected from adult C57BL/6, Nestin‐GFP, or Td‐Tomato^flox/wt^ mice with QIAzol protocol (Qiagen) according to the manufacturer's instructions. One microgram of total RNA was treated with DNase I (Sigma) and cDNA was synthesized using iScript cDNA Synthesis kit (Bio‐Rad) or with ImProm‐II reverse transcriptase (Promega). Real‐time qPCR was performed in a duplex with Actin as a reference gene, with QuantiFast SYBR Green PCR Kit (Qiagen) or TaqMan Assay (Thermo Fisher) on ABI‐7500 Real‐Time PCR System (Applied Biosystems). Expression levels were determined relative to Actin, using the delta‐delta Ct method. Primers were designed using NCBI/UCSC Genome Browser and Primer3 software tools and then checked in PrimerBLAST for their specificity to amplify the desired genes. For piRNA real‐time qPCR, total RNA enriched in the fraction of small RNAs was extracted using miRNeasy Mini kit (Qiagen) following the manufacturer's instructions from aNPCs, microdissected DG from hippocampi of C57BL6/J or Td‐Tomato^flox/wt^ mice. cDNA was obtained using the TaqMan MicroRNA Reverse Transcription Kit (Thermo Fisher) according to the manufacturer's instructions and quantified using the Custom TaqMan Small RNA Assay (Thermo Fisher) on a ABI‐7500 Real‐Time PCR System (Applied Biosystems). Each sample was normalized to U6 snRNA level (Thermo Fisher). Oligonucleotide sequences are listed in the Table EV1.

#### RNA library preparation

For small RNA libraries, the quantity and quality of the total RNA isolated from aNPCs/neuroblasts cultures were measured by Nanodrop spectrophotometer (Thermo Fisher) and Experion RNA chips (Bio‐Rad). RNA with RNA integrity number (RIN) values ≥ 9.5 were selected for the study. One microgram of high‐quality RNA for each sample was used for library preparation according to the Illumina TruSeq small RNA library protocol (Illumina Inc., CA). Briefly, 3′ adapters were ligated to 3′ end of small RNAs using a truncated RNA ligase enzyme followed by 5′ adaptor ligation using an RNA ligase enzyme. Reverse transcription followed by PCR was used to prepare cDNA using primers specific for the 3′ and 5′ adapters. The amplification of those fragments having adapter molecules on both ends was carried out with 13 PCR cycles. The amplified libraries were pooled together and run on a 6% polyacrylamide gel. The 145–160 bp bands (which correspond to inserts of 24–32 nt cDNAs) were extracted and purified using the Wizard® SV Gel and PCR Clean‐Up System (Promega). The quality of the library was assessed by the Experion DNA 1 K chips (Bio‐Rad). Small RNA sequencing using HiSeq2000 (Illumina Inc., CA) was performed by the IIT genomics facility at the Center for Genomic Science (IIT@SEMM, Milan, Italy). For Long RNA Libraries, quantity and quality of the total RNA extracted from WT/Mili KD aNPCs were measured by Qubit 4 Fluorometer (Thermo Fisher) and Bioanalyzer RNA chips (Agilent). RNA with RIN values ≥ 8 were selected for the study. Thirty nanogram of high‐quality RNA for each sample was used for library preparation according to the Illumina Stranded Total RNA Prep, Ligation with Ribo‐Zero Plus Kit (20040529, Illumina Inc., CA) using the IDT for Illumina Indexes Set A (20040553). Briefly, after ribosomal RNA depletion, RNA was fragmented, denatured and cDNA synthesized. The 3′ ends were adenylated and anchors ligated. After amplification and clean‐up, the quality of the libraries was assessed by the Bioanalyzer DNA chips (Agilent). Paired‐End stranded total RNA sequencing on NovaSeq 6000 Sequencing System instrument (Illumina Inc., CA), was performed by the IIT Genomic facility at the Center for Human Technologies, Genoa, Italy. Sequencing was performed bidirectionally, and in duplicate by two flow cell pairs of 100 and 150 base pairs, for a total of six measurements produced from three independent samples for each differentiation time point and genotype.

#### Small RNA sequencing data processing

Was done essentially as previously published (Ghosheh *et al*, 2016). Briefly, Illumina reads were trimmed to remove the 3′ adapter using Cutadapt, with parameters ‐m 25 ‐q 20. Since piRNA size ranges from 26 to 31 bases, all sequences with length ≤ 24 bases were discarded. Reads mapped to known noncoding RNAs (RNAcentral v6.0 snoRNA, UCSC tRNA, miRBase Release 21 miRNA hairpin and mature miRNA annotation, NCBI complete ribosomal DNA unit) were removed from the datasets. The comparison was performed using NCBI BLASTN v2.6.0 with parameters ‐max_hsps = 1, ‐max_target_seqs = 1, ‐perc_identity = 80, mismatches <= 1, qcovhsp >= 90. Reads were aligned on the nonrepeat‐masked UCSC release 9 of the mouse genome (MM9) using *bowtie* (Langmead & Salzberg, 2012) v2.2.6 with the sensitive preset option and allowed a maximum of 100 alignments. All the reads that aligned to the genome were retained and used for subsequent analysis. piRNA clusters were identified collapsing overlapped piRNA sequences (piRBase Release 1; Zhang *et al*, 2014) into one cluster (mergeBed with preset options; Quinlan & Hall, 2010). piRNA clusters and all the reads that aligned to the genome were intersected (intersectBed with option ‐f 1). Intersection files were then parsed using a custom perl script in order to evaluate alignment counts. Differential expression was assessed using DESeq2 (Love *et al*, 2014). piRNA clusters were considered differentially expressed when the adjusted *P*‐value was ≤0.05, and down‐ and up‐regulation was established in the range of ≤ −1 to ≥ 1 log2 fold change, respectively. piRNA sequences were then categorized for the putative mRNA transcript targets (for each gene). In order to obtain a count of piRNA target levels, which target individual gene transcripts, for each differentiation time point (DIF0‐7), piRNA transcripts were expressed in transcripts per million (TPM) and summed for each target category. Spearman correlation was performed between the levels of the piRNA in the Sh‐Scramble (control) genotype and compared with the fold‐change level of the putative target genes, which were found to be significantly altered (up and down) in the Sh‐Mili‐KD genotype. In order to assess the clustering behavior of putative piRNAs, the 5′ termini positions of each cluster‐associated putative primary and putative secondary piRNA sequences were analyzed for distance, represented as probability, within a range of 200 nucleotides in the 5′ direction and 200 nucleotides in the 3′ direction of the putative primary piRNAs, as reported previously (Gainetdinov *et al*, 2018). The positional distance between piRNAs for each cluster was sampled iteratively for each assigned piRNA and normalized by the total number of diverse piRNAs associated with each cluster. The distance probability distribution was assayed by the locally weighted smoothing linear regression method (LOWESS), by using the built‐in MATLAB “fit” function (MathWorks, Natick, MA), with a span value of 0.1.

#### mRNA sequencing data processing

Adapter sequences were trimmed using *Cutadapt*, after which a quality control trim was implemented on a sliding window of 25 nucleotides, for 2 base pairs with a minimum quality score of 26, with the *Bowtie* build for the MATLAB bioinformatics suite (MathWorks, Natick, MA). Transcript quantification was performed with the Salmon suite (Patro *et al*, 2017), on the NCBI mouse genome, release 67, obtained from the ENSEMBL FASTA directory. The identified ENSEMBL gene accessions were grouped for the different transcript reads, and the read counts, expressed in TPM, were summed for the annotated transcripts. Outlier reads for each gene transcript level in TPM were detected by the mean absolute deviations method (MAD), where reads with more than 3 scaled MAD distances from the mean were eliminated from statistical analysis. Then, the mean and SD for each gene were used for statistical analysis among the different genotypes by one‐way ANOVA with multiple comparisons. *P*‐values lower than, or equal to, 0.05 were selected as the threshold of significance, for a minimum count of 4 of 6 samples per gene. Database for Annotation, Visualization and Integrated Discovery (DAVID, https://david.ncifcrf.gov/) (Huang *et al*, 2009), was used to perform Gene ontology (GO) and Kyoto Encyclopedia of Genes and Genomes (KEGG) signaling pathway analysis using whole *Mus musculus* genome as background. Expression heatmap correlation plots were computed by the k‐means clustering method, with imputation performed by the nearest‐neighbor method by the MATLAB “clustergram” function (MathWorks, Natick, MA, USA), as described previously (Eisen *et al*, 1999).

#### Periodate oxidation/alkaline ß‐elimination

Periodate oxidation and alkaline ß‐elimination were performed as previously described (Balaratnam *et al*, 2018). Briefly, total small RNA fractions were collected from 8 × 10^6^ aNPCs (per replica). Two portions, each containing 25 μg of small RNA were dissolved in 87.5 μl 0.06 M borate buffer (pH 8.6). Then, 12.5 μl of nuclease‐free water (to control group) or 200 mM sodium periodate (to treatment group) were added to the reaction and samples were incubated for 1 h at room temperature. After the incubation, the reaction was stopped by adding 10 μl of glycerol for another 30 min. For the control group (treated with water), 12.5 μl sodium periodate was incubated with glycerol for 60 min prior to adding to the samples to maintain the same ion strength between the control and treatment. RNA was then precipitated by ethanol precipitation method 1 h at −80°C. Precipitated RNA was dissolved in 100 μl of 0.055 M borate buffer (pH 9.5) and incubated for 60 min at 45°C. RNA was precipitated again as before, washed, and used for TaqMan small RNA assay. As an internal control for the assay, a synthetic piRNA sequence (corresponding to one of the most abundant piRNA found in aNPCs bearing homology to piR‐cluster‐1) either modified with the 3′‐end 2′‐O‐methylation (positive control) or bearing a terminal 2′,3′‐hydroxyl group (negative control) were subject to the periodate treatment as above.

#### Protein synthesis assay

To quantify the protein synthesis rate of cells, we used the Global Protein Synthesis Assay Kit (FACS/Microscopy) and Red Fluorescence kit (Abcam), following the manufacturer's instructions. Briefly, cells in proliferating or differentiating media (DIF7) were treated with Cycloheximide as an inhibitor of protein synthesis, for 30 min at 37°C. Media were replaced with fresh aliquots containing Protein Label (400×) diluted to 1× final concentration and the cells were incubated for additional 30 min at 37°C. Negative control cells were not incubated with the protein label. Samples were analyzed by FACS for red fluorescence generated by *de novo* synthesized protein during click reaction. Translation rate is directly proportional to emitted fluorescence. Cells emitting fluorescence lower than 10^3^ were considered negative (P3) and higher than 10^4^ were considered positive (P2).

#### Immunofluorescence, STED nanoscopy, and particle analysis

Confocal and Stimulated Emission Depletion (STED) nanoscopy were performed as previously reported (Vicidomini *et al*, 2018; Diaspro & Bianchini, 2020). aNPCs were plated on glass coverslips 24 h before fixation. Cells were fixed with PFA 4%, permeabilized with PBS‐T 0.1%, blocked for 1 h at room temperature with PBS‐T 0.1% NGS 5%, and incubated according to the dilution suggested by the manufacturer's instructions with 0.01 μg/ml rabbit polyclonal antibody against the N terminus of RPL26 (Abcam) for 1 h at room temperature. Cells were washed extensively and incubated with the secondary antibody goat anti‐rabbit ATTO‐647N (0.8 μg/ml; Sigma) for 45 min. Nuclei were stained while mounting the coverslip with DAPI‐Prolong antifade (Invitrogen). Confocal and STED images were acquired at 23°C with a modified TCS SP5 STED‐CW gated and operated with its own imaging software, LAS X (Leica Microsystems, Mannheim, Germany). The microscope has been customized with a second pulsed STED laser line at 775 nm. The beam originates from a Onefive Katana HP 8 (NKT, Birkerød, Denmark) and passes through a vortex phase plate (RPC photonics, Rochester, NY, USA) before entering the microscope through the IR port. The depletion laser pulses are electronically synchronized with the Leica's supercontinuum pulsed and visible excitation laser. The ATTO‐647N fluorescence was excited at 633 nm, and the fluorescence depletion was performed at 775 nm. The maximal focal power of the STED beam was 200 mW at 80 MHz. Both beams were focused into the 1.4 NA objective lens (HCX PL APO 100× 1.40 NA Oil STED Orange; Leica). Fluorescence was collected by the same lens, filtered with a 775 nm notch filter, and imaged in the spectral range 660–710 nm by the hybrid detector with a time gating of 1 ns. All the images have a 14 nm pixel size and 37‐μs pixel dwell time. The analysis of polysome clusters in aNPC lineages was performed on more than 20 images likewise different cells. Image analysis was performed using the Fiji software.

#### *In silico* piRNA targets prediction

For piRNA targets analysis, we divided the sequencing data into one set of 100 piRNA clusters enriched in proliferating aNPCs (DIF0) and a second set of 198 clusters specifically expressed at DIF4/7 stage. The Differential Expression analysis for piRNAs mapping on repeat elements (REs) in DIF4 and DIF7 compared with DIF0 was done using EdgeR software package (Robinson & Oshlack, 2010). Identification of piRNA targets was divided into: piRNAs mapping on REs only/piRNAs mapping on GENCODE elements/piRNAs mapping on REs within GENCODE elements/unannotated piRNAs/piRNAs clusters. Gene Ontology analysis for piRNAs mapping on GENCODE protein‐coding genes (but NOT mapping on REs) has been done with the R package GOFuncR (https://bioconductor.org/packages/release/bioc/html/GOfuncR.html).

#### Quantification and statistical analysis

Data are presented as mean ± SEM and were analyzed using Prism 6 (GraphPad). Statistical significance was assessed with a two‐tailed unpaired *t*‐test for two experimental groups. For experiments with three or more groups, one‐way ANOVA with the Bonferroni's multiple comparison test was used. Results were considered significant when *P* < 0.05. The number of samples (*n*) in each group is reported in the figure legend. Exact *P*‐values of the experiments are shown in Table 1.

**Table 1:** Exact *P*‐values of the experiments presented in the paper.

Figure	Comparison	*P*‐value	Statistical test
1B	DIF0/DIF7	**P = 0.0022	One‐way ANOVA, *post‐hoc* Bonferroni
	DIF0/DIF14	***P = 0.0001	
	DIF4/DIF7	***P = 0.0003	
	DIF4/DIF14	****P < 0.0001	
1C	Testis/Hippocampus	***P = 0.0004	One‐way ANOVA, *post‐hoc* Bonferroni
	Testis/aNPCs	***P = 0.0005	
1D	Testis/Hippocampus	***P = 0.0002	One‐way ANOVA, *post‐hoc* Bonferroni
	Testis/aNPCs	**P = 0.0013	
1E	Neurons/aNPCs	***P = 0.0008	Two‐tailed Student's *t*‐test
1G	Td^−^/Td^+^ 10 dpi	*P = 0.0224	One‐way ANOVA, *post‐hoc* Bonferroni
	Td^+^ 30/10 dpi	*P = 0.0355	
2E	mRNA Ctl/Mili KD	*P = 0.014	Two‐tailed Student's *t*‐test
	WB Ctl/Mili KD	**P = 0.0015	
2F	piCS1 Ctl/Mili KD	***P = 0.0004	Two‐tailed Student's *t*‐test
	piCS2 Ctl/Mili KD	***P = 0.0005	
	piCS3 Ctl/Mili KD	***P = 0.0002	
	piCS4 Ctl/Mili KD	****P < 0.0001	
2I	piCS2 anti‐Piwil2/IgG	^ns^ *P* = 0.1018	Two‐tailed Student's *t*‐test
	piCS3 anti‐Piwil2/IgG	*P = 0.0423	Two‐tailed Student's *t*‐test
	piCS4 anti‐Piwil2/IgG	*P = 0.0142	Two‐tailed Student's *t*‐test
	piCS5 anti‐Piwil2/IgG	**P = 0.0097	Two‐tailed Student's *t*‐test
3B	piCS1 Td^−^/Td^+^	*P = 0.0164	Two‐tailed Student's *t*‐test
	piCS2 Td^−^/Td^+^	**P = 0.0028	
	piCS3 Td^−^/Td^+^	*P = 0.0184	
	piCS4 Td^−^/Td^+^	*P = 0.0278	
4C	mRNA Ctl/Mili KD	*P = 0.0152	Two‐tailed Student's *t*‐test
	WB Ctl/Mili KD	**P = 0.0083	
4E	GFAP Ctl/Mili KD	**P = 0.0061	Two‐tailed Student's *t*‐test
	mRNA Ctl/Mili KD	****P < 0.0001	
4F	NeuN Ctl/Mili KD	**P = 0.0013	Two‐tailed Student's *t*‐test
	GFAP Ctl/Mili KD	**P = 0.0077	
4G	C3 Ctl/Mili KD	*P = 0.0124	Two‐tailed Student's *t*‐test
	Serpin Ctl/Mili KD	**P = 0.0068	
	Cxcl10 Ctl/Mili KD	**P = 0.0013	
4H	Mili Ctl/KA	**P = 0.0028	Two‐tailed Student's *t*‐test
	piCS1 Ctl/KA	***P = 0.0002	
5B	β‐Gal Ctl/Mili KD	****P < 0.0001	Two‐tailed Student's *t*‐test
5C	BrdU Ki67 Ctl/Mili KD	****P < 0.0001	Two‐tailed Student's *t*‐test
5D	G0‐G1 Ctl/Mili KD	*P = 0.0186	Two‐tailed Student's *t*‐test
	S Ctl/Mili KD	**P = 0.0056	
5E	Cyclin A Ctl/Mili KD	*P = 0.0132	Two‐tailed Student's *t*‐test
	Cyclin D1 Ctl/Mili KD	***P = 0.0003	
	Cyclin E Ctl/Mili KD	*P = 0.0266	
5F	Btg1 Ctl/Mili KD	**P = 0.0068	Two‐tailed Student's *t*‐test
	Btg2 Ctl/Mili KD	*P = 0.0174	
	Btg3 Ctl/Mili KD	**P = 0.0068	
	Ccnd3 Ctl/Mili KD	*P = 0.0279	
	Ppp1ca Ctl/Mili KD	***P = 0.0005	
5H	Mili young/old	***P = 0.0003	Two‐tailed Student's *t*‐test
6C	SINE DIF0 Ctl/Mili KD	**P = 0.0079	One‐way ANOVA, *post‐hoc* Bonferroni
	SINE DIF4 Ctl/Mili KD	*P = 0.0405	
	SINE DIF7 Ctl/Mili KD	**P = 0.0059	
	rRNA DIF0 Ctl/Mili KD	**P = 0.0074	
	rRNA DIF4 Ctl/Mili KD	***P = 0.0002	
6D	LINE1 DIF7 Ctl/Mili KD	*P = 0.00472	Two‐tailed Student's *t*‐test
6G	mRNA Target Mili KD/CtlmRNA Nontarget Mili KD/Ctl	*P* = 3.21E‐260	Two‐sample Kolmgorov‐Smirnov test
EV1A	mRNA Ctl/GpM1	*P = 0.0133	Two‐tailed Student's *t*‐test
	mRNA Ctl/GpM3	*P = 0.038	
EV1B	WB Ctl/GpM1	***P = 0.001	
	WB Ctl/GpM3	*P = 0.014	
EV1C	piCS Ctl/GpM1/GpM3	****P < 0.0001	One‐way ANOVA, *post‐hoc* Bonferroni
EV3B	mRNA Ctl/Mili KD	**P = 0.098	Two‐tailed Student's *t*‐test
	WB Ctl/Mili KD	****P < 0.0001	
EV3C	mRNA Ctl/Mili KD	*P = 0.0477	Two‐tailed Student's *t*‐test
	WB Ctl/Mili KD	*P = 0.017	
EV3D	GFAP Ctl/Mili KD	**P = 0.0039	Two‐tailed Student's *t*‐test
EV3E	NeuN Ctl/Mili KD	**P = 0.012	Two‐tailed Student's *t*‐test
	GFAP Ctl/Mili KD	****P < 0.0001	
EV4B	Bcl2 Ctl/Mili KD	**P = 0.098	Two‐tailed Student's *t*‐test
EV5C	DIF7 Ctl/Mili KD	*P = 0.0338	One‐way ANOVA, *post‐hoc* Bonferroni

## Author contributions

**Caterina Gasperini:** Conceptualization; data curation; formal analysis; investigation; visualization; methodology; writing – original draft; writing – review and editing. **Kiril Tuntevski:** Data curation; formal analysis; investigation; visualization; methodology; writing – review and editing. **Silvia Beatini:** Data curation; formal analysis; investigation; visualization; methodology; writing – review and editing. **Roberta Pelizzoli:** Data curation; formal analysis; investigation; methodology. **Amanda Lo Van:** Data curation; formal analysis; visualization. **Damiano Mangoni:** Data curation; formal analysis; investigation; visualization; methodology; writing – review and editing. **Rosa M Cossu:** Data curation; formal analysis; investigation; visualization; methodology; writing – review and editing. **Giovanni Pascarella:** Data curation; software; formal analysis; investigation; visualization; methodology; writing – review and editing. **Paolo Bianchini:** Formal analysis; investigation; visualization; methodology; writing – review and editing. **Pascal Bielefeld:** Formal analysis; investigation; methodology; writing – review and editing. **Margherita Scarpato:** Formal analysis; methodology. **Meritxell Pons‐Espinal:** Supervision; methodology; writing – review and editing. **Remo Sanges:** Software; formal analysis; supervision; methodology; writing – review and editing. **Alberto Diaspro:** Supervision; funding acquisition; methodology; writing – review and editing. **Carlos P Fitzsimons:** Formal analysis; supervision; funding acquisition; methodology; writing – review and editing. **Piero Carninci:** Supervision; funding acquisition; methodology; writing – review and editing. **Stefano Gustincich:** Supervision; funding acquisition; methodology; writing – review and editing. **Davide De Pietri Tonelli:** Conceptualization; data curation; formal analysis; supervision; funding acquisition; investigation; visualization; methodology; writing – original draft; project administration; writing – review and editing.

## Disclosure and competing interests statement

The authors declare that they have no conflict of interest.

## Supporting information



Expanded View Figures PDF

Table EV1

Dataset EV1

Dataset EV2

Dataset EV3

Dataset EV4

Source Data for Expanded View

PDF+

Source Data for Figure 1

Source Data for Figure 2

Source Data for Figure 4

## Data Availability

Mouse Small RNA sequencing data have been deposited in the European Nucleotide Archive (ENA) under the accession: PRJEB40241 (https://www.ebi.ac.uk/ena/browser/view/PRJEB40241?show=reads) and the long RNA sequencing data in the Gene Expression Omnibus (GEO) database under the accession GSE182848 (https://www.ncbi.nlm.nih.gov/geo/query/acc.cgi?acc=GSE182848); human datasets (De Rie *et al*, 2017) are available through RIKEN FANTOM5.

## References

[embr202153801-bib-0001] Adusumilli VS , Walker TL , Overall RW , Klatt GM , Zeidan SA , Zocher S , Kirova DG , Ntitsias K , Fischer TJ , Sykes AM *et al* (2021) ROS dynamics delineate functional states of hippocampal neural stem cells and link to their activity‐dependent exit from quiescence. Cell Stem Cell 28: 300–314 33275875 10.1016/j.stem.2020.10.019 PMC7875116

[embr202153801-bib-0002] Ahlenius H , Visan V , Kokaia M , Lindvall O , Kokaia Z (2009) Neural stem and progenitor cells retain their potential for proliferation and differentiation into functional neurons despite lower number in aged brain. J Neurosci 29: 4408–4419 19357268 10.1523/JNEUROSCI.6003-08.2009 PMC6665731

[embr202153801-bib-0003] Altman J (1962) Are new neurons formed in the brains of adult mammals? Science 135: 1127–1128 13860748 10.1126/science.135.3509.1127

[embr202153801-bib-0004] Aravin A , Gaidatzis D , Pfeffer S , Lagos‐Quintana M , Landgraf P , Iovino N , Morris P , Brownstein MJ , Kuramochi‐Miyagawa S , Nakano T *et al* (2006) A novel class of small RNAs bind to MILI protein in mouse testes. Nature 442: 203–207 16751777 10.1038/nature04916

[embr202153801-bib-0005] Babcock KR , Page JS , Fallon JR , Webb AE (2021) Adult hippocampal neurogenesis in aging and Alzheimer's disease. Stem Cell Reports 16: 1–13 33636114 10.1016/j.stemcr.2021.01.019 PMC8072031

[embr202153801-bib-0006] Balaratnam S , West N , Basu S (2018) A piRNA utilizes HILI and HIWI2 mediated pathway to down‐regulate ferritin heavy chain 1 mRNA in human somatic cells. Nucleic Acids Res 46: 10635–10648 30102404 10.1093/nar/gky728 PMC6237762

[embr202153801-bib-0007] Bielefeld P , Sierra A , Encinas JM , Maletic‐Savatic M , Anderson A , Fitzsimons CP (2017) A standardized protocol for stereotaxic intrahippocampal administration of kainic acid combined with electroencephalographic seizure monitoring in mice. Front Neurosci 11: 1–9 28405182 10.3389/fnins.2017.00160 PMC5370320

[embr202153801-bib-0008] Bielefeld P , Schouten M , Meijer GM , Breuk MJ , Geijtenbeek K , Karayel S , Tiaglik A , Vuuregge AH , Willems RAL , Witkamp D *et al* (2019) Co‐administration of anti microRNA‐124 and ‐137 oligonucleotides prevents hippocampal neural stem cell loss upon non‐convulsive seizures. Front Mol Neurosci 12: 1–13 30837840 10.3389/fnmol.2019.00031 PMC6389789

[embr202153801-bib-0009] Bonaguidi MA , Wheeler MA , Shapiro JS , Stadel RP , Sun GJ , Ming GL , Song H (2011) *In vivo* clonal analysis reveals self‐renewing and multipotent adult neural stem cell characteristics. Cell 145: 1142–1155 21664664 10.1016/j.cell.2011.05.024 PMC3124562

[embr202153801-bib-0010] Braun SMG , Machado RAC , Jessberger S (2013) Temporal control of retroviral transgene expression in newborn cells in the adult brain. Stem Cell Reports 1: 114–122 24052947 10.1016/j.stemcr.2013.06.003 PMC3757750

[embr202153801-bib-0011] Clarke LE , Liddelow SA , Chakraborty C , Münch AE , Heiman M , Barres BA (2018) Normal aging induces A1‐like astrocyte reactivity. Proc Natl Acad Sci USA 115: E1896–E1905 29437957 10.1073/pnas.1800165115 PMC5828643

[embr202153801-bib-0012] Coufal NG , Garcia‐Perez JL , Peng GE , Yeo GW , Mu Y , Lovci MT , Morell M , O'Shea KS , Moran JV , Gage FH (2009) L1 retrotransposition in human neural progenitor cells. Nature 460: 1127–1131 19657334 10.1038/nature08248 PMC2909034

[embr202153801-bib-0013] Czech B , Munafò M , Ciabrelli F , Eastwood EL , Fabry MH , Kneuss E , Hannon GJ (2018) piRNA‐guided genome defense: from biogenesis to silencing. Annu Rev Genet 52: 131–157 30476449 10.1146/annurev-genet-120417-031441 PMC10784713

[embr202153801-bib-0014] De Luca L , Trino S , Laurenzana I , Simeon V , Calice G , Raimondo S , Podestà M , Santodirocco M , Di Mauro L , La Rocca F *et al* (2016) MiRNAs and piRNAs from bone marrow mesenchymal stem cell extracellular vesicles induce cell survival and inhibit cell differentiation of cord blood hematopoietic stem cells: a new insight in transplantation. Oncotarget 7: 6676–6692 26760763 10.18632/oncotarget.6791 PMC4872742

[embr202153801-bib-0015] De Rie D , Abugessaisa I , Alam T , Arner E , Arner P , Ashoor H , Åström G , Babina M , Bertin N , Burroughs AM *et al* (2017) An integrated expression atlas of miRNAs and their promoters in human and mouse. Nat Biotechnol 35: 872–878 28829439 10.1038/nbt.3947 PMC5767576

[embr202153801-bib-0016] Di Giacomo M , Comazzetto S , Saini H , DeFazio S , Carrieri C , Morgan M , Vasiliauskaite L , Benes V , Enright AJ , O'Carroll D (2013) Multiple epigenetic mechanisms and the piRNA pathway rnforce LINE1 dilencing during adult spermatogenesis. Mol Cell 50: 601–608 23706823 10.1016/j.molcel.2013.04.026

[embr202153801-bib-0017] Diaspro A , Bianchini P (2020) Optical nanoscopy. Riv Nuovo Cim 43: 385–455

[embr202153801-bib-0018] Ding D , Liu J , Dong K , Midic U , Hess RA , Xie H , Demireva EY , Chen C (2017) PNLDC1 is essential for piRNA 3′ end trimming and transposon silencing during spermatogenesis in mice. Nat Commun 8: 2–11 29018194 10.1038/s41467-017-00854-4 PMC5635004

[embr202153801-bib-0019] Doetsch F , Caille I , Lim DA , Garcia‐Verdugo JM , Alvarez‐Buylla A (1999) Subventricular zone astrocytes are neural stem cells in the adult mammalian brain. Cell 97: 703–716 10380923 10.1016/s0092-8674(00)80783-7

[embr202153801-bib-0020] Eisen MB , Spellman PT , Brown PO , Botstein D (1999) Cluster analysis and display of genome‐wide expression patterns. Proc Natl Acad Sci USA 95: 12930–12933 10.1073/pnas.95.25.14863 PMC24541 9843981

[embr202153801-bib-0021] Encinas JM , Michurina TV , Peunova N , Park JH , Tordo J , Peterson DA , Fishell G , Koulakov A , Enikolopov G (2011) Division‐coupled astrocytic differentiation and age‐related depletion of neural stem cells in the adult hippocampus. Cell Stem Cell 8: 566–579 21549330 10.1016/j.stem.2011.03.010 PMC3286186

[embr202153801-bib-0022] Escartin C , Galea E , Lakatos A , O'Callaghan JP , Petzold GC , Serrano‐Pozo A , Steinhäuser C , Volterra A , Carmignoto G , Agarwal A *et al* (2021) Reactive astrocyte nomenclature, definitions, and future directions. Nat Neurosci 24: 312–325 33589835 10.1038/s41593-020-00783-4 PMC8007081

[embr202153801-bib-0023] Gainetdinov I , Colpan C , Arif A , Cecchini K , Zamore PD (2018) A single mechanism of biogenesis, initiated and directed by PIWI proteins, explains piRNA production in most animals. Mol Cell 71: 775–790 30193099 10.1016/j.molcel.2018.08.007 PMC6130920

[embr202153801-bib-0024] Ghosheh Y , Seridi L , Ryu T , Takahashi H , Orlando V , Carninci P , Ravasi T (2016) Characterization of piRNAs across postnatal development in mouse brain. Sci Rep 6: 1–7 27112104 10.1038/srep25039 PMC4844963

[embr202153801-bib-0025] Girard A , Sachidanandam R , Hannon GJ , Carmell MA (2006) A germline‐specific class of small RNAs binds mammalian Piwi proteins. Nature 442: 199–202 16751776 10.1038/nature04917

[embr202153801-bib-0026] Huang DW , Sherman BT , Lempicki RA (2009) Systematic and integrative analysis of large gene lists using DAVID bioinformatics resources. Nat Protoc 4: 44–57 19131956 10.1038/nprot.2008.211

[embr202153801-bib-0027] Jain G , Stuendl A , Rao P , Berulava T , Pena Centeno T , Kaurani L , Burkhardt S , Delalle I , Kornhuber J , Hüll M *et al* (2019) A combined miRNA–piRNA signature to detect Alzheimer's disease. Transl Psychiatry 9: 250 31591382 10.1038/s41398-019-0579-2 PMC6779890

[embr202153801-bib-0028] Jin WN , Shi K , He W , Sun JH , Van Kaer L , Shi FD , Liu Q (2021) Neuroblast senescence in the aged brain augments natural killer cell cytotoxicity leading to impaired neurogenesis and cognition. Nat Neurosci 24: 61–73 33257875 10.1038/s41593-020-00745-w

[embr202153801-bib-0029] Keam SP , Young PE , McCorkindale AL , Dang THY , Clancy JL , Humphreys DT , Preiss T , Hutvagner G , Martin DIK , Cropley JE *et al* (2014) The human Piwi protein Hiwi2 associates with tRNA‐derived piRNAs in somatic cells. Nucleic Acids Res 42: 8984–8995 25038252 10.1093/nar/gku620 PMC4132735

[embr202153801-bib-0030] Kirino Y , Mourelatos Z (2007) Mouse Piwi‐interacting RNAs are 2′‐O‐methylated at their 3′ termini. Nat Struct Mol Biol 14: 347–348 17384647 10.1038/nsmb1218

[embr202153801-bib-0031] Krishan A (1975) Rapid flow cytofluorometric analysis of mammalian cell cycle by propidium iodide staining. J Cell Biol 66: 188–193 49354 10.1083/jcb.66.1.188 PMC2109516

[embr202153801-bib-0032] Langmead B , Salzberg SL (2012) Fast gapped‐read alignment with Bowtie 2. Nat Methods 9: 357–359 22388286 10.1038/nmeth.1923 PMC3322381

[embr202153801-bib-0033] Lee JH , Schütte D , Wulf G , Füzesi L , Radzun HJ , Schweyer S , Engel W , Nayernia K (2006) Stem‐cell protein Piwil2 is widely expressed in tumors and inhibits apoptosis through activation of Stat3/Bcl‐XL pathway. Hum Mol Genet 15: 201–211 16377660 10.1093/hmg/ddi430

[embr202153801-bib-0034] Lee EJ , Banerjee S , Zhou H , Jammalamadaka A , Arcila M , Manjunath BS , Kosik KS (2011) Identification of piRNAs in the central nervous system. RNA 17: 1090–1099 21515829 10.1261/rna.2565011 PMC3096041

[embr202153801-bib-0035] Leighton LJ , Wei W , Marshall PR , Ratnu VS , Li X , Zajaczkowski EL , Spadaro PA , Khandelwal N , Kumar A , Bredy TW (2019) Disrupting the hippocampal Piwi pathway enhances contextual fear memory in mice. Neurobiol Learn Mem 161: 202–209 30965112 10.1016/j.nlm.2019.04.002

[embr202153801-bib-0036] Liddelow SA , Guttenplan KA , Clarke LE , Bennett FC , Bohlen CJ , Schirmer L , Bennett ML , Münch AE , Chung W‐S , Peterson TC *et al* (2017) Neurotoxic reactive astrocytes are induced by activated microglia. Nature 541: 481–487 28099414 10.1038/nature21029 PMC5404890

[embr202153801-bib-0037] Liu GY , Sabatini DM (2020) mTOR at the nexus of nutrition, growth, ageing and disease. Nat Rev Mol Cell Biol 21: 183–203 31937935 10.1038/s41580-019-0199-y PMC7102936

[embr202153801-bib-0038] Love MI , Huber W , Anders S (2014) Moderated estimation of fold change and dispersion for RNA‐seq data with DESeq2. Genome Biol 15: 1–21 10.1186/s13059-014-0550-8 PMC4302049 25516281

[embr202153801-bib-0039] Madisen L , Zwingman TA , Sunkin SM , Oh SW , Zariwala HA , Gu H , Ng LL , Palmiter RD , Hawrylycz MJ , Jones AR *et al* (2010) A robust and high‐throughput Cre reporting and characterization system for the whole mouse brain. Nat Neurosci 13: 133–140 20023653 10.1038/nn.2467 PMC2840225

[embr202153801-bib-0040] Martín‐Suárez S , Valero J , Muro‐García T , Encinas JM (2019) Phenotypical and functional heterogeneity of neural stem cells in the aged hippocampus. Aging Cell 18: 1–14 10.1111/acel.12958 PMC6612636 30989815

[embr202153801-bib-0041] Mignone JL , Kukekov V , Chiang AS , Steindler D , Enikolopov G (2004) Neural stem and progenitor cells in nestin‐GFP transgenic mice. J Comp Neurol 469: 311–324 14730584 10.1002/cne.10964

[embr202153801-bib-0042] Mills EW , Green R (2017) Ribosomopathies: there's strength in numbers. Sciencе 358: 1–8 10.1126/science.aan2755 29097519

[embr202153801-bib-0043] Muotri AR , Chu VT , Marchetto MCN , Deng W , Moran JV , Gage FH (2005) Somatic mosaicism in neuronal precursor cells mediated by L1 retrotransposition. Nature 435: 903–910 15959507 10.1038/nature03663

[embr202153801-bib-0044] Nandi S , Chandramohan D , Fioriti L , Melnick AM , Hébert JM , Mason CE , Rajasethupathy P , Kandel ER (2016) Roles for small noncoding RNAs in silencing of retrotransposons in the mammalian brain. Proc Natl Acad Sci USA 113: 12697–12707 27791114 10.1073/pnas.1609287113 PMC5111663

[embr202153801-bib-0045] Nolde MJ , Cheng EC , Guo S , Lin H (2013) Piwi genes are dispensable for normal hematopoiesis in mice. PLoS One 8: 1–8 10.1371/journal.pone.0071950 PMC3751959 24058407

[embr202153801-bib-0046] Ozata DM , Gainetdinov I , Zoch A , O'Carroll D , Zamore PD (2019) PIWI‐interacting RNAs: small RNAs with big functions. Nat Rev Genet 20: 89–108 30446728 10.1038/s41576-018-0073-3

[embr202153801-bib-0047] Page NF , Gandal MJ , Estes ML , Cameron S , Buth J , Parhami S , Ramaswami G , Murray K , Amaral DG , Van de Water JA *et al* (2021) Alterations in retrotransposition, synaptic connectivity, and myelination implicated by transcriptomic changes following maternal immune activation in nonhuman primates. Biol Psychiatry 89: 896–910 33386132 10.1016/j.biopsych.2020.10.016 PMC8052273

[embr202153801-bib-0048] Patro R , Duggal G , Love MI , Irizarry RA , Kingsford C (2017) Salmon provides fast and bias‐aware quantification of transcript expression. Nat Methods 14: 417–419 28263959 10.1038/nmeth.4197 PMC5600148

[embr202153801-bib-0049] Penning A , Tosoni G , Abiega O , Bielefeld P , Gasperini C , De Pietri Tonelli D , Fitzsimons CP , Salta E (2022) Adult neural stem cell regulation by small non‐coding RNAs: physiological significance and pathological implications. Front Cell Neurosci 15: 1–20 10.3389/fncel.2021.781434 PMC8764185 35058752

[embr202153801-bib-0050] Perera BPU , Tsai ZTY , Colwell ML , Jones TR , Goodrich JM , Wang K , Sartor MA , Faulk C , Dolinoy DC (2019) Somatic expression of piRNA and associated machinery in the mouse identifies short, tissue‐specific piRNA. Epigenetics 14: 504–521 30955436 10.1080/15592294.2019.1600389 PMC6557559

[embr202153801-bib-0051] Pons‐Espinal M , de Luca E , Marzi MJ , Beckervordersandforth R , Armirotti A , Nicassio F , Fabel K , Kempermann G , De Pietri Tonelli D (2017) Synergic functions of miRNAs determine neuronal fate of adult neural stem cells. Stem Cell Reports 8: 1046–1061 28330621 10.1016/j.stemcr.2017.02.012 PMC5390108

[embr202153801-bib-0052] Pons‐Espinal M , Gasperini C , Marzi MJ , Braccia C , Armirotti A , Pötzsch A , Walker TL , Fabel K , Nicassio F , Kempermann G *et al* (2019) MiR‐135a‐5p is critical for exercise‐induced adult neurogenesis. Stem Cell Reports 12: 1298–1312 31130358 10.1016/j.stemcr.2019.04.020 PMC6565832

[embr202153801-bib-0053] Quinlan AR , Hall IM (2010) BEDTools: a flexible suite of utilities for comparing genomic features. Bioinformatics 26: 841–842 20110278 10.1093/bioinformatics/btq033 PMC2832824

[embr202153801-bib-0054] Robinson MD , Oshlack A (2010) A scaling normalization method for differential expression analysis of RNA‐seq data. Genome Biol 11: R25 20196867 10.1186/gb-2010-11-3-r25 PMC2864565

[embr202153801-bib-0055] Rojas‐Rıós P , Simonelig M (2018) piRNAs and PIWI proteins: regulators of gene expression in development and stem cells. Development 145: 1–13 10.1242/dev.161786 30194260

[embr202153801-bib-0056] Schindelin J , Arganda‐Carreras I , Frise E , Kaynig V , Longair M , Pietzsch T , Preibisch S , Rueden C , Saalfeld S , Schmid B *et al* (2012) Fiji: an open‐source platform for biological‐image analysis. Nat Methods 9: 676–682 22743772 10.1038/nmeth.2019 PMC3855844

[embr202153801-bib-0057] Schouten M , Bielefeld P , Garcia‐Corzo L , Passchier EMJ , Gradari S , Jungenitz T , Pons‐Espinal M , Gebara E , Martín‐Suárez S , Lucassen PJ *et al* (2020) Circadian glucocorticoid oscillations preserve a population of adult hippocampal neural stem cells in the aging brain. Mol Psychiatry 25: 1382–1405 31222184 10.1038/s41380-019-0440-2 PMC7303016

[embr202153801-bib-0058] Sharma AK , Nelson MC , Brandt JE , Wessman M , Mahmud N , Weller KP , Hoffman R (2001) Human CD34^+^ stem cells express the hiwi gene, a human homologue of the Drosophila gene Piwi. Blood 97: 426–434 11154219 10.1182/blood.v97.2.426

[embr202153801-bib-0059] Sierra A , Martín‐Suárez S , Valcárcel‐Martín R , Pascual‐Brazo J , Aelvoet SA , Abiega O , Deudero JJ , Brewster AL , Bernales I , Anderson AE *et al* (2015) Neuronal hyperactivity accelerates depletion of neural stem cells and impairs hippocampal neurogenesis. Cell Stem Cell 16: 488–503 25957904 10.1016/j.stem.2015.04.003 PMC4443499

[embr202153801-bib-0060] Toda T , Parylak SL , Linker SB , Gage FH (2019) The role of adult hippocampal neurogenesis in brain health and disease. Mol Psychiatry 24: 67–87 29679070 10.1038/s41380-018-0036-2 PMC6195869

[embr202153801-bib-0061] Torres AG , Reina O , Attolini CSO , De Pouplana LR (2019) Differential expression of human tRNA genes drives the abundance of tRNA‐derived fragments. Proc Natl Acad Sci USA 116: 8451–8456 30962382 10.1073/pnas.1821120116 PMC6486751

[embr202153801-bib-0062] Upton KR , Gerhardt DJ , Jesuadian JS , Richardson SR , Sánchez‐Luque FJ , Bodea GO , Ewing AD , Salvador‐Palomeque C , Van Der Knaap MS , Brennan PM *et al* (2015) Ubiquitous L1 mosaicism in hippocampal neurons. Cell 161: 228–239 25860606 10.1016/j.cell.2015.03.026 PMC4398972

[embr202153801-bib-0063] Vicidomini G , Bianchini P , Diaspro A (2018) STED super‐resolved microscopy. Nat Methods 15: 173–182 29377014 10.1038/nmeth.4593

[embr202153801-bib-0064] Viero G , Lunelli L , Passerini A , Bianchini P , Gilbert RJ , Bernabò P , Tebaldi T , Diaspro A , Pederzolli C , Quattrone A (2015) Three distinct ribosome assemblies modulated by translation are the building blocks of polysomes. J Cell Biol 208: 581–596 25713412 10.1083/jcb.201406040 PMC4347638

[embr202153801-bib-0065] Wakisaka KT (2019) The dawn of pirna research in various neuronal disorders. Front Biosci 24: 1440–1451 10.2741/4789 31136989

[embr202153801-bib-0066] Walker TL , Kempermann G (2014) One mouse, two cultures: isolation and culture of adult neural stem cells from the two neurogenic zones of individual mice. J Vis Exp 84: 1–9 10.3791/51225 PMC4131911 24637893

[embr202153801-bib-0067] Yu C‐W (1992) The assessment of cellular proliferation by immunohistochemistry: a review of currently available methods and their applications. Histochem J 24: 121–131 1349881 10.1007/BF01047461

[embr202153801-bib-0068] Zhang P , Si X , Skogerbø G , Wang J , Cui D , Li Y , Sun X , Liu L , Sun B , Chen R *et al* (2014) piRBase: a web resource assisting piRNA functional study. Database 2014: 1–7 10.1093/database/bau110 PMC4243270 25425034

[embr202153801-bib-0069] Zhao P , Yao M , Chang S , Gou L , Liu M , Qiu Z (2015) Novel function of PIWIL1 in neuronal polarization and migration via regulation of microtubule‐associated proteins. Mol Brain 8: 1–12 26104391 10.1186/s13041-015-0131-0 PMC4477296

