# Supplementary material for: Piwil2 (Mili) sustains neurogenesis and prevents cellular senescence in the postnatal hippocampus
Source: EMBO Rep. 2022 Dec 6;24(2):e53801. doi: 10.15252/embr.202153801 (PMC9900342; doi:10.15252/embr.202153801)
Supplement: Supplementary file 1 — Expanded View Figures PDF [file EMBR-24-e53801-s010.pdf]

Expanded View Figures

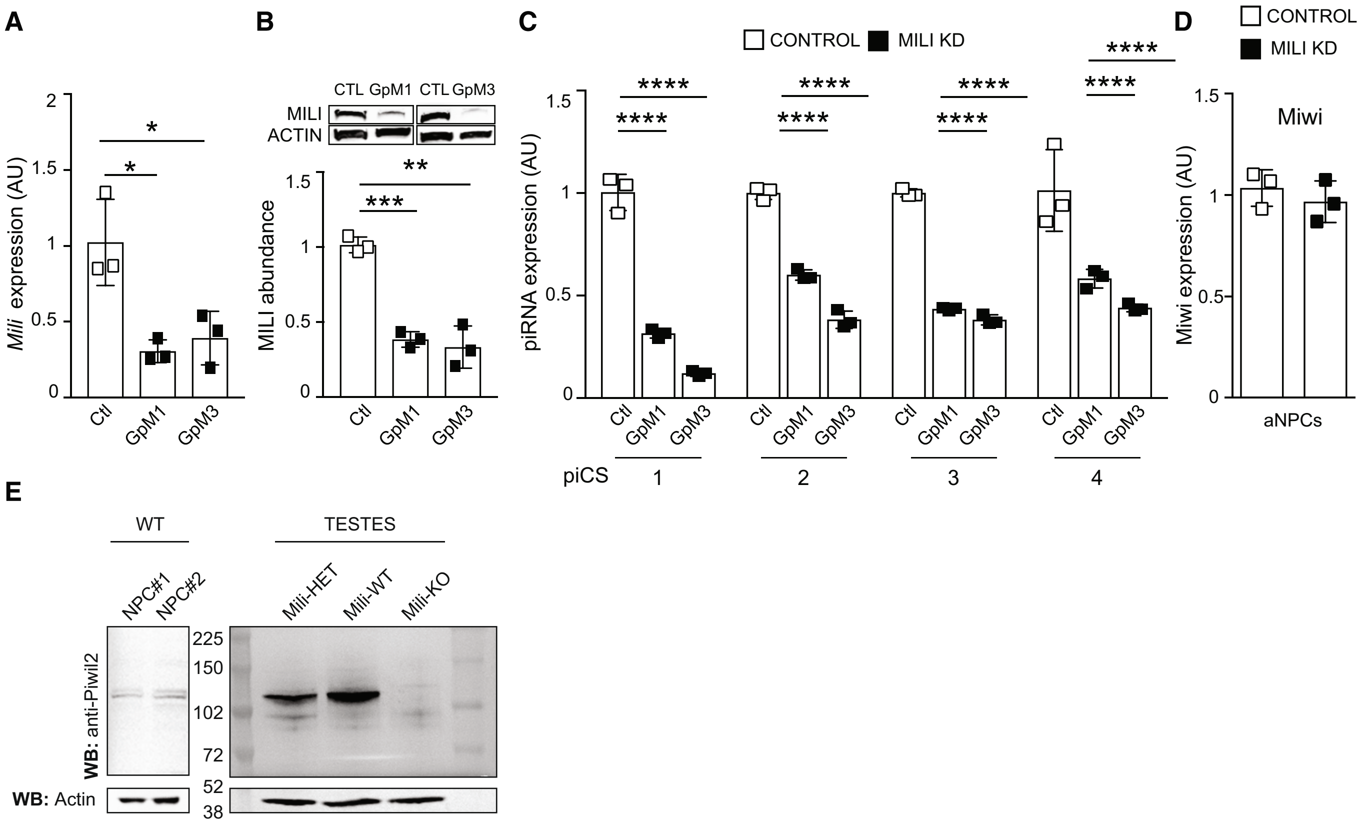

Figure EV1. Mili KD depletes piRNAs and does not affect Miwi. Relative to Fig 2.

A, B *Mili* mRNA expression (A); western blot (B, inset) and quantification of Mili protein abundance (B, bar graph) in lysates from aNPCs upon transfection with control GapmeR (Ctl) or two different GapmeRs (GpM1, GpM3) targeting *Mili*.

C Expression of transcripts of piRNA-cluster consensus sequences (piCS) in control and Mili KD (two independent GapmeRs) aNPCs.

D Relative expression of *Miwi* transcript in undifferentiated aNPCs transduced with viruses transcribing a Scrambled short hairpin (Control) and short hairpin against *Mili* (Mili KD).

E Western blot of Mili protein in lysates from aNPCs ($n = 2$ biological replicates) and from testes of mice wildtype (WT), heterozygous (HET) or knockout (KO) for the *Mili* gene.

Data information: data are expressed as mean ± SEM, $n = 3$ biological replicates (A–C). $*P < 0.05$, $**P < 0.01$, $***P < 0.001$, $****P < 0.0001$, as assessed by the two-tailed Student's *t*-test (A, B) or One-way ANOVA, *post-hoc* Bonferroni (C).
Source data are available online for this figure.

© 2022 The Authors

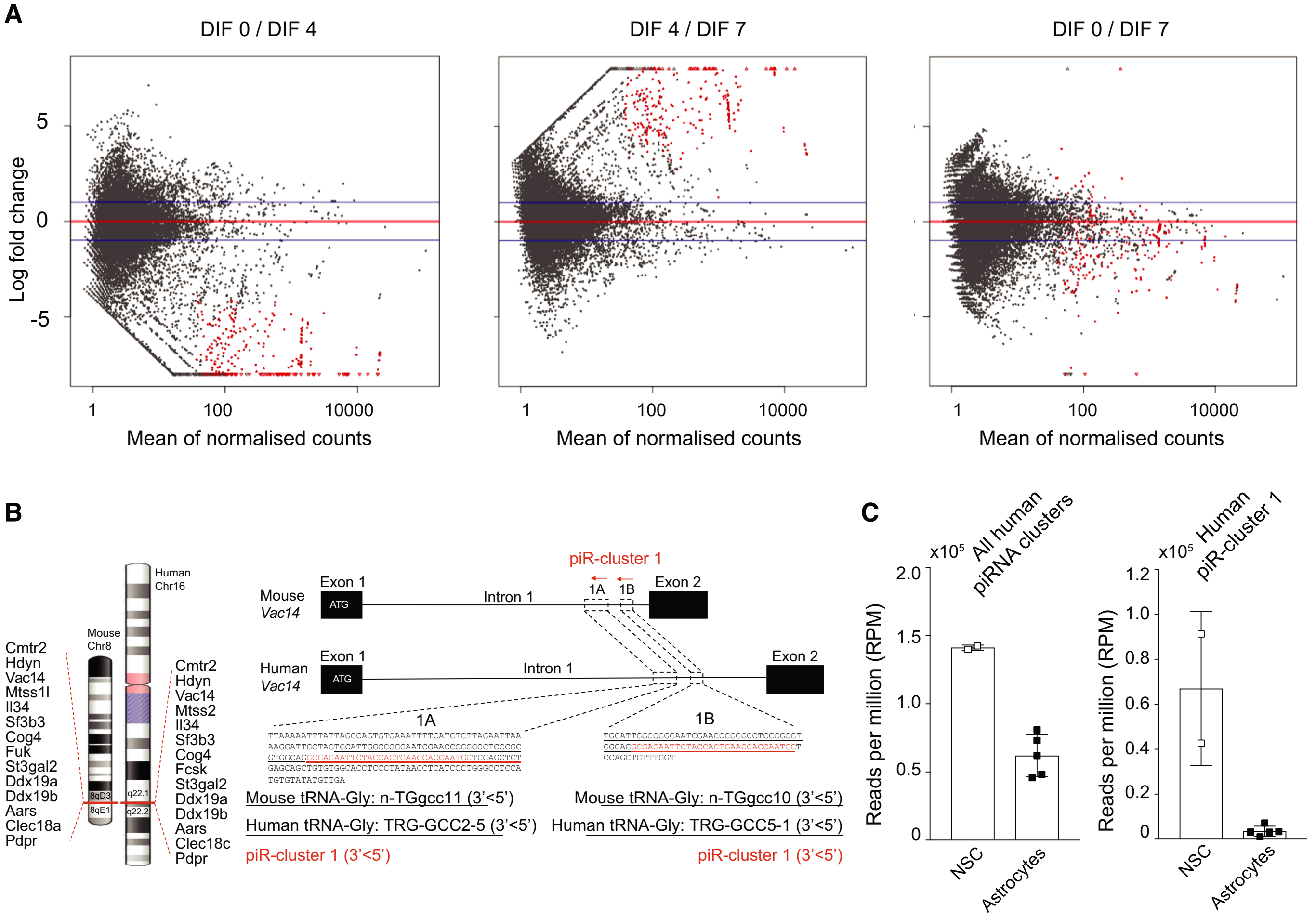

Figure EV2. Expression of piRNAs in mouse and human NSC and progeny. Relative to Fig 3.

A Pairwise comparison of 298 piRNA clusters differentially expressed in undifferentiated aNPCs (DIF0) or neuroblasts upon viral-induced neurogenesis (DIF4-7). *n* = 2 biological replicates.

B Chromosomal location of piR-cluster 1 in mouse and human; (Right) genomic location and sequences (underlined red text) of piR-cluster 1 corresponding to tRNAGly genes (underlined black text).

C Expression of piRNA clusters (left) and piR-cluster 1 (right) in human NSC and astrocytes. *n* = 2 biological replicates of human NSCs; *n* = 5 biological replicates of human astrocytes.

Data information: data in C are expressed as mean ± SEM.

 © 2022 The Authors

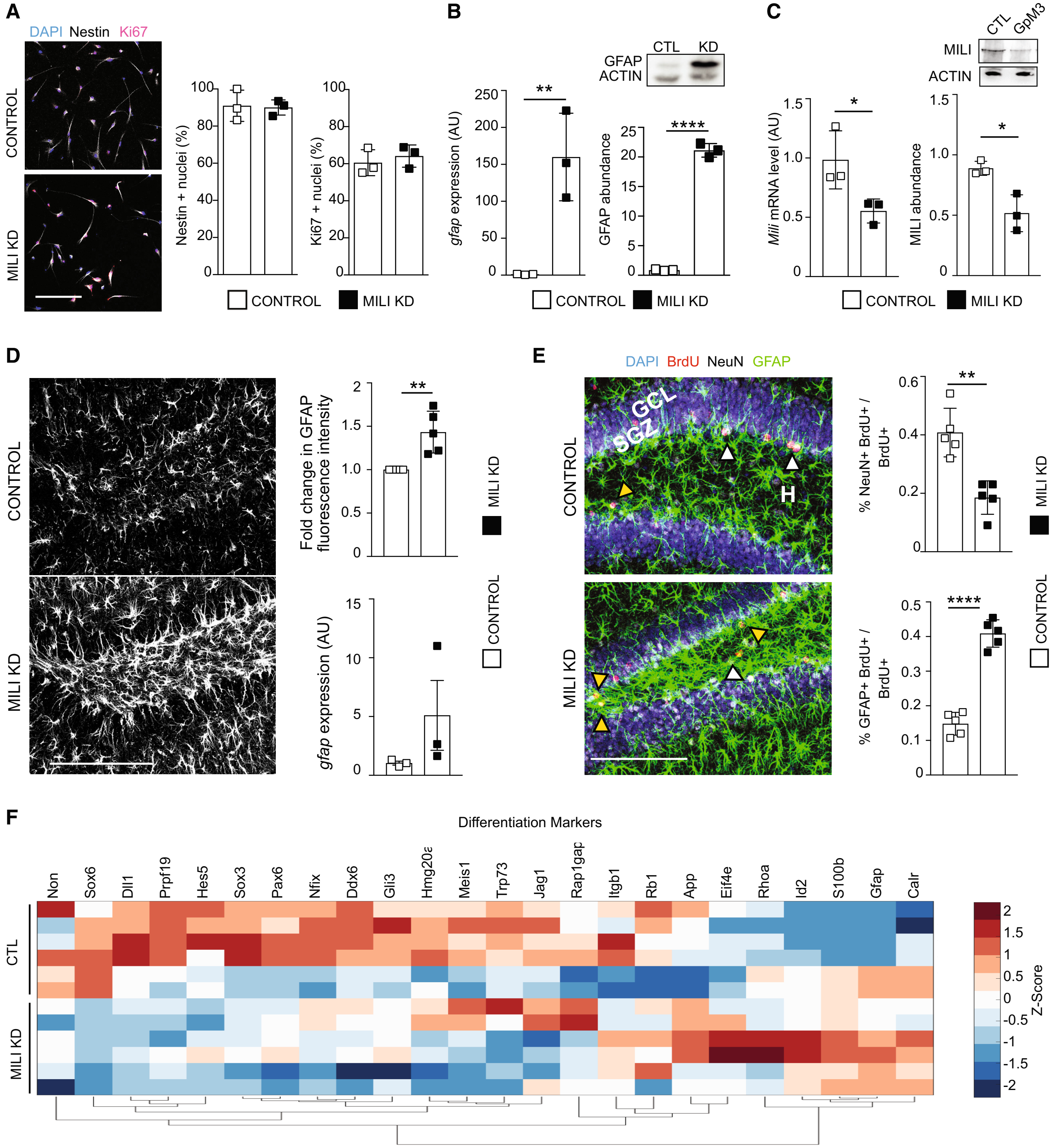
A
DAPI Nestin Ki67
CONTROL
MILI KD
Nestin + nuclei (%)
Ki67 + nuclei (%)
CONTROL MILI KD
B
CTL KD
GFAP
ACTIN
gfap expression (AU)
GFAP abundance
CONTROL MILI KD
C
CTL GpM3
MILI
ACTIN
Mili mRNA level (AU)
MILI abundance
CONTROL MILI KD
D
CONTROL
MILI KD
Fold change in GFAP fluorescence intensity
gfap expression (AU)
E
DAPI BrdU NeuN GFAP
GCL
SGZ
H
% NeuN+ BrdU+ / BrdU+
% GFAP+ BrdU+ / BrdU+
F
Differentiation Markers
Non Sox6 Dll1 Prpf19 Hes5 Sox3 Pax6 Nfix Ddx6 Gli3 Hmg20a Meis1 Trp73 Jag1 Rap1gap Itgb1 Rb1 App Eif4e Rhoa Id2 S100b Gfap Calr
CTL
MILI KD
Z-Score

Figure EV3.

Figure EV3. Depletion of Mili and piRNAs does not alter stemness and proliferation and induces the expression of genes involved in astrogliogenesis. Related to Fig 4.

A (left) Confocal microscopy images of undifferentiated aNPCs transduced *in vitro* with viruses transcribing a Scrambled (Control) and shMILI (Mili KD), immunostained with anti-Nestin (white), or anti-Ki67 (purple) antibodies and stained for nuclear DNA with DAPI (blue); (right) Percentage of Nestin or $Ki67^{+}$ cells. over total cells.

B *Gfap* mRNA expression (left graph), western blot (inset), and quantification of Gfap protein abundance (right graph) in lysates from control and Mili KD neuroblasts at DIF7.

C *Mili* mRNA expression (left graph), western blot (inset), and quantification of Mili protein abundance (right graph) in lysates from mouse hippocampi 48 h after the injection of scrambled (Control) or GapmeR3 against *Mili* (Mili KD).

D Representative immunofluorescence micrograph of postnatal hippocampal sections immunostained for GFAP; (top right) quantification of GFAP fluorescence intensity level (top) in hippocampal section 30 dpi of GapmeR3 (Mili KD) compared with scrambled (Control). Expression of *Gfap* mRNA (bottom right) in the DG from mouse hippocampi 48 h after the injection of scrambled (Control) or GapmeR3 (Mili KD).

E (left) Representative immunofluorescence micrograph of postnatal hippocampal sections immunostained for GFAP (green), BrdU (red), NeuN (white), and nuclear DNA (blue) at 30 dpi of scrambled (Control) or GapmeR3 against Mili (Mili KD); (right) percentages of $NeuN^{+}BrdU^{+}$ (white arrowheads), or $GFAP^{+}BrdU^{+}$ (yellow arrowheads) double-positive cells over total $BrdU^{+}$ cells.

F RNA seq. expression data of genes encoding for proteins involved in astrogliogenesis and regulation of neuronal fate in Mili KD neuroblasts at DIF7, compared with Scrambled control. Expression heatmap correlation plots were computed by the k-means clustering method. Scale bar indicates *Z*-scores.

Data information: data are expressed as mean ± SEM, $n = 3$ (A–C, F) and $n = 5$ (D, E) biological replicates (in F each biological replicate was sequenced with two separate flow cells). *$P < 0.05$, **$P < 0.01$, ****$P < 0.0001$, as assessed by the two-tailed Student's *t*-test. GCL, granular cell layer, SGZ, subgranular zone. The scale bars represent 50 μm (A) and 100 μm (D, E).
Source data are available online for this figure.

 © 2022 The Authors

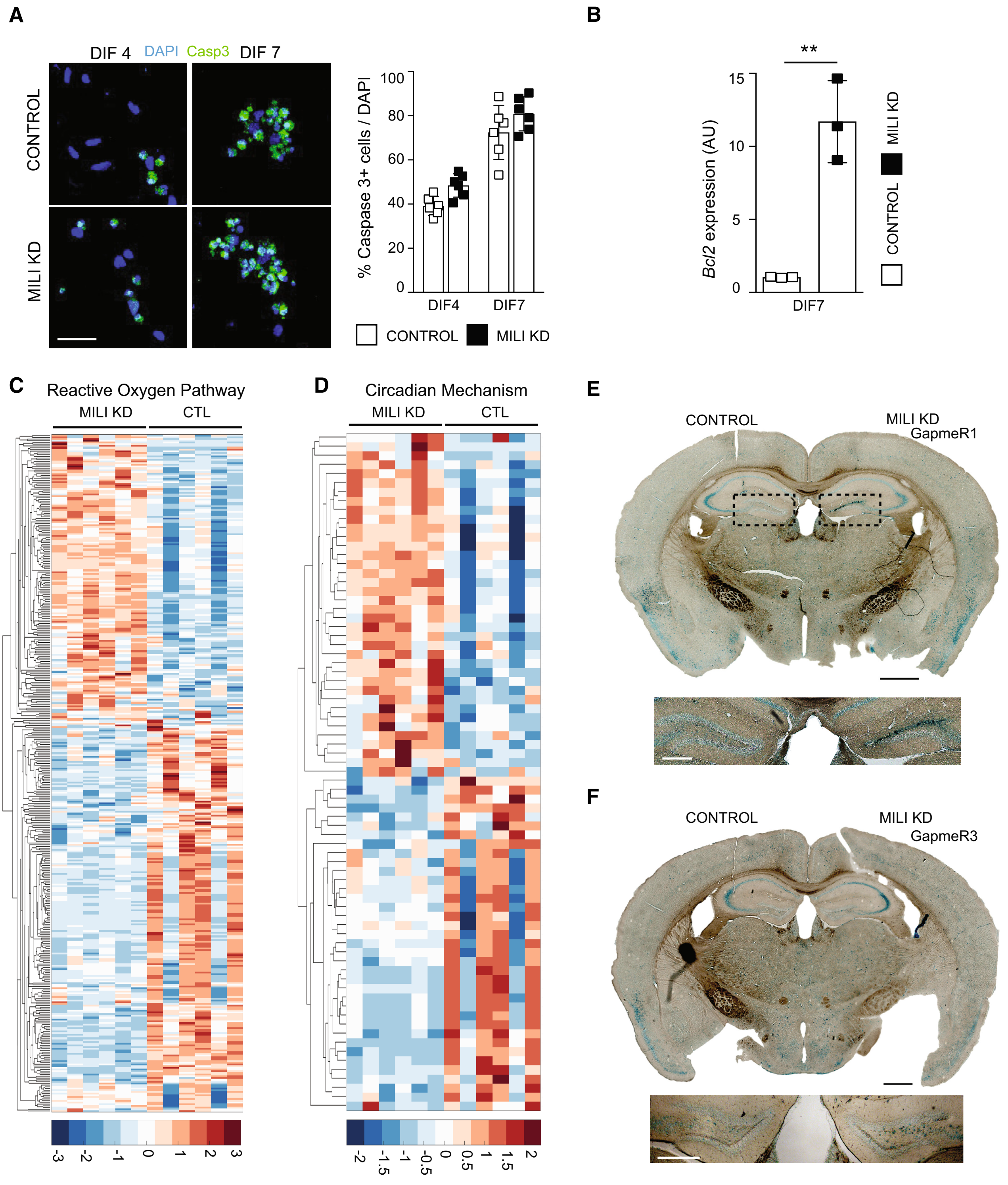
A
DIF 4
DAPI
Casp3
DIF 7
CONTROL
MILI KD
% Caspase 3+ cells / DAPI
100
80
60
40
20
0
DIF4
DIF7
CONTROL
MILI KD
B
**
15
10
5
0
Bcl2 expression (AU)
DIF7
CONTROL
MILI KD
C
Reactive Oxygen Pathway
MILI KD
CTL
-3
-2
-1
0
1
2
3
D
Circadian Mechanism
MILI KD
CTL
-2
-1.5
-1
-0.5
0
0.5
1
1.5
2
E
CONTROL
MILI KD
GapmeR1
F
CONTROL
MILI KD
GapmeR3

Figure EV4.

Figure EV4. Depletion of Mili and piRNAs does not lead to apoptosis and alters the expression of genes involved in inflammatory responses. Related to Fig 5.

A Representative fluorescence micrographs of control or Mili KD neuroblasts 4 or 7 days after spontaneous differentiation (DIF4, 7), immunostained with anti-cleaved caspase-3 (green) and for nuclear DNA with DAPI (blue). (Right) Percentage of cleaved Caspase-3^+ cells over total cells.

B *Bcl2* mRNA expression level in control or Mili KD neuroblasts at DIF7.

C, D Heatmap of differentially expressed transcripts in RNA seq from Mili KD neuroblasts, encoding proteins involved in ROS production (C) or circadian regulation (D). Target genes are listed in Dataset EV4. Expression heatmap correlation plots were computed by the k-means clustering method. Scale bar indicates *Z*-scores.

E, F Representative light-microscopy images of the ß-galactosidase staining in postnatal hippocampal sections, 30 dpi of scrambled (Control, left hemisphere) and GapmeR1 (E) or GapmeR3 (F) against *Mili* (Mili KD, right hemispheres). Dashed box in E indicates the areas shown in Fig 5. Bottom panels in (E, F) are higher magnification of the hippocampi shown in top panels.

Data information: data are expressed as mean ± SEM, $n = 6$ (A) and $n = 3$ (B–D) biological replicates (in C and D each biological replicate was sequenced with two separate flow cells). $^{**}P < 0.01$, as assessed by the two-tailed Student's *t*-test. The scale bars represent 50 µm (A), 1 mm (E, F top) and 500 µm (E, F bottom).

© 2022 The Authors

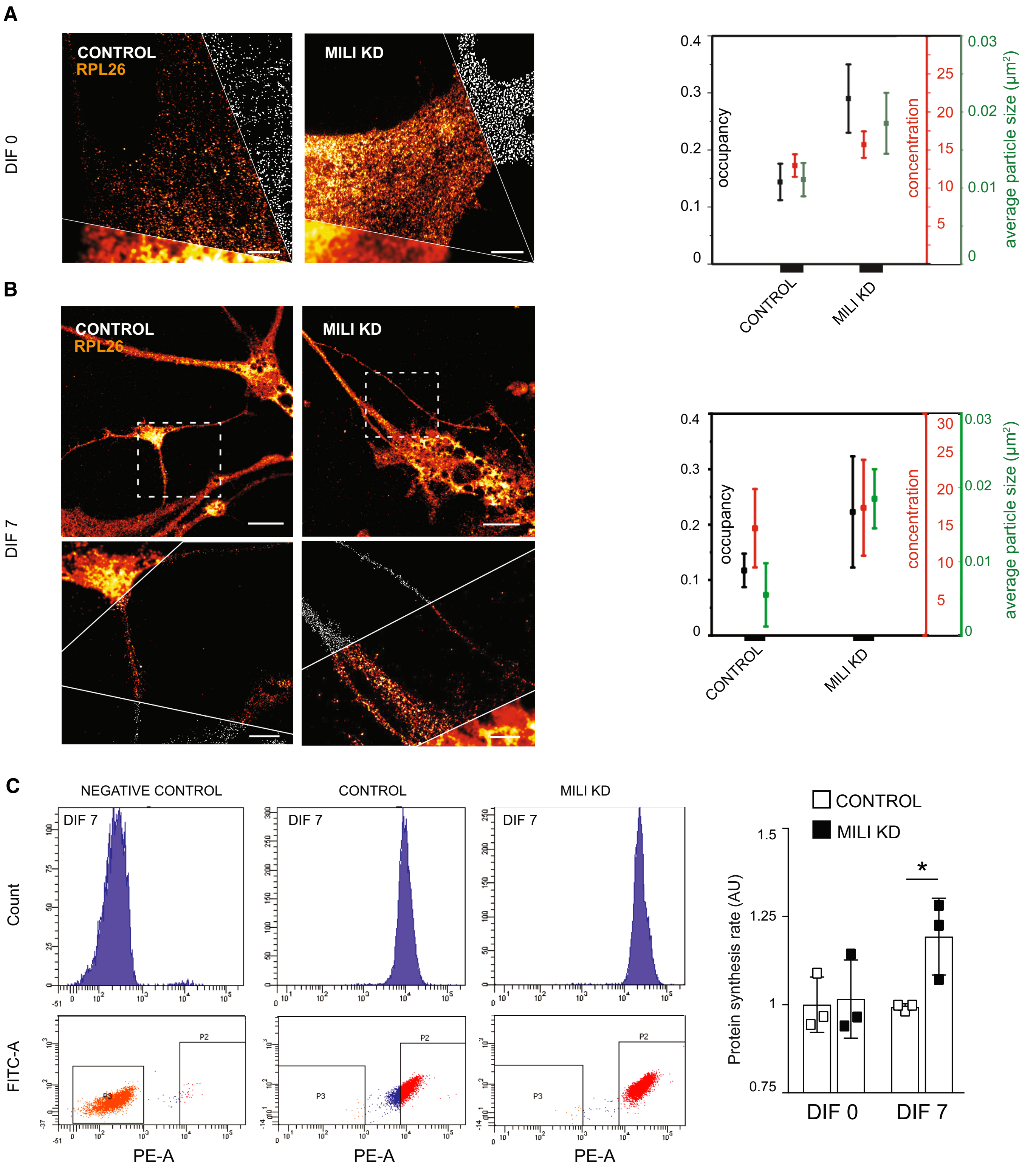
A
DIF 0
CONTROL
RPL26
MILI KD
occupancy
concentration
average particle size (μm²)
CONTROL
MILI KD
B
DIF 7
CONTROL
RPL26
MILI KD
occupancy
concentration
average particle size (μm²)
CONTROL
MILI KD
C
NEGATIVE CONTROL
CONTROL
MILI KD
DIF 7
Count
FITC-A
PE-A
P2
P3
CONTROL
MILI KD
Protein synthesis rate (AU)
DIF 0
DIF 7
*

Figure EV5.

Figure EV5. Depletion of Mili and piRNAs enhances polysome assembly and results in higher protein synthesis upon differentiation.

A, B Representative micrographs (Middle cut: g-STED nanoscopy; Bottom: Confocal; Top: analysis) of control and Mili KD undifferentiated aNPCs (DIF0) and neuroblasts (DIF7) immunostained for the ribosomal protein RPL26. (Right) Normalized distributions of the occupancy, concentration and average particle size of each polyribosome particle in the indicated cells.

C Protein synthesis rate (right) as determined by OPP incorporation assay with flow cytometry (left) in control and Mili KD undifferentiated aNPCs (DIF0) and neuroblasts (DIF7).

Data information: data are expressed as mean ± SEM, $n = 3$ biological replicates. $^*P < 0.05$, as assessed by the two-tailed Student's t-test. The scale bars represent 2 μm (A) and 10 μm (B).

 © 2022 The Authors
