# Supplementary material for: Piwil2 (Mili) sustains neurogenesis and prevents cellular senescence in the postnatal hippocampus
Source: EMBO Rep. 2022 Dec 6;24(2):e53801. doi: 10.15252/embr.202153801 (PMC9900342; doi:10.15252/embr.202153801)
Supplement: Supplementary file 2 — Table EV1 [file EMBR-24-e53801-s001.pdf]

Table EV1
Oligonucleotides: primers, TaqMan probes, and GapmeRs used in this publication.

Primers used for real-time qPCR		
Gene Name	Forward primer (5' to 3')	Reverse primer (5' to 3')
Actin	GGCTGTATTCCCCTCCATCG	CCAGTTGGTAACAATGCCATGT
Mili	GGCCAGCATAAATCTCACAC	TAGCTGGCCATCAGACACTC
Miwi	TAATTGGCCTGGAGTCATCC	GAGGTAGTAGAGGCGGTTGG
Gfap	GGGGCAAAAGCACCAAAGAAG	GGGACAACTTGTATTGTGAGCC
Complement C3	CCAGCTCCCCATTACGTCTG	GCACTTGCCTCTTTAGGAAGTC
Serpina 3n	ATTTGTCCCAATGTCTGCGAA	TGGCTATCTTGGCTATAAAGGGG
Cxcl10	CCAAGTGCTGCCGTCATTTTC	GGCTGGCAGGGATGATTTCAA
Vac14	AAGTGGCTCTACCATCTCTACAT	ACAACCTCATCAGATTCGTCAGA
Lars2	CATAGAGAGGAATTTGCACCCTG	GCCAGTCCTGCTTCATAGAGTTT
Rpl13a	AGCCTACCAGAAAGTTTGCTTAC	GCTTCTTCTTCCGATAGTGCATC
Rpl17a	ATCAAGAGGGTCAAACCTTCGT	CCACCATTATACCGCCGGAA
Cyclin A	GCCTTCACCATTCATGTGGAT	TTGCTGCGGGTAAAGAGACAG
Cyclin D1	GCGTACCCTGACACCAATCTC	CTCCTCTTCGCACTTCTGCTC
Cyclin E	GATCCAGAAAAAGGAAGGCAAA	TGAAGAAATTGCCAAGATTGACA

Primers used for real-time qPCR (TaqMan)			
Target	Forward primer (5' to 3')	Reverse primer (5' to 3')	Probe (5' to 3')
L1 5'UTR Af	TGCCCACTGAAACTAAGGAGA	GCTTGTTCTTCAGGTGACTCTGT	FAM-TGCTACCCTCCAGGTCTGCT-MGBNFQ
L1 5'UTR Gf	CCAAACACCAGATAACTGTACACC	CGTGGGAGACAAGCTCTCTT	FAM-TGAAAGAGGAGAGCTTGCCT-MGBNFQ
L1 5'UTR Tf	TGAGCACTGAAACTCAGAGGAG	GATTGTTCTTCTGGTGATTCTGTTA	FAM-GAATCTGTCTCCCAGGTCTG-MGBNFQ
L1 ORF2	CCCTCAACAGAGGAATGGAT	CCATCCATTTGGCTAGGAAT	FAM-AAATGTGGTACATCTACACAATGGA-MGBNFQ
SINE B1	TGGCGCACGCCTTTAATC	GAGACAGGGTTTCTCTGTGTAGCC	FAM-CAGAGGCAGGCGGAT-MGBNFQ
5s rRNA	ACGGCCATACCACCCTGAA	GGTCTCCCATCCAAGTACTAACCA	FAM-CCGAGATCAGACGAGAT-MGBNFQ
Ubiquitin C	ACAGACGTACCTTCCTCACC	CCCCATCACACCCAAGAACA	VIC-AAAAAGAGCCCTCCTTGTGC-MGBNFQ

GapmeR sequence			
Catalog	Product name	Product Sequence 5'-3'	Target region on Mili transcript
339516 LG00000002-DFA	Negative Control	AACACGTCTATACGC	Non target
339512 LG00223301-DFA	GapmeR1	GAGTGCAGTGAAGTTG	Coding Sequence (CDS)
339512 LG00223303-DFA	GapmeR3	TCACTCTGTAATCTTG	3' UTR
