# Supplementary figures and images for: Piwil2 (Mili) sustains neurogenesis and prevents cellular senescence in the postnatal hippocampus
Source: EMBO Rep. 2022 Dec 6;24(2):e53801. doi: 10.15252/embr.202153801 (PMC9900342; doi:10.15252/embr.202153801)

Fig. EV1B

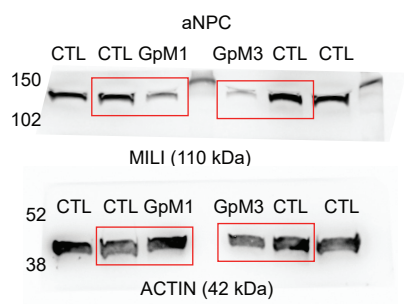

Supplement: Supplementary file 7 — Source Data for Expanded View [file EMBR-24-e53801-s008.zip › Source_Data_for_Figure_EV1B.pdf]

Fig. EV3B

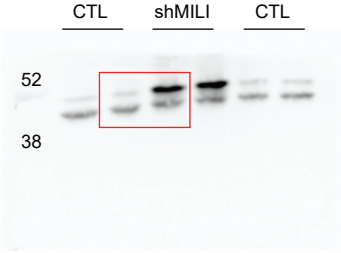

GFAP (49,88 kDa)  
ACTIN (42 kDa)

Fig. EV3C

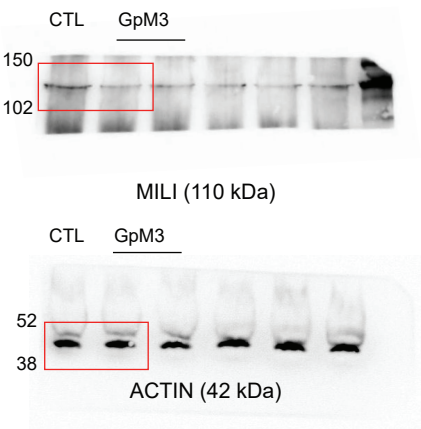

MILI (110 kDa)

ACTIN (42 kDa)

Supplement: Supplementary file 7 — Source Data for Expanded View [file EMBR-24-e53801-s008.zip › Source_Data_for_Figure_EV3.pdf]

Fig. 1B

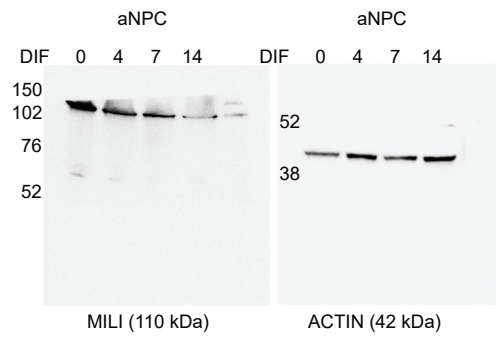

Fig. 1C

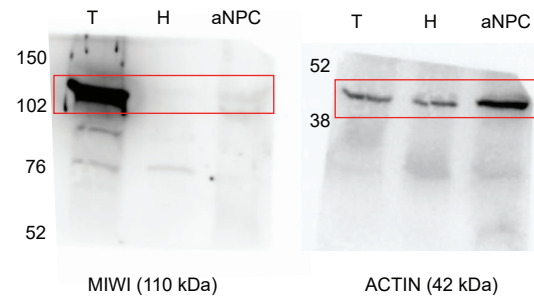

Fig. 1D

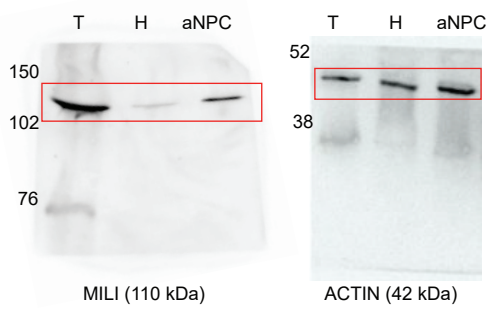

Fig. 1E

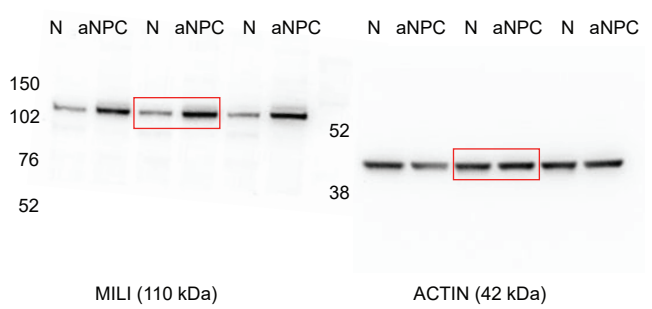

Supplement: Supplementary file 9 — Source Data for Figure 1 [file EMBR-24-e53801-s005.pdf]

Fig. 2E

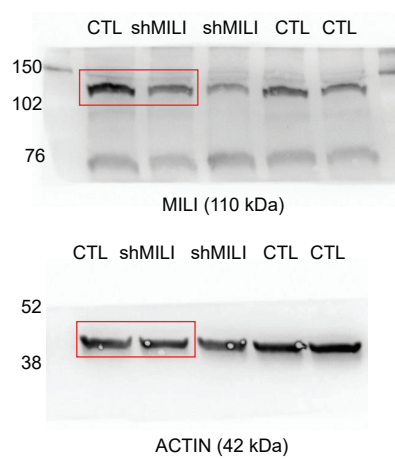

Supplement: Supplementary file 10 — Source Data for Figure 2 [file EMBR-24-e53801-s012.pdf]

Fig. 4C

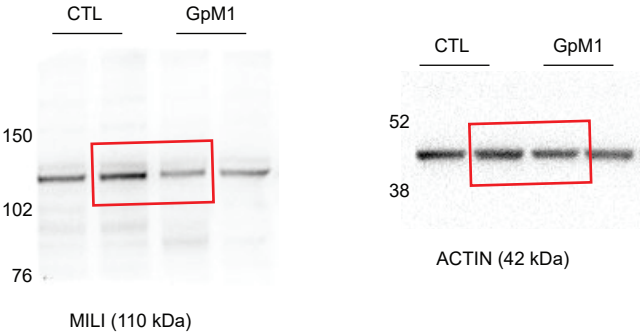

Supplement: Supplementary file 11 — Source Data for Figure 4 [file EMBR-24-e53801-s009.pdf]
