# Supplementary material for: Piwil2 (Mili) sustains neurogenesis and prevents cellular senescence in the postnatal hippocampus
Source: EMBO Rep. 2022 Dec 6;24(2):e53801. doi: 10.15252/embr.202153801 (PMC9900342; doi:10.15252/embr.202153801)
Supplement: Supplementary file 8 — PDF+ [file EMBR-24-e53801-s002.pdf]

Article

SOURCE DATA TRANSPARENT PROCESS OPEN ACCESS

EMBO reports

Piwil2 (Mili) sustains neurogenesis and prevents cellular senescence in the postnatal hippocampus

Caterina Gasperini[1], Kiril Tuntevski[1,2], Silvia Beatini[1], Roberta Pelizzoli[1], Amanda Lo Van[1], Damiano Mangoni[3], Rosa M Cossu[3], Giovanni Pascarella[4], Paolo Bianchini[5], Pascal Bielefeld[6], Margherita Scarpato[3], Meritxell Pons-Espinal[1], Remo Sanges[3,7], Alberto Diaspro[5], Carlos P Fitzsimons[6], Piero Carninci[4,8], Stefano Gustincich[3] & Davide De Pietri Tonelli[1,*]

Abstract

Adult neural progenitor cells (aNPCs) ensure lifelong neurogenesis in the mammalian hippocampus. Proper regulation of aNPC fate has thus important implications for brain plasticity and healthy aging. Piwi proteins and the small noncoding RNAs interacting with them (piRNAs) have been proposed to control memory and anxiety, but the mechanism remains elusive. Here, we show that Piwil2 (Mili) is essential for proper neurogenesis in the postnatal mouse hippocampus. RNA sequencing of aNPCs and their differentiated progeny reveal that Mili and piRNAs are dynamically expressed in neurogenesis. Depletion of Mili and piRNAs in the adult hippocampus impairs aNPC differentiation toward a neural fate, induces senescence, and generates reactive glia. Transcripts modulated upon Mili depletion bear sequences complementary or homologous to piRNAs and include repetitive elements and mRNAs encoding essential proteins for proper neurogenesis. Our results provide evidence of a critical role for Mili in maintaining fitness and proper fate of aNPCs, underpinning a possible involvement of the piRNA pathway in brain plasticity and successful aging.

Keywords hippocampal neurogenesis; Mili; Piwi-interacting RNAs; Piwil2; senescence
Subject Categories Neuroscience; RNA Biology
DOI 10.15252/embr.202153801 | Received 12 August 2021 | Revised 25 October 2022 | Accepted 10 November 2022 | Published online 6 December 2022
EMBO Reports (2023) 24: e53801

Introduction

A regulated balance of aNPC quiescence, proliferation, and differentiation guarantees lifelong neurogenesis in the adult hippocampus (Altman, 1962; Doetsch *et al*, 1999), prevents the generation of reactive glia (Encinas *et al*, 2011; Sierra *et al*, 2015; Clarke *et al*, 2018), and curbs neurodegeneration (Toda *et al*, 2019). Understanding the molecular control of aNPCs fate is pivotal to develop novel therapies aimed at preventing or delay age-dependent loss of neurogenesis and related pathological conditions.

The *Piwi* genes encode for an evolutionary conserved subfamily of Argonaute proteins that bind to Piwi-interacting RNAs (piRNAs), a class of single-stranded noncoding RNAs of 21–35 nucleotides. Piwi proteins and piRNAs (henceforth referred to as the piRNA pathway) are highly abundant in gonads, where they mainly target transposable elements (TEs) for degradation to maintain germline stem cell pools and male fertility (Czech *et al*, 2018; Ozata *et al*, 2019). Since its initial discovery, the piRNA pathway has been also implicated in regulating gene expression outside gonads, particularly in somatic stem cells (Rojas-Rıós & Simonelig, 2018). In fact, Piwi proteins are present in human hematopoietic stem cells, but their functions are dispensable for normal hematopoiesis in the mouse (Nolde *et al*, 2013), suggesting a possible role of the piRNA pathway in the control of self-renewal, rather than differentiation of these cells (Sharma *et al*, 2001).

Besides gonads, the highest piRNA expression in the adult mouse has been observed in the hippocampus (Perera *et al*, 2019), and proposed to control synaptic plasticity, memory, and anxiety (Lee *et al*, 2011; Zhao *et al*, 2015; Nandi *et al*, 2016; Leighton *et al*, 2019). piRNA abundance in neurons, however, is low compared to that of germline cells (Lee *et al*, 2011; Ghosheh *et al*, 2016; Nandi *et al*, 2016). Moreover, TE expression increases following the differentiation of NPC (Muotri *et al*, 2005), in parallel with the number of somatic TE insertions found in hippocampal neurons (Upton *et al*, 2015), arguing against functions of the piRNA pathway in postmitotic nerve cells. Given that the hippocampus is one of the niches in which neurogenesis persists beyond embryonic age, we

1 Neurobiology of miRNA Laboratory, Istituto Italiano di Tecnologia, Genoa, Italy
2 The Open University Affiliated Research Centre at Istituto Italiano di Tecnologia (ARC@IIT), Genoa, Italy
3 Central RNA Laboratory, Istituto Italiano di Tecnologia, Genoa, Italy
4 Division of Genomic Technologies, RIKEN Center for Life Science Technologies, Yokohama, Japan
5 Nanoscopy, CHT Erzelli, Istituto Italiano di Tecnologia, Genoa, Italy
6 Swammerdam Institute for Life Sciences, Faculty of Science, University of Amsterdam, Amsterdam, The Netherlands
7 Area of Neuroscience, SISSA, Trieste, Italy
8 Human Technopole, Milan, Italy
*Corresponding author. Tel: +39 010 2896 725; E-mail: davide.depietri@iit.it

© 2022 The Authors. Published under the terms of the CC BY 4.0 license.

hypothesize that the piRNA pathway may be present in aNPCs, possibly contributing to maintain the neurogenesis capacity lifelong (Penning *et al*, 2022).

Here, we studied the piRNA pathway in aNPCs of the postnatal mouse hippocampus *in vivo* and *in vitro*. By knockdown (KD) of Mili (i.e., one of the essential endoribonucleases for piRNA biogenesis and function) and Mili-dependent piRNAs, we investigated the consequences of their depletion for proliferation, survival, differentiation, and fate of aNPCs. With this approach, we aim to address functions of the piRNA pathway in adult neurogenesis, hence providing a system-level biological understanding of scientific and therapeutic value for brain plasticity and successful aging.

Results

Mili is preferentially expressed in aNPCs and depleted in neurogenesis

As an entry point to investigate the piRNA pathway in aNPCs we quantified the expression of *Piwil1* (Miwi) *Piwil2* (Mili) and *Piwil4* (Miwi2) transcripts, encoding the three main Piwi proteins present in the adult mouse (Czech *et al*, 2018; Ozata *et al*, 2019). Analysis of deep RNA sequencing (RNA seq) of total RNA from cultured aNPCs derived from neural stem cells (NSC) of the adult mouse Dentate Gyrus (DG) (Walker & Kempermann, 2014; Pons-Espinal *et al*, 2017) indicated that *Mili* is the most abundantly expressed of the three *Piwi* genes in neurogenesis (Fig 1A). In addition, other genes encoding for piRNA biogenesis cofactors were expressed in neurogenesis, including the transcription factors Zic2, Mybl1, and Meis1, the Tudor and KH domain-containing protein Tdrkh, and the helicase Mov10 (Dataset EV1). Interestingly, *Mili* expression showed a dynamic trend in neurogenesis. Indeed, its expression increased transiently from proliferating aNPCs (here referred to as days of differentiation (DIF0)) showing a peak at DIF4 upon onset of their spontaneous differentiation, whereas it decreased in differentiated progeny (DIF7-14) (Fig 1A). Similarly, the Mili protein abundance was higher in undifferentiated aNPCs (DIF0), or early upon onset of vector-induced neurogenesis (DIF4), compared with neuroblasts and neurons (DIF7 and 14, respectively) (Fig 1B). Next, we quantified the abundance of Miwi and Mili proteins in the mouse testis, whole hippocampus, and undifferentiated aNPCs (Fig 1C and D). As expected, the Miwi protein was very abundant in the testis but almost undetectable in the whole hippocampus or aNPCs (Fig 1C), whereas the Mili protein abundance in aNPCs was about 40% of the one in the testes (Fig 1D), and about four-fold higher than in primary hippocampal neurons (Fig 1E).

To validate this finding *in vivo*, we used the split-Cre viral approach to selectively label NSCs and their progeny in the postnatal hippocampus of Td-Tomato Cre-reporter mice (Pons-Espinal *et al*, 2017). Five days postviral injection (dpi), we found Mili protein in Td-Tomato positive (Td^+) NSCs of the subgranular zone (SGZ) of the DG (Fig 1F). To corroborate the immunofluorescence result, and to follow *Mili* expression during neurogenesis *in vivo*, we sorted Td^+ NSCs and their differentiated progeny at 10 and 30 dpi in the postnatal mouse hippocampus, respectively, and quantified *Mili* by real-time quantitative PCR (qPCR). The *Mili* transcript was significantly more abundant in Td^+ NSCs (10 dpi) than in adult-born Td^+ neurons (30 dpi) or Td^- cells (Fig 1G). These results indicate that Mili expression is dynamic in neurogenesis, being enriched in neural stem/progenitor cells and depleted in their differentiated progeny.

Identification and validation of piRNAs in aNPCs

Next, we used RNA seq. to investigate the presence of endogenous piRNAs in undifferentiated aNPCs (DIF0), or upon onset of vector-induced neurogenesis (DIF4-7). To eliminate the possibility of ribosomal RNA (rRNA) or full-length transfer RNA (tRNA) contaminations, we performed small RNA size selection during library preparation. Following a previously published analysis pipeline (Ghosheh *et al*, 2016), we identified a total of 725,472 putative piRNAs, and using stringent criteria, we focused the subsequent analyses on the 571,439 small noncoding RNAs that perfectly aligned (i.e., no mismatch) with mouse piRNA previously annotated in the piRNA database (piRBase, Zhang *et al*, 2014). Putative piRNAs in aNPCs had a peak length of 30 nt (Fig 2A) and bore a 5′ uridine (U) bias (Fig 2B), in agreement with previous reports in the brain of adult mice (Ghosheh *et al*, 2016). Moreover, the nucleotide-pair distance probability between the 5′ termini of putative primary and secondary piRNAs was distributed similarly to that of other animals (Gainetdinov *et al*, 2018), with asymptotic convergence around the “0” mark on the abscissa (Fig 2C). Mature piRNAs typically bear 2′-*O*-methylation at their 3′ termini, which confers them stability and enables stronger binding to Piwi proteins (Czech *et al*, 2018; Ozata *et al*, 2019). Thereby, we asked whether the endogenous piRNAs isolated from aNPCs were also methylated by evaluating their resistance to periodate oxidation and alkaline ß-elimination, as previously reported (Kirino & Mourelatos, 2007). As controls, we used a synthetic piRNA bearing or lacking, a 2′-O-methylation in its 3′ end. As expected, the unmethylated synthetic piRNA was degraded after sodium periodate treatment, whereas the methylated one was preserved, as indicated by (qPCR)-based small RNA assay (TaqMan) (Fig 2D). Small RNAs isolated from aNPCs were subject to periodate treatment in the same experiment. As expected, endogenous unmethylated small noncoding RNAs, such as snoRNA-202 and -135, were degraded; whereas four of the most abundant endogenous piRNA-cluster consensus sequences (piCS, i.e., extended by qPCR primers bearing specificity for shared sequences among different clusters) identified in aNPCs exhibited resistance to periodate treatment, thus indicating their methylation (Fig 2D). We then addressed the Mili-dependence of the endogenous piRNAs. To this aim, we used three independent strategies to achieve *Mili* KD in aNPCs (Figs 2E and EV1A and B). Specifically, we transduced short-hairpin RNAs targeting *Mili* transcripts through a lentiviral vector, and transfected two different synthetic antisense oligonucleotides (GapmeRs) targeting distinct *Mili* regions (Fig 4A). Indeed, KD of *Mili* in aNPCs was sufficient to deplete four of the most abundant endogenous piCS (Figs 2F and EV1C), in agreement with the observation that Mili is the main Piwi protein in these cells (Fig 1). Of note, this manipulation did not affect *Miwi* expression, excluding possible compensatory effects on piRNA biogenesis in aNPCs (Fig EV1D). PiRNAs interact with Piwi proteins to form functional complexes. To examine whether endogenous Mili and piRNAs associate in aNPCs, we immunoprecipitated (IP) the Mili protein from differentiating aNPCs (DIF4, i.e., at the peak of Mili expression; the

 © 2022 The Authors

specificity of the anti-Mili antibody used for the IP was validated by western blotting of lysates from the testis of Mili null and control adult mice, Fig EV1E). IP with IgG was also included as a control for nonspecific binding (Fig 2G). To determine the size distribution of RNAs co-precipitated with Mili, we used capillary electrophoresis on microfluidic chips. This analysis indicated that the peak size distribution of Mili-bound RNAs in aNPCs was 25 nt (Fig 2H), in agreement with the known size of Mili-bound piRNAs in the mouse testis (Ding *et al*, 2017). Real-time qPCR confirmed that five of the most abundant endogenous piCS identified in aNPCs were enriched in the

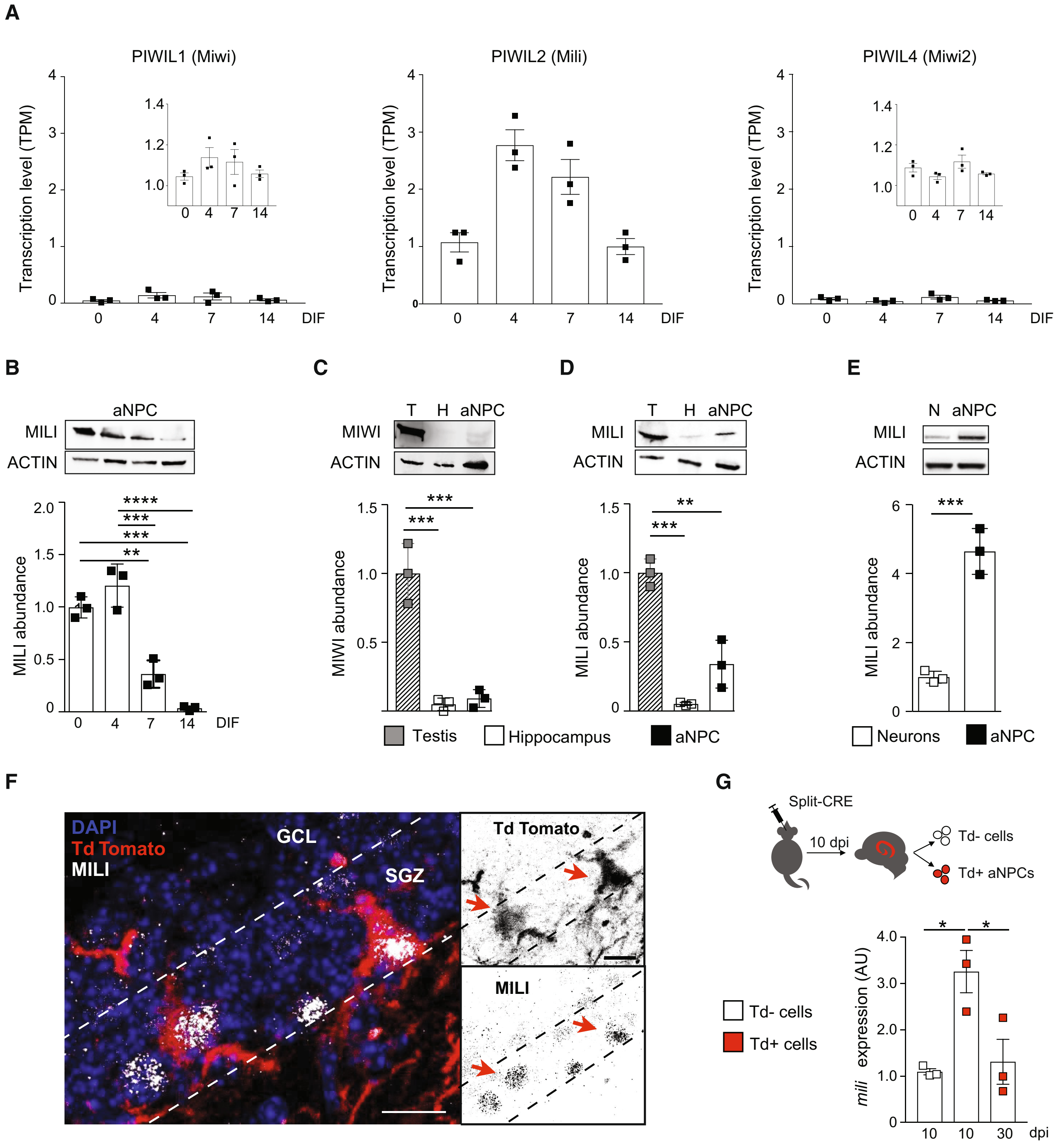

Figure 1.

Figure 1. Mili is preferentially expressed in aNPCs and depleted in neurogenesis.

A Levels of *Piwil1 (Miwi)*, *Piwil2 (Mili)*, and *Piwil4 (Miwi2)* transcripts in RNA seq. data from undifferentiated aNPCs (Days of differentiation—DIF—0) and differentiating neuroblasts (DIF4-14); insets in left and right panels show the same data with smaller scales in the ordinate axes.

B Western blot (inset) and quantification (bar graph) of Mili protein abundance in DIF0 aNPCs and differentiating neuroblasts upon viral-induced neurogenesis (DIF4-14).

C, D Western blot (insets) and quantification (bar graphs) of Miwi and Mili protein abundance in lysates from postnatal mouse testis, hippocampus, and undifferentiated aNPCs cultures.

E Western blot (inset) and quantification (bar graph) of Mili protein abundance in lysates from cultured mouse hippocampal neurons and undifferentiated aNPCs.

F Representative immunofluorescence micrograph of Mili (white) and Td^{+} NSCs (red) in hippocampal subgranular zone (SGZ); arrows indicate Td^{+} $Mili^{+}$ double-positive cells.

G Scheme of the experiment (top) and *Mili* mRNA expression in sorted Td^{+} and Td^{-} cells after *in vivo* transduction with split-Cre viruses in the hippocampus (bottom).

Data information: data are expressed as mean ± SEM, $n = 3$ biological replicates. *$P < 0.05$, **$P < 0.01$, ***$P < 0.001$, ****$P < 0.0001$ as assessed by one-way ANOVA with the Bonferroni test (in B–D, G) and the two-tailed Student's *t*-test (in E). In (F): GCL, granular cell layer; H, Hilus. The scale bars represent 10 μm.
Source data are available online for this figure.

Mili-IP compared with the control IP (Fig 2I). Together these results indicate that Mili and piRNAs are co-expressed and interact in aNPCs.

Expression of piRNAs parallels Mili abundance in neurogenesis

Analysis of small RNAs seq in undifferentiated aNPCs (DIF0), or upon onset of vector-induced neurogenesis (DIF4-7) showed that piRNAs were dynamically expressed, peaking at the onset of differentiation (DIF4) (Figs 3A and EV2A), in agreement with the Mili expression pattern (Fig 1). To validate this observation, we sorted Td^{+} NSCs from the adult hippocampus and quantified levels of four of the most abundant piCS, confirming their expression *in vivo* (Fig 3B). Genomic mapping of the piRNA reads from aNPCs and their progeny identified 298 clusters perfectly aligning to the mouse genome (Fig 3C and Dataset EV2). These clusters had an average length of 168 bases, with some exceeding 2,000 bases, as previously seen in mouse testis (Aravin *et al*, 2006; Girard *et al*, 2006). The piRNA raw reads/cluster averaged around 4,700 reads, with two clusters, one located in chromosome 13 and one in the 17, giving rise to more than 80,000 piRNA reads (Fig 3C). Analysis of small RNA seq data for directionality suggested a strand bias, where the majority of the piRNAs arise unidirectionally, although some piRNAs were found to be homologous to both strands and at different loci. Of note, one of the clusters in our dataset (hereafter referred to piR-cluster 1, Dataset EV2) is homologous to the human piR-61648 that was recently shown to be selectively expressed in somatic tissues but depleted in gonads (Torres *et al*, 2019; Fig EV2B). In agreement, analysis of small RNA datasets from the RIKEN FANTOM5 project (De Rie *et al*, 2017) showed an enriched expression of the piRNAs bearing sequence homology to piR-cluster 1, as well as of many additional piRNA clusters in human NSCs compared with differentiated brain cells (Fig EV2C and Dataset EV3). Together, these results indicate that Mili-dependent piRNAs are more abundant in neural stem/progenitor cells than in their differentiated progeny, thus matching the expression of Mili.

Depletion of Mili and piRNAs impairs neurogenesis and increases astrogliosis

To infer functions of the piRNA pathway in neurogenesis, we KD *Mili* in aNPCs and investigated the consequences of Mili and piRNA depletion for their proliferation, survival, differentiation, and fate. Mili KD did not alter aNPC stemness or proliferation (Fig EV3A), but it led to a dramatic increase in the expression of the astrocyte marker glial fibrillary acidic protein (GFAP) *in vitro* (Fig EV3B). Next, we KD *Mili in vivo* by injecting two different GapmeRs antisense to the *Mili* transcript (GapmeR1 shown in Fig 4; GapmeR3 shown in Fig EV3C), or a scrambled GapmeR (Control) in the DG of postnatal mouse hippocampus (Figs 4A–G and EV3D and E). Inspection of brain sections 30 days after bilateral injections indicated a marked increase in $GFAP^{+}$ cells that showed enlarged somas in the ipsilateral hippocampus injected with GapmeR antisense to *Mili*, compared with the contralateral side injected with control GapmeR (Figs 4D and EV3D). Quantification of GFAP protein (fluorescence intensity) and transcript in Mili KD hippocampus (Figs 4E and EV3D) confirmed this observation. To ascertain whether $GFAP^{+}$ cells were actively generated upon Mili KD, we labeled dividing cells by administration of bromodeoxyuridine (BrdU) in a third cohort of mice, immediately after GapmeRs injection (Figs 4F and EV3E). Thirty days after, we found that Mili KD led to a significant increase in adult-born $GFAP^{+}BrdU^{+}$ glial cells at the expense of $NeuN^{+}BrdU^{+}$ neurons (Figs 4F and EV3E). This result was corroborated by RNA seq dataset analysis from differentiating aNPCs *in vitro*, showing an enrichment in the expression of astrocyte-related genes and a concomitant deregulation of neuronal fate genes upon Mili KD (Fig EV3F). These results indicate that Mili sustains neurogenesis in the postnatal hippocampus, at the expense of gliogenesis. Increased GFAP expression is generally regarded as a hallmark of astrocytic reactivity (Escartin *et al*, 2021). In agreement, we observed a significant increase in the levels of known reactive glial markers (Liddelow *et al*, 2017; Clarke *et al*, 2018) upon Mili KD in the postnatal hippocampus (Fig 4G). To corroborate this result, we took advantage of Kainic Acid (KA) injection in the postnatal hippocampus of mice expressing GFP under the control of the NSCs/NPCs specific promoter *Nestin* (Fig 4H), a treatment previously shown to induce aNSC conversion into reactive glia (Sierra *et al*, 2015; Bielefeld *et al*, 2017). Indeed, this treatment reduced the levels of *Mili* and one of the most abundant piCS-bearing sequence homology to piR-cluster 1 in sorted *Nestin*-GFP^{+} NSCs (Fig 4H). Altogether, these results demonstrate that Mili functions are essential for proper neurogenesis and prevent reactive gliogenesis in the postnatal mouse hippocampus.

 © 2022 The Authors

Depletion of Mili and piRNAs in aNPCs results in senescence-associated phenotypes

Conversion of hippocampal NSC into reactive glia at the expense of neurogenesis has been related to increased neuroinflammation and cellular senescence (Martín-Suárez *et al*, 2019; Babcock *et al*, 2021), and it has been observed in normal aging (Clarke *et al*, 2018). The involvement of the piRNA pathway in these mechanisms is unknown. We investigated whether the depletion of Mili and piRNA induces senescence-associated secretory phenotype (SASP), β-galactosidase activity (β-gal) and cell cycle exit, known hallmarks of an aged hippocampal niche (Ahlenius *et al*, 2009; Encinas *et al*, 2011; Jin *et al*, 2021). In agreement with the *in vivo* phenotypes, Mili KD in aNPCs cultures induced a significant increase in the expression of several genes encoding immune-modulatory and SASP proteins in neuroblasts (DIF4), compared with control cells (Fig 5A). Moreover, Mili KD resulted in a higher proportion of cells positive for β-gal as early as 48 h upon induction of their spontaneous differentiation (Fig 5B). At the same time point, we immunostained aNPCs with anti-KI67 (a protein that is expressed in all phases of the cell cycle except G0 and early G1; Yu, 1992) and anti-BrdU antibodies. Quantification of the $BrdU^{+}$ and $KI67^{-}$ cells over

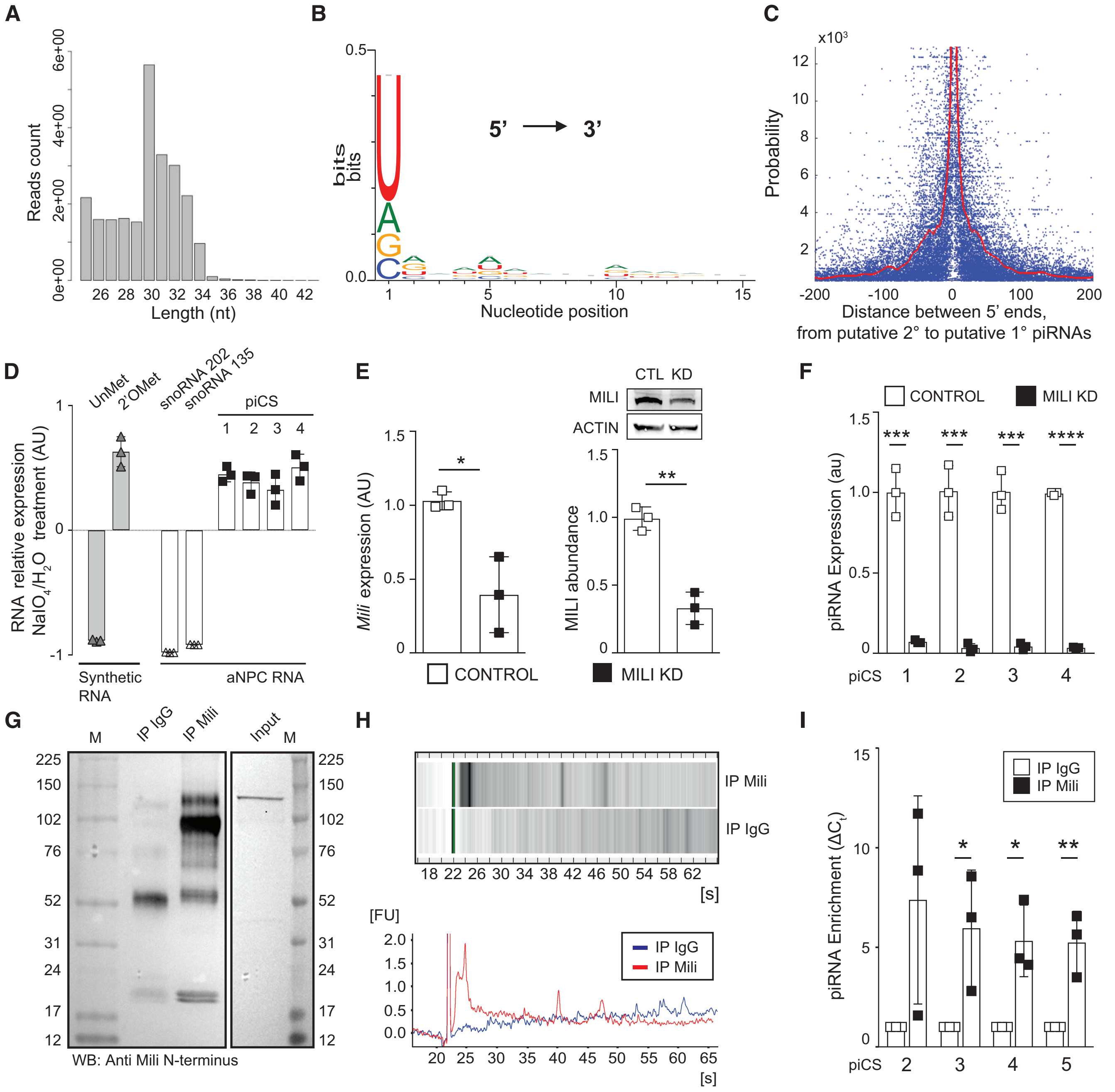

Figure 2.

Figure 2. Identification and validation of piRNAs in aNPCs.

A–C (A) Size distribution of the piRNA reads showing (B) uridine bias at piRNA 5′ ends and (C) probability of distances from the 5′ ends of putative secondary piRNAs to the 5′ ends of putative primary piRNAs. Note in panel C that 5′ termini of putative primary and secondary piRNAs derived from the same cluster tend to concatenate around the "0" mark, as reported in other animals. Distance probability was assayed for unique piRNAs (length between 15–35 nucleotides), without taking into account abundance, by locally weighted smoothing linear regression (LOWESS).

D Relative expression of transcripts bearing piRNA-cluster consensus sequences (piCS) of the top abundant piRNAs, and two control snoRNAs (202 and 135) in aNPC, upon treatment with sodium periodate ($NaIO_4$) or water and alkaline ß-elimination. Synthetic RNA oligos were used as negative (Unmethylated, UnMet) and positive (2′-O-methylated, 2′OMet) controls, respectively. Note that the presence of 3′-end 2′-O-methylation in positive control and piRNAs confers them resistance to periodate oxidation and alkaline ß-elimination, in contrast to the depletion in UnMet negative control and snoRNAs.

E *Mili* mRNA expression (left bar graph); western blot (inset) and quantification (right bar graph) of Mili protein abundance in aNPCs upon viral transduction of scrambled shRNA (Control) or shRNA targeting *Mili* (Mili KD).

F Expression of four of the most abundant piCS in control and Mili KD aNPCs.

G–I Western blot (G), analysis by capillary electrophoresis (H), or quantification by qPCR (I) of the endogenous piCS after co-immunoprecipitation (IP) with endogenous Mili (IP Mili), or control IgG (IP IgG) in lysates of DIF4 aNPCs. In the qPCR abundance of the indicated piCS in the IP Mili was normalized to its respective level in the control co-immunoprecipitation (IgG); error bars in (I) represent standard deviation.

Data information: data are expressed as mean ± SEM unless differently indicated, $n = 2$ biological replicates (A–C) and $n = 3$ biological replicates (D–F, I). *$P < 0.05$, **$P < 0.01$, ***$P < 0.001$, ****$P < 0.0001$, as assessed by the two-tailed Student's *t*-test.

Source data are available online for this figure.

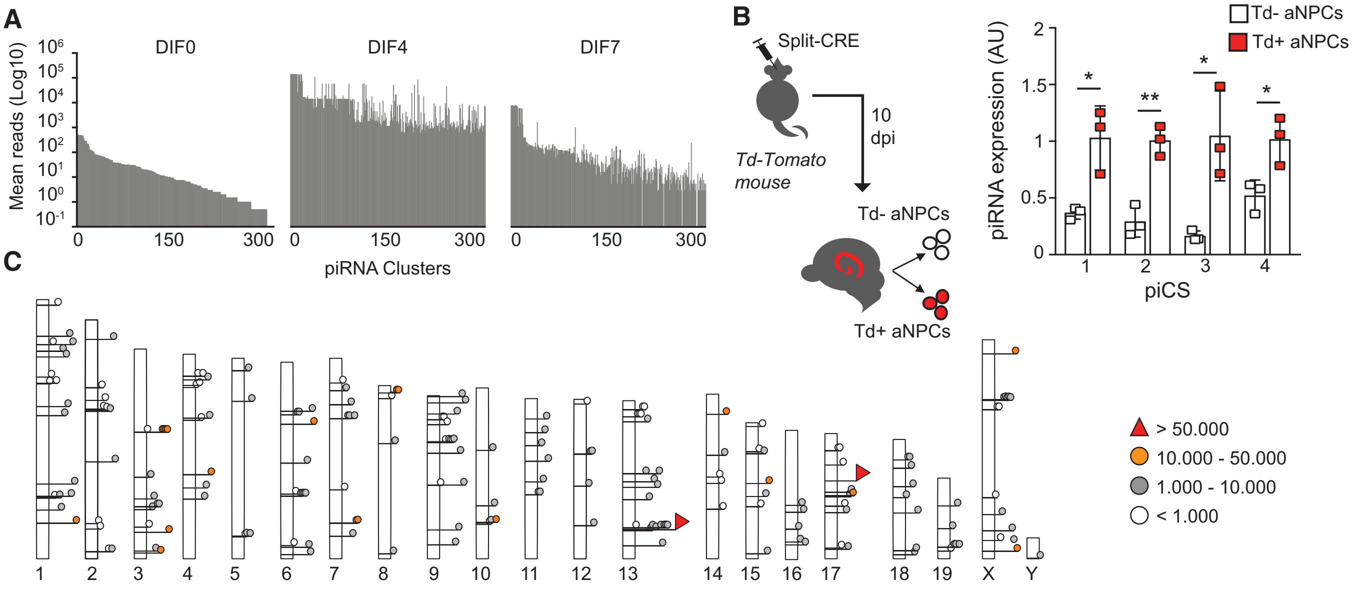

Figure 3. Expression of piRNAs parallels Mili abundance in neurogenesis.

A Mean reads of the piRNA clusters in undifferentiated aNPCs (DIF0) and neuroblasts (DIF4-7) upon viral-induced neurogenesis, where the total number of filtered reads in each library ranges between $1.4–4.4 \times 10^6$.

B (left) Schematic representation of the experiment; (right) expression of four of the most abundant piCS in sorted Td^+ and Td^- cells 10 dpi of split-Cre viruses in hippocampus.

C Genomic location of the 298 piRNA clusters found in the *Mus musculus* genome (assembly MGSCv37; mm9).

Data information: data are expressed as mean ± SEM, $n = 2$ biological replicates (A, C) and $n = 3$ biological replicates (B). *$P < 0.05$, **$P < 0.01$, as assessed by the two-tailed Student's *t*-test carried out for each piCS between Td^+ and Td^- cells.

total $BrdU^+$ indicated a premature cycle exit upon Mili KD (Fig 5C). Similarly, by propidium iodide incorporation and flow cytometry analysis we found a significant increase in the proportion of cells in G0/G1 phase and a concomitant decrease in S phase cells upon Mili KD (Fig 5D; Mili KD G0/G1 = 89.8%, S = 6.03%, G2/M = 4.03%; Control G0/G1 = 84.7%, S = 8.97% G2/M = 6.27%). Mili KD did not lead to apoptosis (Fig EV4A and B), whereas it led to altered expression of genes encoding proteins associated with oxidative stress, circadian mechanism (Fig EV4C and D, and Dataset EV4), and senescence-induced cell cycle exit (Fig 5E and F) in agreement with previous reports (Schouten *et al*, 2020; Adusumilli *et al*, 2021; Babcock *et al*, 2021). Strengthening this evidence, we found increased β-gal activity in the ipsilateral hippocampus injected with different GapmeRs antisense to *Mili*, compared with the contralateral side injected with control GapmeR (Figs 5G and EV4E and F). Next, we sorted *Nestin*-GFP^+ NSCs from the DG of young (6 weeks) and ~12 months old mice (i.e., when the majority of hippocampal NSCs turn into an aged phenotype; Martín-Suárez *et al*, 2019) and found

© 2022 The Authors

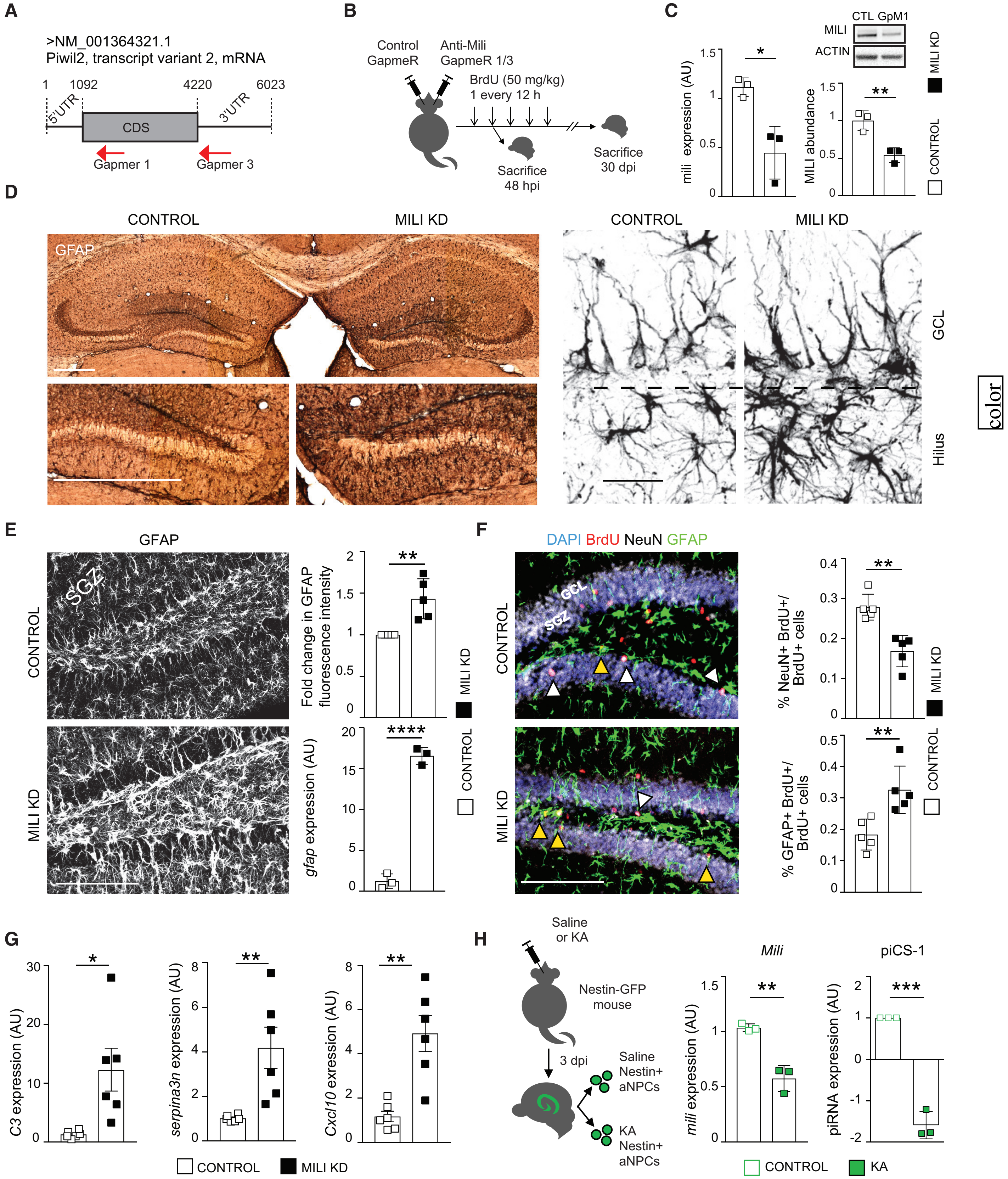
A
>NM_001364321.1
Piwil2, transcript variant 2, mRNA
1 1092 4220 6023
5'UTR CDS 3'UTR
Gapmer 1 Gapmer 3
B
Control GapmeR
Anti-Mili GapmeR 1/3
BrdU (50 mg/kg) 1 every 12 h
Sacrifice 48 hpi
Sacrifice 30 dpi
C
CTL GpM1
MILI
ACTIN
mili expression (AU)
MILI abundance
MILI KD
CONTROL
D
CONTROL MILI KD
GFAP
CONTROL MILI KD
GCL
Hilus
E
GFAP
CONTROL
MILI KD
SGZ
Fold change in GFAP fluorescence intensity
gfap expression (AU)
F
DAPI BrdU NeuN GFAP
GCL
SGZ
% NeuN+ BrdU+/ BrdU+ cells
% GFAP+ BrdU+/ BrdU+ cells
G
C3 expression (AU)
serpina3n expression (AU)
Cxcl10 expression (AU)
CONTROL MILI KD
H
Saline or KA
Nestin-GFP mouse
3 dpi
Saline Nestin+ aNPCs
KA Nestin+ aNPCs
Mili
piCS-1
mili expression (AU)
piRNA expression (AU)
CONTROL KA

Figure 4.

Figure 4. Depletion of Mili and piRNAs impairs neurogenesis and increases astrogliosis.

A Representation of the targeting regions of GapmeR1 and 3 on the *Mili* transcript.
B Scheme of the *in vivo* experiment.
C *Mili* mRNA expression (left bar graph); western blot (inset) and quantification (right bar graph) of Mili protein abundance in lysates from the DG of mouse hippocampi 48 h after the injection of scrambled (Control) or GapmeR1 against *Mili* (Mili KD).
D Representative light microscopy (left) and confocal (right) micrographs of postnatal hippocampal sections, immunostained for GFAP at 30 dpi of scrambled (Control, left hemisphere) and GapmeR1 against *Mili* (Mili KD, right hemisphere).
E Representative immunofluorescence micrograph of postnatal hippocampal sections immunostained for GFAP at 30 dpi of scrambled (Control) and GapmeR1 against *Mili* (Mili KD); Right panels: Fold change in GFAP fluorescence intensity level (upper graph) in a hippocampal region of interest (ROI) of 500 μm^2 in brain slices upon Mili KD compared with Control; *Gfap* mRNA levels (lower graph) in the DG from mouse hippocampi 48 h after the injection of scrambled (Control) or GapmeR1 (Mili KD).
F (left) Representative immunofluorescence micrograph of postnatal hippocampal sections immunostained for GFAP (green), BrdU (red), NeuN (white), and nuclear DNA (blue) at 30 dpi of scrambled (Control) or GapmeR1 against *Mili* (Mili KD); (right graphs) percentages of $NeuN^+BrdU^+$ (white arrowheads in the images), or $GFAP^+BrdU^+$ (yellow arrowheads in the images) double-positive cells over total $BrdU^+$ cells.
G Relative mRNAs expression of reactive astrocyte markers in the hippocampus 48 h upon injection of scrambled (control, $n = 6$ mice) or GapmeRs against *Mili* (Mili KD, $n = 3$ mice GapmeR1 and $n = 3$ GapmeR3).
H (left) Schematic representation of the experiment; *Mili* mRNA (left graph) and piCS1 (right graph) expression in sorted GFP^+ NSCs from *Nestin*-GFP mice treated with Saline (Control) or Kainic Acid (KA).

Data information: data are expressed as mean ± SEM, $n = 3$ (C, D, E mRNA, H) and 5 (E, F) biological replicates. *$P < 0.05$, **$P < 0.01$, ***$P < 0.001$, ****$P < 0.0001$, as assessed by the two-tailed Student's *t*-test. UTR, untranslated region; CDS, coding sequence; GCL, granular cell layer; SGZ, subgranular zone. The scale bars represent 1 mm (D, left), 10 µm (D, right), and 100 µm (E, F).
Source data are available online for this figure.

that *Mili* transcript was significantly reduced in aged NSCs compared with the young (Fig 5H). These results indicate that Mili functions likely prevent the senescence of aNPCs and their progeny.

Identification of piRNA targets in neurogenesis

Next, we sought to identify targets of piRNAs in aNPCs lineages. In contrast to germline piRNAs, which primarily target TEs, somatic piRNAs have also homology with, or pair by sequence complementarity, to a variety of noncoding RNAs including tRNAs and others from repetitive elements (Keam *et al*, 2014; Rojas-Ríos & Simonelig, 2018). Thereby, we first performed *in silico* prediction of noncoding RNAs targeted by the piRNAs identified in our model. Accordingly, TEs were just a minor percentage of the predicted noncoding RNA targets in both undifferentiated aNPCs and progeny (Fig 6A and B), despite their proportion being increased upon induction of neurogenesis (Fig 6B). The latter finding is in agreement with the activation of TEs (e.g., LINE1) observed during neuronal differentiation (Muotri *et al*, 2005; Coufal *et al*, 2009; Upton *et al*, 2015). Interestingly, transcripts from repeats such as 5S rRNA and tRNAs were the main predicted targets in both undifferentiated (47% and 40%, respectively) and progeny (35% and 16%, respectively, Fig 6A and B). To ascertain whether these noncoding RNAs are modulated upon Mili and piRNA depletion, we quantified their levels in Mili KD aNPCs and progeny (Fig 6C). Indeed, Mili depletion significantly elevated levels of 5S rRNA and SINEB1 family of TEs in both undifferentiated aNPCs and progeny, compared with scrambled control (Fig 6C), whereas LINE1, here quantified with a qPCR assay detecting the full-length transcript, was initially refractory to Mili depletion and its level only increased late in differentiation (Fig 6C).

To identify which protein-coding transcripts are modulated upon Mili depletion in aNPCs lineages, we analyzed RNA seq data from Mili KD or scrambled control cells during their spontaneous

Figure 5. Depletion of Mili and piRNAs in aNPCs results in senescence-associated phenotypes.

A Heatmap illustrating the expression of genes encoding proteins involved in immune-modulatory and senescence-associated phenotype in differentiating neuroblasts (DIF4) upon Mili KD, compared with control cells. Expression heatmap correlation plots were computed by the k-means clustering method. Scale bar indicates *Z*-scores.
B Representative bright-field microscopy images (left) and quantification (right) of $ß\text{-galactosidase}^+$ aNPCs as percent of total cells upon Mili KD, or control 48 h after induction of spontaneous differentiation.
C Representative fluorescence microscopy images (left) and quantification (right) of control or Mili KD neuroblasts 48 h after spontaneous differentiation, immunostained with anti-BrdU (white) and Ki67 (purple) antibodies. (Right) Percentage of $BrdU^+$ and $Ki67^-$ cells over $BrdU^+$ cells.
D Representative cell cycle analysis of propidium iodide staining by flow cytometry in neuroblasts 48 h after spontaneous differentiation; Percentage of cells in G0/G1 and S is reported in the text.
E Relative mRNA expression of genes encoding proteins involved in the regulation of cell cycle in control and Mili KD neuroblasts 48 h after spontaneous differentiation.
F Transcript abundance expressed in transcripts per million (TPM) of genes encoding proteins involved in the regulation of cell cycle and differentiation (left) or cell cycle (right) in DIF4 neuroblasts upon Mili KD, compared with control cells.
G Representative light-microscopy images of the ß-galactosidase staining of postnatal hippocampal sections, 30 dpi of scrambled (Control, left hemisphere) and GapmeR1 against *Mili* (Mili KD, right hemisphere). See Fig EV4E for a larger image of this section.
H (left) Scheme of the *in vivo* experiment; (right) *Mili* mRNA expression in *Nestin*-GFP^+ sorted cells from young (6 weeks) and old (56 weeks) mice.

Data information: data are expressed as mean ± SEM, $n = 3$ (A, D–F, H) and 5 (B, C) biological replicates (in A each biological replicate was sequenced with two separate flow cells). *$P < 0.05$, **$P < 0.01$, ***$P < 0.001$, ****$P < 0.0001$, as assessed by the two-tailed Student's *t*-test. The scale bars represent 50 µm (B, C) and 500 µm (G).

 © 2022 The Authors

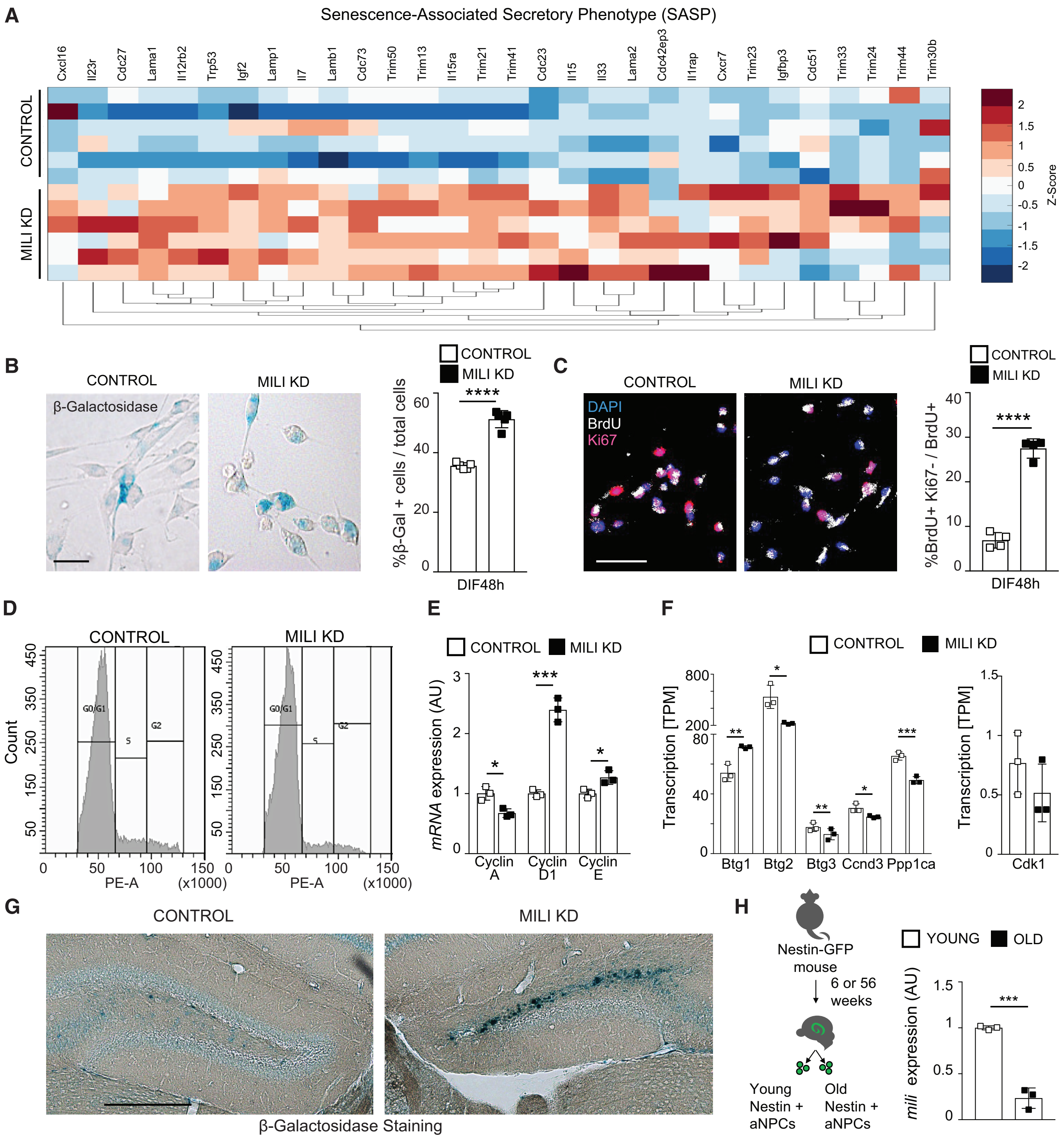

Figure 5.

differentiation, at the peak of Mili and piRNA expression (i.e., DIF4). Most of the transcripts modulated upon Mili depletion were upregulated (Fig 6D), and most of them bore sequences antisense to piRNAs and/or harbored homologous sequences to piRNAs (Fig 6D and E). More specifically, we observed that transcripts from individual genes are targeted by multiple unique piRNAs, with a maximum of 11,904 piRNAs targeting a single modulated gene (Fig 6F), and a maximum of 9,870 piRNA transcripts per million (TPM) targeting a single mRNA (Fig 6G).

To address possible functions of the piRNA targets in aNPCs we searched the Gene ontology (GO) and Kyoto Encyclopedia of Genes and Genomes (KEGG) databases. GO analysis of the upregulated protein-coding targets upon Mili depletion indicated a prevalence of genes involved in the regulation of chromatin, transcription, mRNA

© 2022 The Authors

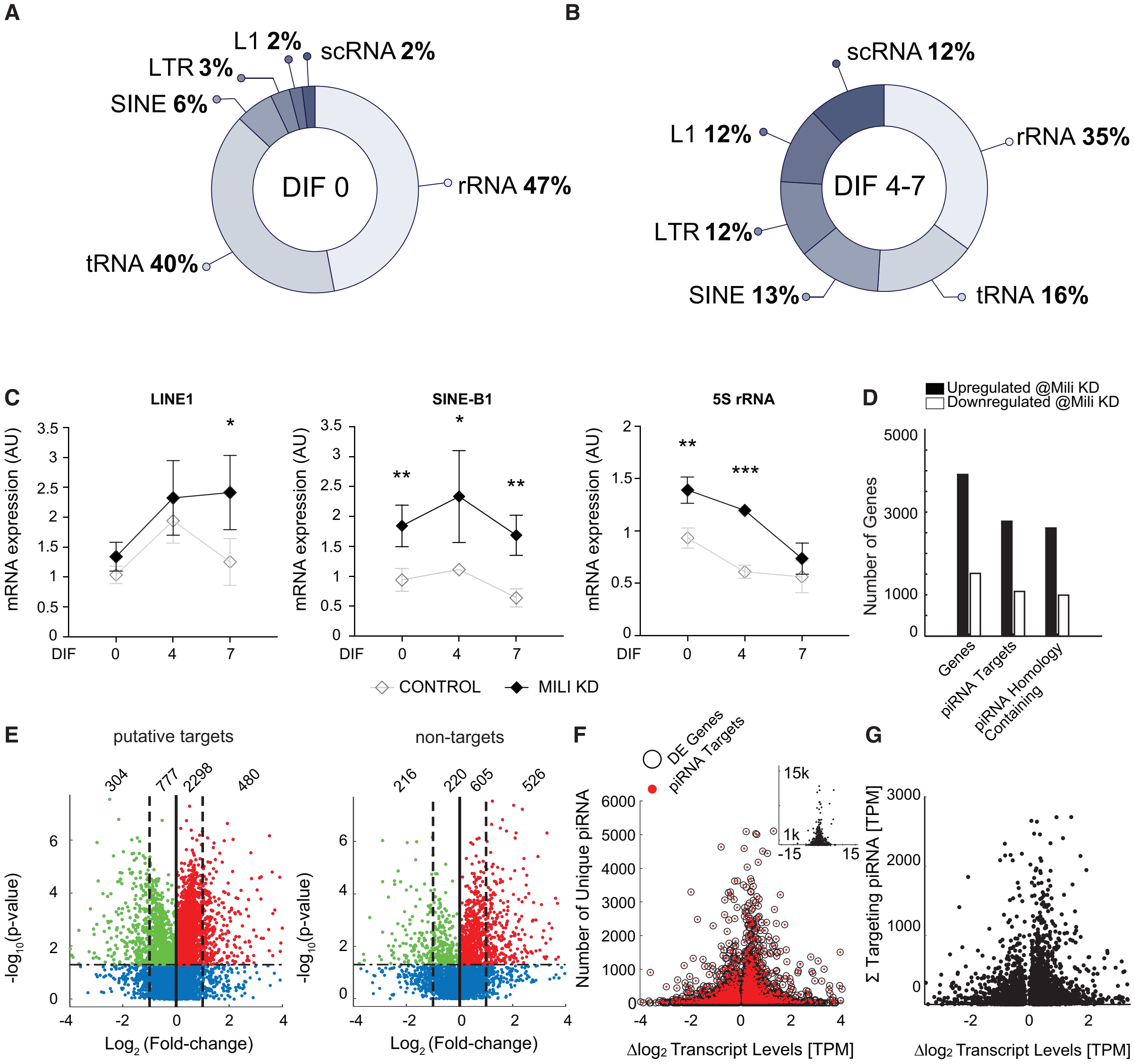

Figure 6. piRNAs target repetitive elements and mRNAs in neurogenesis.

A, B Pie plots showing proportions of noncoding RNAs predicted targets of piRNAs in undifferentiated aNPCs (DIF0) and differentiating neuroblasts (DIF4-7).

C Transcript levels of *LINE1* 5′UTR, *SINEB1*, and *5S rRNA* in the Mili KD or control aNPCs (DIF0) and differentiating neuroblasts (DIF4-7).

D Total counts of upregulated (black bars) and downregulated protein-coding genes (white bars) in Mili KD versus scrambled control neuroblasts at DIF4.

E Volcano plots showing the log2 fold change of significantly altered mRNA transcripts (numbers of each category indicated) that are target or nontarget of the piRNAs.

F The log2 fold change of significantly altered mRNA transcripts (abscissa) plotted with the raw number of unique piRNA sequences qualified as targeting molecules (ordinate) for all modulated genes (black circles), and piRNA-targeted genes (red dots), identified by RNA seq. The modulated genes without piRNA target sequences concatenate at the bottom at the "$y = 0$" value; total range, inset.

G The log2 fold change of significantly altered piRNA-targeted mRNA transcripts (abscissa) plotted with the summed levels of all mRNA-targeted (complementary) piRNA molecules (ordinate), identified by RNA seq.

Data information: data are expressed as mean ± SEM, $n = 3$ (C) biological replicates. Data are expressed in transcripts per million (TPM) as the mean levels of six sequencing runs (three biological replicates sequenced with two separate flow cells) profiles at DIF4 (D–G). Outliers (mean calculation) were detected by more than three mean absolute deviations, for a final "*n*" value between 4 and 6 samples. **P* < 0.05, ***P* < 0.01, ****P* < 0.001, as assessed by the two-tailed Student's *t*-test. (G) The two-sample Kolmogorov–Smirnov test on the change in expression between piRNA target and nontarget genes, *P*-value = 3.21e-260.

 © 2022 The Authors

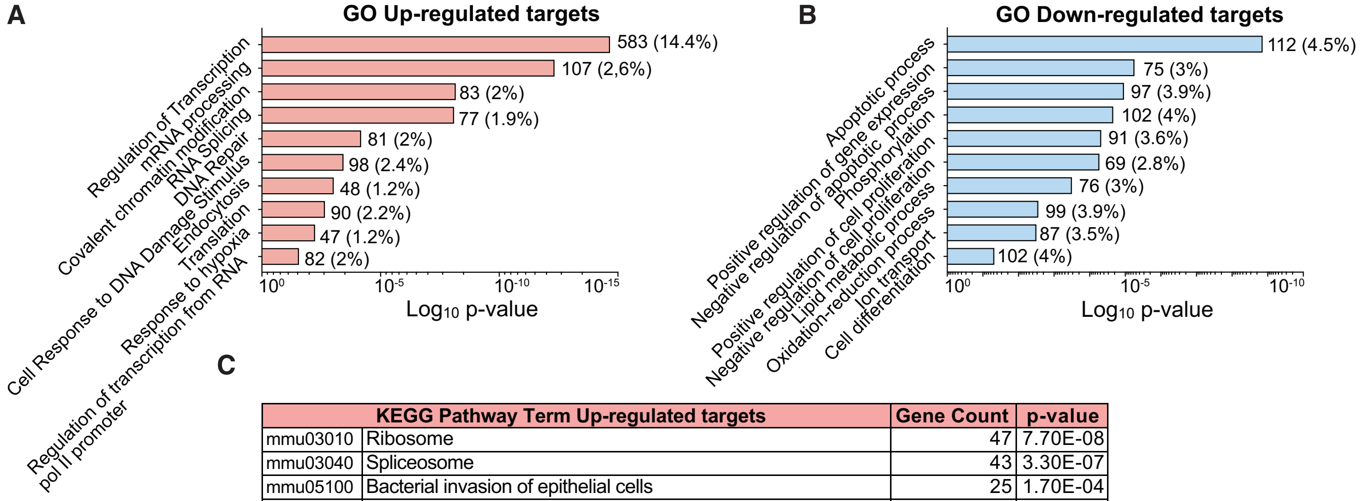

C

KEGG Pathway Term Up-regulated targets		Gene Count	p-value
mmu03010	Ribosome	47	7.70E-08
mmu03040	Spliceosome	43	3.30E-07
mmu05100	Bacterial invasion of epithelial cells	25	1.70E-04
mmu04810	Regulation of actin cytoskeleton	49	8.90E-04
mmu05412	Arrythmogenic right ventricular cardiomyopathy (ARVC)	21	8.90E-14
mmu05033	Nicotine addiction	15	9.40E-04
mmu03018	RNA degradation	24	1.00E-03
mmu04510	Focal adhesion	47	1.50E-03
mmu05213	Endometrial cancer	17	2.00E-03
mmu04670	Leukocyte transendothelial migration	30	2.30E-03

KEGG Pathway Term Down-regulated targets		Gene Count	p-value
mmu05166	HTLV-I infection	58	5.10E-07
mmu04390	Hippo signaling pathway	38	7.90E-07
mmu04510	Focal adhesion	46	1.90E-06
mmu04010	MAPK signaling pathway	50	1.70E-05
mmu05200	Pathways in cancer	68	6.20E-05
mmu04115	p53 signaling pathway	19	1.60E-04
mmu05205	Proteoglycans in cancer	40	1.70E-04
mmu04921	Oxytocin signaling pathway	32	2.00E-04
mmu05203	Viral carcinogenesis	43	3.40E-04
mmu04151	PI3K-Akt signaling pathway	59	3.90E-04

Figure 7. Analysis of genes and pathways modulated upon Mili and piRNA depletion.

A, B Bar graph showing the top biological pathways of significantly upregulated (A) and downregulated (B) protein-coding genes in Mili KD neuroblasts at DIF4; Numbers in each category indicate gene counts and percentages are normalized on the total number of genes for each category.

C Most significant terms generated from KEGG pathway analysis of the modulated targets.

processing, translation, and DNA repair (Fig 7A), which are well-known functions regulated by the piRNA pathway in both germline and somatic tissues (Czech *et al*, 2018; Rojas-Ríos & Simonelig, 2018; Ozata *et al*, 2019). Among the downregulated protein-coding targets, we found genes involved in the regulation of apoptosis, cell proliferation, oxidative pathway, and differentiation (Fig 7B), in agreement with the main phenotypes that we observed upon Mili depletion in neurogenesis. "Ribosome" and "Spliceosome" were the top terms among upregulated genes in the KEGG pathway analysis; whereas, cancer-related terms were common among the downregulated pathways (Fig 7C), in agreement with the known oncogenic role of Piwil2 in various human tumors (Lee *et al*, 2006). As 5S rRNA and SINEB1, both involved in the control of ribosome biogenesis and translation, are among the piRNA targets in aNPCs and because the dysregulation of ribosome biogenesis has been associated with cellular senescence (Liu & Sabatini, 2020), we investigated ribosome density and translation in Mili KD aNPCs and progeny. Accordingly, Mili depletion increased the density of polyribosomes in both undifferentiated and differentiating aNPCs compared with control cells (Fig EV5A and B), as revealed by immunostaining for the ribosomal protein RPL26 imaged with stimulated emission depletion (STED) nanoscopy (Viero *et al*, 2015). Furthermore, the protein synthesis rate, measured by OPP (O-propargyl-puromycin) labeling of nascent proteins, was significantly increased upon Mili depletion in differentiating neuroblasts (DIF7), but not in undifferentiated aNPCs (DIF0) (Fig EV5C), in agreement with the notion that the density of ribosomes over a transcript does not necessarily correlate with its translation (Mills & Green, 2017).

In sum, these results indicate that the piRNA pathway is present and preferentially expressed in adult hippocampal NSC/NPCs compared with their progeny, where it exerts a wide range of gene-modulatory functions essential for their fitness and neurogenesis.

Discussion

This study provides the first evidence of the role of the piRNA pathway in neurogenesis. By investigating the presence of Mili and Mili-dependent piRNAs in aNPCs and by inferring functions of this

© 2022 The Authors

pathway in the regulation of adult hippocampal neurogenesis, we provide evidence of an essential role for Mili in maintaining NSC fitness and proper fate. This finding adds a new layer of complexity to the understanding of adult brain plasticity and entails implications for aging and neuronal disorders, where dysregulated expression of the piRNA pathway has been reported, such as neurodegeneration (Jain *et al*, 2019; Wakisaka, 2019) and various psychiatric conditions (Page *et al*, 2021).

At the functional level, the Piwi proteins in gonads and bone marrow maintain stem cell pools by preserving their fitness (De Luca *et al*, 2016; Rojas-Ríós & Simonelig, 2018). Similarly, our data indicate that Mili is required to maintain the fitness of NSC/NPCs in the adult hippocampus. We find that piRNAs are enriched in aNPCs and their expression is dynamic along neurogenesis, mirroring Mili. Moreover, Mili depletion in aNPCs leads to premature cell cycle exit, increases generation of reactive glia, and alters the expression of several inflammatory, reactive oxygen, and circadian-related genes, which are known hallmarks of an aged hippocampal niche (Bonaguidi *et al*, 2011; Encinas *et al*, 2011; Clarke *et al*, 2018; Martín-Suárez *et al*, 2019; Schouten *et al*, 2020); accordingly, we find a reduced expression of Mili in hippocampal NSCs of aged mice. In contrast to the dispensable role of Piwi proteins for hematopoiesis (Nolde *et al*, 2013), however, Mili is required for the proper neurogenesis fate. Thereby, our data support the idea that Mili regulates the fate choice in the hippocampal niche, implicating functions of the piRNA pathway in the maintenance of lifelong neurogenesis, possibly to prevent or delay its drift toward reactive gliogenesis. While some of these conclusions require further longitudinal investigations in *Mili* knockout mice (ideally, bearing Lox-P sites to selectively restrict the deletion of *Mili* to distinct NSC subpopulations), this study underpins a possible involvement of the piRNA pathway in brain plasticity and aging.

At the mechanistic level, several unresolved questions arise from this study. Perhaps the most crucial one is whether Mili functions in neurogenesis are mediated through piRNAs. Indeed, Piwi proteins regulate targets at both the transcriptional and post-transcriptional levels. However, these mechanisms are very complex and differ according to the cell type, the Piwi proteins involved, effectors, and their subcellular localization and do not necessarily require cleavage-competent Piwi or piRNAs (Czech *et al*, 2018; Ozata *et al*, 2019). Our data indicate the presence of key players of the piRNA pathway, such as Mili, several cofactors, as well as piRNAs (fulfilling most of the criteria characterizing them, such as length, U-Bias, 2′-O-Methylation at their 3′ ends, inter-distance, Mili-dependence and -interaction) in aNPCs. We also uncover that this pathway targets, either directly or indirectly, more than 6,000 genes in neurogenesis including repetitive elements, and several genes are known to be regulated by piRNAs in other tissues and cell types; however, given this level of complexity, the mechanisms mediating gene-regulatory functions of the Mili-piRNA complexes in our model certainly warrant further investigation. Future exploitation of the "simplified" aNPC model (where Mili is the main Piwi protein, while other cofactors such as the testis-specific methyl transferase Hen1 are missing), would disentangle the mechanism of piRNA biogenesis and function in neurogenesis, providing the biological understanding of scientific and therapeutic value for brain plasticity and successful aging.

Materials and Methods

Reagents and Tools table

Reagent/ resource	Reference or source	Identifier or catalog number
Experimental models		
C57BL/6J (*M. musculus*)	Jackson Lab	B6.129P2Gpr37tm1Dgen/J
Td-Tomato$^{flox/wt}$ (*M. musculus*)	Jackson Lab	Madisen *et al* (2010)
Mili null mice (*M. musculus*)	European mouse mutant archive (EMMA)	Di Giacomo *et al* (2013)
Nestin-GFP (*M. musculus*)	C. Fitzsimons' Lab	Mignone *et al* (2004)
Antibodies		
Mouse-anti-MILI	Santa Cruz 1:100 WB	Cat # sc-377347
Mouse-anti-MILI	Santa Cruz 1:100 WB, 1:25 IP	Cat # sc-377258
Rabbit-anti-MILI	G. Hannon's Lab 1:150 WB, 1:100 IF/IHC	
Rabbit-anti-MIWI	G. Hannon's Lab 1:200 WB	
Rabbit-anti-ACTIN	Abcam 1:1,000 WB	Cat # ab13970
Rabbit-anti-GADPH	Santa Cruz 1:1,000 WB	Cat # sc-25778
Rabbit-anti-GFAP	Dako 1:1,000 WB, IF/IHC	Cat # Z-0334
Rat-anti-BrdU	Abcam 1:200 IF/IHC	Cat # ab6326
Rabbit-anti-KI67	Abcam 1:250 IF/IHC	Cat # ab15580
Mouse-anti-NeuN	Millipore 1:250 IF/IHC	Cat # MAB377
Rabbit-anti-RPL26	Abcam 1:500 IF/IHC	Cat # ab59567
Mouse-anti-Nestin	Millipore 1:250 IF/IHC	Cat # MAB353
Rabbit-anti-cleaved caspase-3	Cell Signaling Technology 1:400 IF/IHC	Cat # 9664
IgG	Millipore-Sigma 1:100 IP	

Methods and Protocols

Experimental mice

C57BL/6 and Td-Tomato$^{flox/wt}$ knock-in reporter mice (Jackson Laboratory stock number 007908) (Madisen *et al*, 2010) and *Mili* null mice (Di Giacomo *et al*, 2013) were housed at *Istituto Italiano di Tecnologia* (IIT); Nestin-GFP mice (Mignone *et al*, 2004) were housed at the Swammerdam Institute for Life Sciences, University of Amsterdam, The Netherlands. All animal procedures were approved by the IIT animal use committee and the Italian Ministry of health, or by the Commission for Animal Welfare at the University of Amsterdam (DEC protocol 4925, AVD1110020184925), respectively, and

© 2022 The Authors

conducted in accordance with the Guide for the Care and Use of Laboratory Animals of the European Community Council Directives. All mice were group-housed under a 12-h light–dark cycle in a temperature and humidity-controlled environment with *ad libitum* access to food and water.

Virus and GapmeR injection

Split-cre viruses or GapmeRs injection was done as previously published (Pons-Espinal *et al*, 2019); briefly, 8 weeks-old mice Td-Tomato$^{flox/wt}$ or WT C57BL6/J were anesthetized with isoflurane and 1.5 µl of virus mix (Split-Cre N-Cre:C-Cre), or 1.5 µl of 50 µM antisense GapmeR targeting *Mili* or negative control (custom design probes, MILI 339512, Control 339516, Qiagen), were stereotaxically injected in the dentate gyrus. Bilateral injection of Control GapmeR in the left hemisphere and Mili KD GapmeR in the right hemisphere allowed the analysis of the phenotypes within the same brain. To assess the GapmeRs uptake and the efficacy of *Mili* KD, the first group of mice ($n = 3$ for each GapmeR) was sacrificed 48 h after the injection and the DG and tissue processed for RNA or protein extraction. Another set of animals received 2 BrdU intraperitoneal injections per day for 5 days (50 mg/kg) (one every 12 h) starting 24 h after GapmeRs injection. Animals were sacrificed 30 days after GapmeRs injection ($n = 5$ for each oligo) and tissue processed for histological analysis (Pons-Espinal *et al*, 2019). GapmeR sequences are listed in the Table EV1.

Kainic acid (KA) administration and aNPC collection by fluorescence-activated cell sorting (FACS)

Kainic acid to elicit tonic, nonconvulsive epileptic seizures, was administered as described before (Bielefeld *et al*, 2019). Briefly, 50 nl of 2.22 mM Kainic Acid dissolved in PBS (pH 7.4) was injected bilaterally into the hippocampus at the following coordinates (AP −2.0, ML +/− 1.5, DV −2.0 mm) (between 9 AM and 1 PM). Control animals were administered saline (pH 7.4). Bilateral dentate gyri from three animals per condition were pooled to allow sufficient recovery of NSC/NPCs. A single-cell suspension was created using a Neural Tissue Dissociation kit (Miltenyi Biotec), according to the manufacturer's protocol. In order to enrich aNPCs from the DG, we used the endogenous GFP expression driven by the Nestin promotor in combination with FACS. Propidium Iodide (5 µg/ml) was added to the single-cell suspension to assess cell viability. Cells were sorted using a FACSAriatm III system (BD) with a 488 nm excitation laser. Cell duplets were removed based on forward and side scatters, and viable cells were selected based on PI negativity. GFP-positive (corrected for autofluorescence) cells were sorted (≅ 50,000 cells/pool) and collected in PBS containing 1% FBS. Trizol LS (Thermo Scientific) was added and after resuspension samples were snap-frozen and stored at −20°C.

For RNA extraction and cDNA preparation, Td-Tomato$^{flox/wt}$ or Nestin-GFP mice were used. Six to ten Td-Tomato$^{flox/wt}$ mice were euthanized 10 or 30 days after the split-Cre virus injection. DG cells were dissociated with the Neural Tissue Dissociation Kit P (Miltenyi Biotec) and FACS-sorted as previously published (Pons-Espinal *et al*, 2019). FACS-sorted cells were immediately processed for RNA extraction. Cell cycle length was measured by propidium iodide (PI), which binds to DNA by intercalating between the bases, as previously described (Krishan, 1975). Briefly, cells were trypsinized, resuspended in PBS, and fixed with 70% of ethanol for 40 min on ice. Cells were then centrifuged, resuspended in PBS for 15 min, and then incubated for 1 h at 37°C with 60 µg/ml of PI (Sigma). Cells were collected by centrifuge and resuspended in ice-cold PBS for FACS analysis.

Primary aNPC isolation and culture

Hippocampal NPCs were prepared and expanded as described previously and induction of spontaneous differentiation by growth factor removal was done as previously described (Pons-Espinal *et al*, 2017, 2019); viral-induced neuronal differentiation of aNPCs was done by transduction of a viral construct expressing Ascl1-ERT2 as previously described (Braun *et al*, 2013).

Histology, immunofluorescence, and imaging

Immunofluorescence staining on brain slices was performed on sections covering the entire dorsal hippocampus as previously described (Pons-Espinal *et al*, 2019). Forty micrometer-tick brain sections were generated using a sliding microtome and were stored in a −20°C freezer as floating sections in 48 well plates filled with cryoprotectant solution (glycerol, ethylene glycol, and 0.2 M phosphate buffer, pH 7.4, 1:1:2 by volume).

To detect Ki67 staining, citrate buffer 10 mM pH = 6 treatment for 10 min at 95°C was used. Secondary fluorescent antibodies were diluted 1:1,000 (Goat Alexa 488, 568, and 647 nm, Invitrogen). Confocal stack images of brain slices (40 µm) were obtained with the Confocal A1 Nikon Inverted SFC with 20× objective (Nikon Instruments, Yokohama, Japan). Cell quantification and analysis were performed using NIS-Elements software (Nikon Instruments) and the Cell-counter plugin in Fiji. GFAP intensity fluorescence analysis was done on fluorescence microscopy images acquired by the Confocal A1 Nikon Inverted SFC with 20× objective (Nikon Instruments), with the same parameters for all the sections. Quantification of fluorescence intensity was performed using ImageJ measuring the integrated density of a region of interest (ROI) of 500 µm^2 for each hippocampal slice. Fold change in fluorescence intensity of Mili KD hippocampi compared with control ones has been plotted in the graph. For all the quantification analysis, six sections were analyzed from each animal, and the mean of the measures from consecutive sections was used for that individual. DAB staining was performed as previously reported (Bielefeld *et al*, 2019). Briefly, sections were incubated with peroxidase block (Vectashield) and permeabilized with 0.3% PBS-Triton X (PBS-T) and 0.1% PBS-T. Sections were blocked with 0.1% PBS-T and 5% Normal Goat Serum (NGS), incubated with primary antibodies, and subsequently with the corresponding biotinylated secondary antibodies (1:1,000 Goat anti-rabbit, Invitrogen). Signal amplification was performed using the ABC complex (Vectashield), according to the manufacturer's instructions. Sections were incubated with the solution for DAB reaction (Sigma) and counterstained with Hoechst (1:300), mounted, and coverslipped with Vectashield reagent (VECTOR Labs). ß-galactosidase detection was obtained with the Senescence Cells Histochemical Staining Kit (Sigma-Aldrich), according to the manufacturer's instructions. Briefly, cells were plated on coverslip in proliferating medium. Forty-eight hours after the induction of spontaneous differentiation, cells were washed twice with PBS 1× and incubated with Fixation Buffer 1× for 7 min at RT. Next, cells were rinsed with PBS 1× and incubated with fresh senescence-associated ß-Gal (SA-ß-Gal) stain solution at 37°C (no

© 2022 The Authors

CO_2) for 4 h. Reaction was blocked with PBS 1×, and coverslips were mounted on slides using Vectashield reagent (Vector Labs). Images were obtained using the microscope Nikon Eclipse 80i (Nikon Instruments, Yokohama, Japan) and the percentage of cells expressing ß-galactosidase was quantified over the number of total cells using a Cell-counter plugin in Fiji software. To detect ß-galactosidase *in vivo*, brain slices of 40 μm were collected from perfused animals, and the reaction was carried out in free-floating sections as previously described for cells. Images (DAB and ß-galactosidase) were obtained using the microscope Olympus BX51 equipped with Neurolucida software (MBF Bioscience), and a full brain slice was reconstructed from acquired fields. Immunofluorescence staining on cell cultures was performed as reported (Pons-Espinal *et al*, 2019). To detect BrdU incorporation, cells were pretreated with 2 N HCl for 30 min at 37°C. Cells were mounted in a mounting medium and counterstained with fluorescent nuclear dye DAPI (Invitrogen). Images were obtained using the microscope Nikon Eclipse 80i at 20× or 40× magnification and quantification was performed using a Cell-counter plugin in Fiji. For the quantification analysis, six fields were analyzed from each coverslip, and the mean of the measures was used for that experimental replica.

Mili knockdown (KD) in vitro

Adult neural progenitor cells were infected at MOI = 5 with a lentivirus encoding for a *Mili*-targeted short hairpin (shMILI, plKO.1, Sigma) or for a short hairpin scramble lentivirus (Control, SHC202, Sigma) both decorated with an eGFP reporter. GFP-positive cells were first selected by FACS after three passages and then plated in proliferating or differentiating media, as previously described. We also performed the knockdown using two different synthetic antisense LNA GapmeRs for Mili KD or negative control (as for the *in vivo*: Mili 339512, Control 339516, Qiagen). Cells were transfected with 150 pmol of GapmeRs 50 μM using Lipofectamin stem transfection reagent (STEM00015, Thermo Fisher) according to the manufacturer's protocol and collected 72 h after treatment. GapmeRs uptake and Mili knockdown were assessed by real-time qPCR and Western Blot.

Protein extraction and Western blot

For total protein extraction, adult testes or hippocampus or cell pellets were homogenized in RIPA buffer and the protein concentration was determined using a Bradford Assay kit (Bio-Rad). For blot analysis, equal amounts of protein (30 μg) were run on homemade 10% polyacrylamide gels and transferred on nitrocellulose membranes (GE Healthcare). Membranes were probed with the primary antibodies, followed by HRP-conjugated secondary antibody anti-rabbit or mouse (Invitrogen, A16104, A16072; 1:2,000). LAS 4000 Mini Imaging System (GE Healthcare) was used to digitally acquire chemiluminescence signals, and the band intensities were quantified using Fiji software (Macbiophotonics) (Schindelin *et al*, 2012).

Co-immunoprecipitation of Mili and piRNA

Adult NPCs were suspended in ice-cold lysis buffer supplemented with protease inhibitor (Roche) and RNAse inhibitor (Promega) and lysed with a 30G syringe needle. Equal concentrations of cell lysate were incubated overnight at 4°C in rotation with either IgG (Millipore-Sigma), or the anti-Mili primary antibody (Santa Cruz, sc-377258) and UV cross-linked. Dnase I (Sigma) was added, and the suspensions were incubated with Dynabeads Protein G (Thermo Fisher) to immunoprecipitate the antigen–antibody complex for 2 h at room temperature in rotation. The magnetic beads were captured on a magnetic rack and washed five times each with buffers at increasing salt concentrations. Ten percent of the final wash solution was removed for RNA extraction and subject to real-time qPCR or analysis with the Bioanalyzer RNA chips (Agilent). The remainder of the beads was captured on a magnetic rack, and the protein content was eluted in RIPA buffer supplemented with 150 mM TCEP, incubated on ice for 10 min, and resolved on a 4–12% polyacrylamide gel. Proteins were transferred and processed for Western blotting as above.

RNA extraction and real-time qPCR

Total RNA was extracted from aNPCs (proliferating and differentiating conditions), or DG dissected from adult C57BL/6, Nestin-GFP, or Td-Tomato$^{flox/wt}$ mice with QIAzol protocol (Qiagen) according to the manufacturer's instructions. One microgram of total RNA was treated with DNase I (Sigma) and cDNA was synthesized using iScript cDNA Synthesis kit (Bio-Rad) or with ImProm-II reverse transcriptase (Promega). Real-time qPCR was performed in a duplex with Actin as a reference gene, with QuantiFast SYBR Green PCR Kit (Qiagen) or TaqMan Assay (Thermo Fisher) on ABI-7500 Real-Time PCR System (Applied Biosystems). Expression levels were determined relative to Actin, using the delta-delta Ct method. Primers were designed using NCBI/UCSC Genome Browser and Primer3 software tools and then checked in PrimerBLAST for their specificity to amplify the desired genes. For piRNA real-time qPCR, total RNA enriched in the fraction of small RNAs was extracted using miRNeasy Mini kit (Qiagen) following the manufacturer's instructions from aNPCs, microdissected DG from hippocampi of C57BL6/J or Td-Tomato$^{flox/wt}$ mice. cDNA was obtained using the TaqMan MicroRNA Reverse Transcription Kit (Thermo Fisher) according to the manufacturer's instructions and quantified using the Custom TaqMan Small RNA Assay (Thermo Fisher) on a ABI-7500 Real-Time PCR System (Applied Biosystems). Each sample was normalized to U6 snRNA level (Thermo Fisher). Oligonucleotide sequences are listed in the Table EV1.

RNA library preparation

For small RNA libraries, the quantity and quality of the total RNA isolated from aNPCs/neuroblasts cultures were measured by Nanodrop spectrophotometer (Thermo Fisher) and Experion RNA chips (Bio-Rad). RNA with RNA integrity number (RIN) values ≥ 9.5 were selected for the study. One microgram of high-quality RNA for each sample was used for library preparation according to the Illumina TruSeq small RNA library protocol (Illumina Inc., CA). Briefly, 3′ adapters were ligated to 3′ end of small RNAs using a truncated RNA ligase enzyme followed by 5′ adaptor ligation using an RNA ligase enzyme. Reverse transcription followed by PCR was used to prepare cDNA using primers specific for the 3′ and 5′ adapters. The amplification of those fragments having adapter molecules on both ends was carried out with 13 PCR cycles. The amplified libraries were pooled together and run on a 6% polyacrylamide gel. The 145–160 bp bands (which correspond to inserts of 24–32 nt cDNAs) were extracted and purified using the Wizard® SV Gel and PCR Clean-Up System (Promega). The quality of the library was assessed by the Experion DNA 1 K chips (Bio-Rad). Small RNA sequencing

 © 2022 The Authors

using HiSeq2000 (Illumina Inc., CA) was performed by the IIT genomics facility at the Center for Genomic Science (IIT@SEMM, Milan, Italy). For Long RNA Libraries, quantity and quality of the total RNA extracted from WT/Mili KD aNPCs were measured by Qubit 4 Fluorometer (Thermo Fisher) and Bioanalyzer RNA chips (Agilent). RNA with RIN values ≥ 8 were selected for the study. Thirty nanogram of high-quality RNA for each sample was used for library preparation according to the Illumina Stranded Total RNA Prep, Ligation with Ribo-Zero Plus Kit (20040529, Illumina Inc., CA) using the IDT for Illumina Indexes Set A (20040553). Briefly, after ribosomal RNA depletion, RNA was fragmented, denatured and cDNA synthesized. The 3′ ends were adenylated and anchors ligated. After amplification and clean-up, the quality of the libraries was assessed by the Bioanalyzer DNA chips (Agilent). Paired-End stranded total RNA sequencing on NovaSeq 6000 Sequencing System instrument (Illumina Inc., CA), was performed by the IIT Genomic facility at the Center for Human Technologies, Genoa, Italy. Sequencing was performed bidirectionally, and in duplicate by two flow cell pairs of 100 and 150 base pairs, for a total of six measurements produced from three independent samples for each differentiation time point and genotype.

Small RNA sequencing data processing

Was done essentially as previously published (Ghosheh *et al*, 2016). Briefly, Illumina reads were trimmed to remove the 3′ adapter using Cutadapt, with parameters -m 25 -q 20. Since piRNA size ranges from 26 to 31 bases, all sequences with length ≤ 24 bases were discarded. Reads mapped to known noncoding RNAs (RNAcentral v6.0 snoRNA, UCSC tRNA, miRBase Release 21 miRNA hairpin and mature miRNA annotation, NCBI complete ribosomal DNA unit) were removed from the datasets. The comparison was performed using NCBI BLASTN v2.6.0 with parameters -max_hsps = 1, -max_target_seqs = 1, -perc_identity = 80, mismatches <= 1, qcovhsp >= 90. Reads were aligned on the nonrepeat-masked UCSC release 9 of the mouse genome (MM9) using *bowtie* (Langmead & Salzberg, 2012) v2.2.6 with the sensitive preset option and allowed a maximum of 100 alignments. All the reads that aligned to the genome were retained and used for subsequent analysis. piRNA clusters were identified collapsing overlapped piRNA sequences (piRBase Release 1; Zhang *et al*, 2014) into one cluster (mergeBed with preset options; Quinlan & Hall, 2010). piRNA clusters and all the reads that aligned to the genome were intersected (intersectBed with option -f 1). Intersection files were then parsed using a custom perl script in order to evaluate alignment counts. Differential expression was assessed using DESeq2 (Love *et al*, 2014). piRNA clusters were considered differentially expressed when the adjusted *P*-value was ≤0.05, and down- and up-regulation was established in the range of ≤ −1 to ≥ 1 log2 fold change, respectively. piRNA sequences were then categorized for the putative mRNA transcript targets (for each gene). In order to obtain a count of piRNA target levels, which target individual gene transcripts, for each differentiation time point (DIF0-7), piRNA transcripts were expressed in transcripts per million (TPM) and summed for each target category. Spearman correlation was performed between the levels of the piRNA in the Sh-Scramble (control) genotype and compared with the fold-change level of the putative target genes, which were found to be significantly altered (up and down) in the Sh-Mili-KD genotype. In order to assess the clustering behavior of putative piRNAs, the 5′ termini positions of each cluster-associated putative primary and putative secondary piRNA sequences were analyzed for distance, represented as probability, within a range of 200 nucleotides in the 5′ direction and 200 nucleotides in the 3′ direction of the putative primary piRNAs, as reported previously (Gainetdinov *et al*, 2018). The positional distance between piRNAs for each cluster was sampled iteratively for each assigned piRNA and normalized by the total number of diverse piRNAs associated with each cluster. The distance probability distribution was assayed by the locally weighted smoothing linear regression method (LOWESS), by using the built-in MATLAB "fit" function (MathWorks, Natick, MA), with a span value of 0.1.

mRNA sequencing data processing

Adapter sequences were trimmed using *Cutadapt*, after which a quality control trim was implemented on a sliding window of 25 nucleotides, for 2 base pairs with a minimum quality score of 26, with the *Bowtie* build for the MATLAB bioinformatics suite (MathWorks, Natick, MA). Transcript quantification was performed with the Salmon suite (Patro *et al*, 2017), on the NCBI mouse genome, release 67, obtained from the ENSEMBL FASTA directory. The identified ENSEMBL gene accessions were grouped for the different transcript reads, and the read counts, expressed in TPM, were summed for the annotated transcripts. Outlier reads for each gene transcript level in TPM were detected by the mean absolute deviations method (MAD), where reads with more than 3 scaled MAD distances from the mean were eliminated from statistical analysis. Then, the mean and SD for each gene were used for statistical analysis among the different genotypes by one-way ANOVA with multiple comparisons. *P*-values lower than, or equal to, 0.05 were selected as the threshold of significance, for a minimum count of 4 of 6 samples per gene. Database for Annotation, Visualization and Integrated Discovery (DAVID, https://david.ncifcrf.gov/) (Huang *et al*, 2009), was used to perform Gene ontology (GO) and Kyoto Encyclopedia of Genes and Genomes (KEGG) signaling pathway analysis using whole *Mus musculus* genome as background. Expression heatmap correlation plots were computed by the k-means clustering method, with imputation performed by the nearest-neighbor method by the MATLAB "clustergram" function (MathWorks, Natick, MA, USA), as described previously (Eisen *et al*, 1999).

Periodate oxidation/alkaline Ô-elimination

Periodate oxidation and alkaline ß-elimination were performed as previously described (Balaratnam *et al*, 2018). Briefly, total small RNA fractions were collected from 8 × 10^6 aNPCs (per replica). Two portions, each containing 25 µg of small RNA were dissolved in 87.5 µl 0.06 M borate buffer (pH 8.6). Then, 12.5 µl of nuclease-free water (to control group) or 200 mM sodium periodate (to treatment group) were added to the reaction and samples were incubated for 1 h at room temperature. After the incubation, the reaction was stopped by adding 10 µl of glycerol for another 30 min. For the control group (treated with water), 12.5 µl sodium periodate was incubated with glycerol for 60 min prior to adding to the samples to maintain the same ion strength between the control and treatment. RNA was then precipitated by ethanol precipitation method 1 h at −80°C. Precipitated RNA was dissolved in 100 µl of 0.055 M borate buffer (pH 9.5) and incubated for 60 min at 45°C. RNA was precipitated again as before, washed, and used for TaqMan small RNA

© 2022 The Authors

assay. As an internal control for the assay, a synthetic piRNA sequence (corresponding to one of the most abundant piRNA found in aNPCs bearing homology to piR-cluster-1) either modified with the 3′-end 2′-O-methylation (positive control) or bearing a terminal 2′,3′-hydroxyl group (negative control) were subject to the periodate treatment as above.

Protein synthesis assay

To quantify the protein synthesis rate of cells, we used the Global Protein Synthesis Assay Kit (FACS/Microscopy) and Red Fluorescence kit (Abcam), following the manufacturer's instructions. Briefly, cells in proliferating or differentiating media (DIF7) were treated with Cycloheximide as an inhibitor of protein synthesis, for 30 min at 37°C. Media were replaced with fresh aliquots containing Protein Label (400×) diluted to 1× final concentration and the cells were incubated for additional 30 min at 37°C. Negative control cells were not incubated with the protein label. Samples were analyzed by FACS for red fluorescence generated by *de novo* synthesized protein during click reaction. Translation rate is directly proportional to emitted fluorescence. Cells emitting fluorescence lower than 10^3 were considered negative (P3) and higher than 10^4 were considered positive (P2).

Immunofluorescence, STED nanoscopy, and particle analysis

Confocal and Stimulated Emission Depletion (STED) nanoscopy were performed as previously reported (Vicidomini *et al*, 2018; Diaspro & Bianchini, 2020). aNPCs were plated on glass coverslips 24 h before fixation. Cells were fixed with PFA 4%, permeabilized with PBS-T 0.1%, blocked for 1 h at room temperature with PBS-T 0.1% NGS 5%, and incubated according to the dilution suggested by the manufacturer's instructions with 0.01 µg/ml rabbit polyclonal antibody against the N terminus of RPL26 (Abcam) for 1 h at room temperature. Cells were washed extensively and incubated with the secondary antibody goat anti-rabbit ATTO-647N (0.8 µg/ml; Sigma) for 45 min. Nuclei were stained while mounting the coverslip with DAPI-Prolong antifade (Invitrogen). Confocal and STED images were acquired at 23°C with a modified TCS SP5 STED-CW gated and operated with its own imaging software, LAS X (Leica Microsystems, Mannheim, Germany). The microscope has been customized with a second pulsed STED laser line at 775 nm. The beam originates from a Onefive Katana HP 8 (NKT, Birkerød, Denmark) and passes through a vortex phase plate (RPC photonics, Rochester, NY, USA) before entering the microscope through the IR port. The depletion laser pulses are electronically synchronized with the Leica's supercontinuum pulsed and visible excitation laser. The ATTO-647N fluorescence was excited at 633 nm, and the fluorescence depletion was performed at 775 nm. The maximal focal power of the STED beam was 200 mW at 80 MHz. Both beams were focused into the 1.4 NA objective lens (HCX PL APO 100× 1.40 NA Oil STED Orange; Leica). Fluorescence was collected by the same lens, filtered with a 775 nm notch filter, and imaged in the spectral range 660–710 nm by the hybrid detector with a time gating of 1 ns. All the images have a 14 nm pixel size and 37-µs pixel dwell time. The analysis of polysome clusters in aNPC lineages was performed on more than 20 images likewise different cells. Image analysis was performed using the Fiji software.

In silico piRNA targets prediction

For piRNA targets analysis, we divided the sequencing data into one set of 100 piRNA clusters enriched in proliferating aNPCs (DIF0) and a second set of 198 clusters specifically expressed at DIF4/7 stage. The Differential Expression analysis for piRNAs mapping on repeat elements (REs) in DIF4 and DIF7 compared with DIF0 was done using EdgeR software package (Robinson & Oshlack, 2010). Identification of piRNA targets was divided into: piRNAs mapping on REs only/piRNAs mapping on GENCODE elements/piRNAs mapping on REs within GENCODE elements/unannotated piRNAs/piRNAs clusters. Gene Ontology analysis for piRNAs mapping on GENCODE protein-coding genes (but NOT mapping on REs) has been done with the R package GOfuncR (https://bioconductor.org/packages/release/bioc/html/GOfuncR.html).

Quantification and statistical analysis

Data are presented as mean ± SEM and were analyzed using Prism 6 (GraphPad). Statistical significance was assessed with a two-tailed unpaired *t*-test for two experimental groups. For experiments with three or more groups, one-way ANOVA with the Bonferroni's multiple comparison test was used. Results were considered significant when $P < 0.05$. The number of samples (n) in each group is reported in the figure legend. Exact *P*-values of the experiments are shown in Table 1.

Table 1. Exact *P*-values of the experiments presented in the paper.

Figure	Comparison	*P*-value	Statistical test
1B	DIF0/DIF7	$^{**}P = 0.0022$	One-way ANOVA, *post-hoc* Bonferroni
	DIF0/DIF14	$^{***}P = 0.0001$	
	DIF4/DIF7	$^{***}P = 0.0003$	
	DIF4/DIF14	$^{****}P < 0.0001$	
1C	Testis/Hippocampus	$^{***}P = 0.0004$	One-way ANOVA, *post-hoc* Bonferroni
	Testis/aNPCs	$^{***}P = 0.0005$	
1D	Testis/Hippocampus	$^{***}P = 0.0002$	One-way ANOVA, *post-hoc* Bonferroni
	Testis/aNPCs	$^{**}P = 0.0013$	
1E	Neurons/aNPCs	$^{***}P = 0.0008$	Two-tailed Student's *t*-test
1G	Td^-/Td^+ 10 dpi	$^{*}P = 0.0224$	One-way ANOVA, *post-hoc* Bonferroni
	Td^+ 30/10 dpi	$^{*}P = 0.0355$	

 © 2022 The Authors

Table 1 (continued)

Figure	Comparison	*P*-value	Statistical test
2E	mRNA Ctl/Mili KD	*$P = 0.014$	Two-tailed Student's *t*-test
	WB Ctl/Mili KD	**$P = 0.0015$	
2F	piCS1 Ctl/Mili KD	***$P = 0.0004$	Two-tailed Student's *t*-test
	piCS2 Ctl/Mili KD	***$P = 0.0005$	
	piCS3 Ctl/Mili KD	***$P = 0.0002$	
	piCS4 Ctl/Mili KD	****$P < 0.0001$	
2I	piCS2 anti-Piwil2/IgG	$^{ns}P = 0.1018$	Two-tailed Student's *t*-test
	piCS3 anti-Piwil2/IgG	*$P = 0.0423$	Two-tailed Student's *t*-test
	piCS4 anti-Piwil2/IgG	*$P = 0.0142$	Two-tailed Student's *t*-test
	piCS5 anti-Piwil2/IgG	**$P = 0.0097$	Two-tailed Student's *t*-test
3B	piCS1 Td^-/Td^+	*$P = 0.0164$	Two-tailed Student's *t*-test
	piCS2 Td^-/Td^+	**$P = 0.0028$	
	piCS3 Td^-/Td^+	*$P = 0.0184$	
	piCS4 Td^-/Td^+	*$P = 0.0278$	
4C	mRNA Ctl/Mili KD	*$P = 0.0152$	Two-tailed Student's *t*-test
	WB Ctl/Mili KD	**$P = 0.0083$	
4E	GFAP Ctl/Mili KD	**$P = 0.0061$	Two-tailed Student's *t*-test
	mRNA Ctl/Mili KD	****$P < 0.0001$	
4F	NeuN Ctl/Mili KD	**$P = 0.0013$	Two-tailed Student's *t*-test
	GFAP Ctl/Mili KD	**$P = 0.0077$	
4G	C3 Ctl/Mili KD	*$P = 0.0124$	Two-tailed Student's *t*-test
	Serpin Ctl/Mili KD	**$P = 0.0068$	
	Cxcl10 Ctl/Mili KD	**$P = 0.0013$	
4H	Mili Ctl/KA	**$P = 0.0028$	Two-tailed Student's *t*-test
	piCS1 Ctl/KA	***$P = 0.0002$	
5B	β-Gal Ctl/Mili KD	****$P < 0.0001$	Two-tailed Student's *t*-test
5C	BrdU Ki67 Ctl/Mili KD	****$P < 0.0001$	Two-tailed Student's *t*-test
5D	G0-G1 Ctl/Mili KD	*$P = 0.0186$	Two-tailed Student's *t*-test
	S Ctl/Mili KD	**$P = 0.0056$	
5E	Cyclin A Ctl/Mili KD	*$P = 0.0132$	Two-tailed Student's *t*-test
	Cyclin D1 Ctl/Mili KD	***$P = 0.0003$	
	Cyclin E Ctl/Mili KD	*$P = 0.0266$	
5F	Btg1 Ctl/Mili KD	**$P = 0.0068$	Two-tailed Student's *t*-test
	Btg2 Ctl/Mili KD	*$P = 0.0174$	
	Btg3 Ctl/Mili KD	**$P = 0.0068$	
	Ccnd3 Ctl/Mili KD	*$P = 0.0279$	
	Ppp1ca Ctl/Mili KD	***$P = 0.0005$	
5H	Mili young/old	***$P = 0.0003$	Two-tailed Student's *t*-test
6C	SINE DIF0 Ctl/Mili KD	**$P = 0.0079$	One-way ANOVA, *post-hoc* Bonferroni
	SINE DIF4 Ctl/Mili KD	*$P = 0.0405$	
	SINE DIF7 Ctl/Mili KD	**$P = 0.0059$	
	rRNA DIF0 Ctl/Mili KD	**$P = 0.0074$	
	rRNA DIF4 Ctl/Mili KD	***$P = 0.0002$	
6D	LINE1 DIF7 Ctl/Mili KD	*$P = 0.00472$	Two-tailed Student's *t*-test
6G	mRNA Target Mili KD/CtlmRNA Nontarget Mili KD/Ctl	$P = 3.21E-260$	Two-sample Kolmgorov-Smirnov test

© 2022 The Authors

Table 1 (continued)

Figure	Comparison	*P*-value	Statistical test
EV1A	mRNA Ctl/GpM1	*$P = 0.0133$	Two-tailed Student's *t*-test
	mRNA Ctl/GpM3	*$P = 0.038$	
EV1B	WB Ctl/GpM1	***$P = 0.001$	
	WB Ctl/GpM3	*$P = 0.014$	
EV1C	piCS Ctl/GpM1/GpM3	****$P < 0.0001$	One-way ANOVA, *post-hoc* Bonferroni
EV3B	mRNA Ctl/Mili KD	**$P = 0.098$	Two-tailed Student's *t*-test
	WB Ctl/Mili KD	****$P < 0.0001$	
EV3C	mRNA Ctl/Mili KD	*$P = 0.0477$	Two-tailed Student's *t*-test
	WB Ctl/Mili KD	*$P = 0.017$	
EV3D	GFAP Ctl/Mili KD	**$P = 0.0039$	Two-tailed Student's *t*-test
EV3E	NeuN Ctl/Mili KD	**$P = 0.012$	Two-tailed Student's *t*-test
	GFAP Ctl/Mili KD	****$P < 0.0001$	
EV4B	Bcl2 Ctl/Mili KD	**$P = 0.098$	Two-tailed Student's *t*-test
EV5C	DIF7 Ctl/Mili KD	*$P = 0.0338$	One-way ANOVA, *post-hoc* Bonferroni

Data availability

Mouse Small RNA sequencing data have been deposited in the European Nucleotide Archive (ENA) under the accession: PRJEB40241 (https://www.ebi.ac.uk/ena/browser/view/PRJEB40241?show=reads) and the long RNA sequencing data in the Gene Expression Omnibus (GEO) database under the accession GSE182848 (https://www.ncbi.nlm.nih.gov/geo/query/acc.cgi?acc=GSE182848); human datasets (De Rie *et al*, 2017) are available through RIKEN FANTOM5.

Expanded View for this article is available online.

Acknowledgements

We are grateful to G. Hannon (Cambridge, UK), H. Siomi (Keyo University Japan), and Z. Mourelatos (University of Pennsylvania, USA) for providing Mili and/or Miwi antibodies; G. Enikolopov (Stony Brook University NY, USA) for the *Nestin*-GFP mouse and to D. O'Carroll (University of Edinburgh, UK) for the *Mili* null mice. We thank IIT technical staff (S. Bianchi; M. Pesce; E. Albanesi, M. Morini, D. Vozzi) for excellent assistance; L. Pandolfini (IIT-CHT) for advice in the protocol for piRNA resistance to periodate and M. Veronesi and F. Saccoliti (IIT-CCT) for advice on protein chemistry. We are grateful to N. Tirelli Director of the Open University Affiliated Research Centre at IIT (ARC@IIT), part of the Open University, Milton Keynes MK7 6AA, United Kingdom, for advice and support to KT during his training as a PhD student. This study was funded by Fondazione Istituto Italiano di Tecnologia; and partly by Fondazione Cariplo Grant #2015-0590; AIRC-IG 2017 # 20106; PNRR—CENTRO NAZIONALE 3—"Sviluppo di terapia genica e farmaci con tecnologia a RNA". CPF was funded by an ERA-NET-NEURON EJTC 2016 grant and by The Netherlands organization for scientific research (NWO). We apologize to those colleagues whose work could not be cited due to space limitations.

Author contributions

Caterina Gasperini: Conceptualization; data curation; formal analysis; investigation; visualization; methodology; writing – original draft; writing – review and editing. **Kiril Tuntevski:** Data curation; formal analysis; investigation; visualization; methodology; writing – review and editing. **Silvia Beatini:** Data curation; formal analysis; investigation; visualization; methodology; writing – review and editing. **Roberta Pelizzoli:** Data curation; formal analysis; investigation; methodology. **Amanda Lo Van:** Data curation; formal analysis; visualization. **Damiano Mangoni:** Data curation; formal analysis; investigation; visualization; methodology; writing – review and editing. **Rosa M Cossu:** Data curation; formal analysis; investigation; visualization; methodology; writing – review and editing. **Giovanni Pascarella:** Data curation; software; formal analysis; investigation; visualization; methodology; writing – review and editing. **Paolo Bianchini:** Formal analysis; investigation; visualization; methodology; writing – review and editing. **Pascal Bielefeld:** Formal analysis; investigation; methodology; writing – review and editing. **Margherita Scarpato:** Formal analysis; methodology. **Meritxell Pons-Espinal:** Supervision; methodology; writing – review and editing. **Remo Sanges:** Software; formal analysis; supervision; methodology; writing – review and editing. **Alberto Diaspro:** Supervision; funding acquisition; methodology; writing – review and editing. **Carlos P Fitzsimons:** Formal analysis; supervision; funding acquisition; methodology; writing – review and editing. **Piero Carninci:** Supervision; funding acquisition; methodology; writing – review and editing. **Stefano Gustincich:** Supervision; funding acquisition; methodology; writing – review and editing. **Davide De Pietri Tonelli:** Conceptualization; data curation; formal analysis; supervision; funding acquisition; investigation; visualization; methodology; writing – original draft; project administration; writing – review and editing.

Disclosure and competing interests statement

The authors declare that they have no conflict of interest.

References

Adusumilli VS, Walker TL, Overall RW, Klatt GM, Zeidan SA, Zocher S, Kirova DG, Ntitsias K, Fischer TJ, Sykes AM *et al* (2021) ROS dynamics delineate functional states of hippocampal neural stem cells and link to their activity-dependent exit from quiescence. *Cell Stem Cell* 28: 300–314

Ahlenius H, Visan V, Kokaia M, Lindvall O, Kokaia Z (2009) Neural stem and progenitor cells retain their potential for proliferation and differentiation

 © 2022 The Authors

into functional neurons despite lower number in aged brain. *J Neurosci* 29: 4408 – 4419

Altman J (1962) Are new neurons formed in the brains of adult mammals? *Science* 135: 1127 – 1128

Aravin A, Gaidatzis D, Pfeffer S, Lagos-Quintana M, Landgraf P, Iovino N, Morris P, Brownstein MJ, Kuramochi-Miyagawa S, Nakano T *et al* (2006) A novel class of small RNAs bind to MILI protein in mouse testes. *Nature* 442: 203 – 207

Babcock KR, Page JS, Fallon JR, Webb AE (2021) Adult hippocampal neurogenesis in aging and Alzheimer's disease. *Stem Cell Reports* 16: 1 – 13

Balaratnam S, West N, Basu S (2018) A piRNA utilizes HILI and HIWI2 mediated pathway to down-regulate ferritin heavy chain 1 mRNA in human somatic cells. *Nucleic Acids Res* 46: 10635 – 10648

Bielefeld P, Sierra A, Encinas JM, Maletic-Savatic M, Anderson A, Fitzsimons CP (2017) A standardized protocol for stereotaxic intrahippocampal administration of kainic acid combined with electroencephalographic seizure monitoring in mice. *Front Neurosci* 11: 1 – 9

Bielefeld P, Schouten M, Meijer GM, Breuk MJ, Geijtenbeek K, Karayel S, Tiaglik A, Vuuregge AH, Willems RAL, Witkamp D *et al* (2019) Co-administration of anti microRNA-124 and -137 oligonucleotides prevents hippocampal neural stem cell loss upon non-convulsive seizures. *Front Mol Neurosci* 12: 1 – 13

Bonaguidi MA, Wheeler MA, Shapiro JS, Stadel RP, Sun GJ, Ming GL, Song H (2011) *In vivo* clonal analysis reveals self-renewing and multipotent adult neural stem cell characteristics. *Cell* 145: 1142 – 1155

Braun SMG, Machado RAC, Jessberger S (2013) Temporal control of retroviral transgene expression in newborn cells in the adult brain. *Stem Cell Reports* 1: 114 – 122

Clarke LE, Liddelow SA, Chakraborty C, Münch AE, Heiman M, Barres BA (2018) Normal aging induces A1-like astrocyte reactivity. *Proc Natl Acad Sci USA* 115: E1896 – E1905

Coufal NG, Garcia-Perez JL, Peng GE, Yeo GW, Mu Y, Lovci MT, Morell M, O'Shea KS, Moran JV, Gage FH (2009) L1 retrotransposition in human neural progenitor cells. *Nature* 460: 1127 – 1131

Czech B, Munafò M, Ciabrelli F, Eastwood EL, Fabry MH, Kneuss E, Hannon GJ (2018) piRNA-guided genome defense: from biogenesis to silencing. *Annu Rev Genet* 52: 131 – 157

De Luca L, Trino S, Laurenzana I, Simeon V, Calice G, Raimondo S, Podestà M, Santodirocco M, Di Mauro L, La Rocca F *et al* (2016) MiRNAs and piRNAs from bone marrow mesenchymal stem cell extracellular vesicles induce cell survival and inhibit cell differentiation of cord blood hematopoietic stem cells: a new insight in transplantation. *Oncotarget* 7: 6676 – 6692

De Rie D, Abugessaisa I, Alam T, Arner E, Arner P, Ashoor H, Åström G, Babina M, Bertin N, Burroughs AM *et al* (2017) An integrated expression atlas of miRNAs and their promoters in human and mouse. *Nat Biotechnol* 35: 872 – 878

Di Giacomo M, Comazzetto S, Saini H, DeFazio S, Carrieri C, Morgan M, Vasiliauskaite L, Benes V, Enright AJ, O'Carroll D (2013) Multiple epigenetic mechanisms and the piRNA pathway rnforce LINE1 dilencing during adult spermatogenesis. *Mol Cell* 50: 601 – 608

Diaspro A, Bianchini P (2020) Optical nanoscopy. *Riv Nuovo Cim* 43: 385 – 455

Ding D, Liu J, Dong K, Midic U, Hess RA, Xie H, Demireva EY, Chen C (2017) PNLDC1 is essential for piRNA 3′ end trimming and transposon silencing during spermatogenesis in mice. *Nat Commun* 8: 2 – 11

Doetsch F, Caille I, Lim DA, Garcia-Verdugo JM, Alvarez-Buylla A (1999) Subventricular zone astrocytes are neural stem cells in the adult mammalian brain. *Cell* 97: 703 – 716

Eisen MB, Spellman PT, Brown PO, Botstein D (1999) Cluster analysis and display of genome-wide expression patterns. *Proc Natl Acad Sci USA* 95: 12930 – 12933

Encinas JM, Michurina TV, Peunova N, Park JH, Tordo J, Peterson DA, Fishell G, Koulakov A, Enikolopov G (2011) Division-coupled astrocytic differentiation and age-related depletion of neural stem cells in the adult hippocampus. *Cell Stem Cell* 8: 566 – 579

Escartin C, Galea E, Lakatos A, O'Callaghan JP, Petzold GC, Serrano-Pozo A, Steinhäuser C, Volterra A, Carmignoto G, Agarwal A *et al* (2021) Reactive astrocyte nomenclature, definitions, and future directions. *Nat Neurosci* 24: 312 – 325

Gainetdinov I, Colpan C, Arif A, Cecchini K, Zamore PD (2018) A single mechanism of biogenesis, initiated and directed by PIWI proteins, explains piRNA production in most animals. *Mol Cell* 71: 775 – 790

Ghosheh Y, Seridi L, Ryu T, Takahashi H, Orlando V, Carninci P, Ravasi T (2016) Characterization of piRNAs across postnatal development in mouse brain. *Sci Rep* 6: 1 – 7

Girard A, Sachidanandam R, Hannon GJ, Carmell MA (2006) A germline-specific class of small RNAs binds mammalian Piwi proteins. *Nature* 442: 199 – 202

Huang DW, Sherman BT, Lempicki RA (2009) Systematic and integrative analysis of large gene lists using DAVID bioinformatics resources. *Nat Protoc* 4: 44 – 57

Jain G, Stuendl A, Rao P, Berulava T, Pena Centeno T, Kaurani L, Burkhardt S, Delalle I, Kornhuber J, Hüll M *et al* (2019) A combined miRNA–piRNA signature to detect Alzheimer's disease. *Transl Psychiatry* 9: 250

Jin WN, Shi K, He W, Sun JH, Van Kaer L, Shi FD, Liu Q (2021) Neuroblast senescence in the aged brain augments natural killer cell cytotoxicity leading to impaired neurogenesis and cognition. *Nat Neurosci* 24: 61 – 73

Keam SP, Young PE, McCorkindale AL, Dang THY, Clancy JL, Humphreys DT, Preiss T, Hutvagner G, Martin DIK, Cropley JE *et al* (2014) The human Piwi protein Hiwi2 associates with tRNA-derived piRNAs in somatic cells. *Nucleic Acids Res* 42: 8984 – 8995

Kirino Y, Mourelatos Z (2007) Mouse Piwi-interacting RNAs are 2′-O-methylated at their 3′ termini. *Nat Struct Mol Biol* 14: 347 – 348

Krishan A (1975) Rapid flow cytofluorometric analysis of mammalian cell cycle by propidium iodide staining. *J Cell Biol* 66: 188 – 193

Langmead B, Salzberg SL (2012) Fast gapped-read alignment with Bowtie 2. *Nat Methods* 9: 357 – 359

Lee JH, Schütte D, Wulf G, Füzesi L, Radzun HJ, Schweyer S, Engel W, Nayernia K (2006) Stem-cell protein Piwil2 is widely expressed in tumors and inhibits apoptosis through activation of Stat3/Bcl-XL pathway. *Hum Mol Genet* 15: 201 – 211

Lee EJ, Banerjee S, Zhou H, Jammalamadaka A, Arcila M, Manjunath BS, Kosik KS (2011) Identification of piRNAs in the central nervous system. *RNA* 17: 1090 – 1099

Leighton LJ, Wei W, Marshall PR, Ratnu VS, Li X, Zajaczkowski EL, Spadaro PA, Khandelwal N, Kumar A, Bredy TW (2019) Disrupting the hippocampal Piwi pathway enhances contextual fear memory in mice. *Neurobiol Learn Mem* 161: 202 – 209

Liddelow SA, Guttenplan KA, Clarke LE, Bennett FC, Bohlen CJ, Schirmer L, Bennett ML, Münch AE, Chung W-S, Peterson TC *et al* (2017) Neurotoxic reactive astrocytes are induced by activated microglia. *Nature* 541: 481 – 487

Liu GY, Sabatini DM (2020) mTOR at the nexus of nutrition, growth, ageing and disease. *Nat Rev Mol Cell Biol* 21: 183 – 203

Love MI, Huber W, Anders S (2014) Moderated estimation of fold change and dispersion for RNA-seq data with DESeq2. *Genome Biol* 15: 1 – 21

© 2022 The Authors

Madisen L, Zwingman TA, Sunkin SM, Oh SW, Zariwala HA, Gu H, Ng LL, Palmiter RD, Hawrylycz MJ, Jones AR *et al* (2010) A robust and high-throughput Cre reporting and characterization system for the whole mouse brain. *Nat Neurosci* 13: 133 – 140

Martín-Suárez S, Valero J, Muro-García T, Encinas JM (2019) Phenotypical and functional heterogeneity of neural stem cells in the aged hippocampus. *Aging Cell* 18: 1 – 14

Mignone JL, Kukekov V, Chiang AS, Steindler D, Enikolopov G (2004) Neural stem and progenitor cells in nestin-GFP transgenic mice. *J Comp Neurol* 469: 311 – 324

Mills EW, Green R (2017) Ribosomopathies: there's strength in numbers. *Science* 358: 1 – 8

Muotri AR, Chu VT, Marchetto MCN, Deng W, Moran JV, Gage FH (2005) Somatic mosaicism in neuronal precursor cells mediated by L1 retrotransposition. *Nature* 435: 903 – 910

Nandi S, Chandramohan D, Fioriti L, Melnick AM, Hébert JM, Mason CE, Rajasethupathy P, Kandel ER (2016) Roles for small noncoding RNAs in silencing of retrotransposons in the mammalian brain. *Proc Natl Acad Sci USA* 113: 12697 – 12707

Nolde MJ, Cheng EC, Guo S, Lin H (2013) Piwi genes are dispensable for normal hematopoiesis in mice. *PLoS One* 8: 1 – 8

Ozata DM, Gainetdinov I, Zoch A, O'Carroll D, Zamore PD (2019) PIWI-interacting RNAs: small RNAs with big functions. *Nat Rev Genet* 20: 89 – 108

Page NF, Gandal MJ, Estes ML, Cameron S, Buth J, Parhami S, Ramaswami G, Murray K, Amaral DG, Van de Water JA *et al* (2021) Alterations in retrotransposition, synaptic connectivity, and myelination implicated by transcriptomic changes following maternal immune activation in nonhuman primates. *Biol Psychiatry* 89: 896 – 910

Patro R, Duggal G, Love MI, Irizarry RA, Kingsford C (2017) Salmon provides fast and bias-aware quantification of transcript expression. *Nat Methods* 14: 417 – 419

Penning A, Tosoni G, Abiega O, Bielefeld P, Gasperini C, De Pietri Tonelli D, Fitzsimons CP, Salta E (2022) Adult neural stem cell regulation by small non-coding RNAs: physiological significance and pathological implications. *Front Cell Neurosci* 15: 1 – 20

Perera BPU, Tsai ZTY, Colwell ML, Jones TR, Goodrich JM, Wang K, Sartor MA, Faulk C, Dolinoy DC (2019) Somatic expression of piRNA and associated machinery in the mouse identifies short, tissue-specific piRNA. *Epigenetics* 14: 504 – 521

Pons-Espinal M, de Luca E, Marzi MJ, Beckervordersandforth R, Armirotti A, Nicassio F, Fabel K, Kempermann G, De Pietri Tonelli D (2017) Synergic functions of miRNAs determine neuronal fate of adult neural stem cells. *Stem Cell Reports* 8: 1046 – 1061

Pons-Espinal M, Gasperini C, Marzi MJ, Braccia C, Armirotti A, Pötzsch A, Walker TL, Fabel K, Nicassio F, Kempermann G *et al* (2019) MiR-135a-5p is critical for exercise-induced adult neurogenesis. *Stem Cell Reports* 12: 1298 – 1312

Quinlan AR, Hall IM (2010) BEDTools: a flexible suite of utilities for comparing genomic features. *Bioinformatics* 26: 841 – 842

Robinson MD, Oshlack A (2010) A scaling normalization method for differential expression analysis of RNA-seq data. *Genome Biol* 11: R25

Rojas-Ríos P, Simonelig M (2018) piRNAs and PIWI proteins: regulators of gene expression in development and stem cells. *Development* 145: 1 – 13

Schindelin J, Arganda-Carreras I, Frise E, Kaynig V, Longair M, Pietzsch T, Preibisch S, Rueden C, Saalfeld S, Schmid B *et al* (2012) Fiji: an open-source platform for biological-image analysis. *Nat Methods* 9: 676 – 682

Schouten M, Bielefeld P, Garcia-Corzo L, Passchier EMJ, Gradari S, Jungenitz T, Pons-Espinal M, Gebara E, Martín-Suárez S, Lucassen PJ *et al* (2020) Circadian glucocorticoid oscillations preserve a population of adult hippocampal neural stem cells in the aging brain. *Mol Psychiatry* 25: 1382 – 1405

Sharma AK, Nelson MC, Brandt JE, Wessman M, Mahmud N, Weller KP, Hoffman R (2001) Human CD34^{+} stem cells express the hiwi gene, a human homologue of the Drosophila gene Piwi. *Blood* 97: 426 – 434

Sierra A, Martín-Suárez S, Valcárcel-Martín R, Pascual-Brazo J, Aelvoet SA, Abiega O, Deudero JJ, Brewster AL, Bernales I, Anderson AE *et al* (2015) Neuronal hyperactivity accelerates depletion of neural stem cells and impairs hippocampal neurogenesis. *Cell Stem Cell* 16: 488 – 503

Toda T, Parylak SL, Linker SB, Gage FH (2019) The role of adult hippocampal neurogenesis in brain health and disease. *Mol Psychiatry* 24: 67 – 87

Torres AG, Reina O, Attolini CSO, De Pouplana LR (2019) Differential expression of human tRNA genes drives the abundance of tRNA-derived fragments. *Proc Natl Acad Sci USA* 116: 8451 – 8456

Upton KR, Gerhardt DJ, Jesuadian JS, Richardson SR, Sánchez-Luque FJ, Bodea GO, Ewing AD, Salvador-Palomeque C, Van Der Knaap MS, Brennan PM *et al* (2015) Ubiquitous L1 mosaicism in hippocampal neurons. *Cell* 161: 228 – 239

Vicidomini G, Bianchini P, Diaspro A (2018) STED super-resolved microscopy. *Nat Methods* 15: 173 – 182

Viero G, Lunelli L, Passerini A, Bianchini P, Gilbert RJ, Bernabò P, Tebaldi T, Diaspro A, Pederzolli C, Quattrone A (2015) Three distinct ribosome assemblies modulated by translation are the building blocks of polysomes. *J Cell Biol* 208: 581 – 596

Wakisaka KT (2019) The dawn of pirna research in various neuronal disorders. *Front Biosci* 24: 1440 – 1451

Walker TL, Kempermann G (2014) One mouse, two cultures: isolation and culture of adult neural stem cells from the two neurogenic zones of individual mice. *J Vis Exp* 84: 1 – 9

Yu C-W (1992) The assessment of cellular proliferation by immunohistochemistry: a review of currently available methods and their applications. *Histochem J* 24: 121 – 131

Zhang P, Si X, Skogerbø G, Wang J, Cui D, Li Y, Sun X, Liu L, Sun B, Chen R *et al* (2014) piRBase: a web resource assisting piRNA functional study. *Database* 2014: 1 – 7

Zhao P, Yao M, Chang S, Gou L, Liu M, Qiu Z (2015) Novel function of PIWIL1 in neuronal polarization and migration via regulation of microtubule-associated proteins. *Mol Brain* 8: 1 – 12

License: This is an open access article under the terms of the Creative Commons Attribution License, which permits use, distribution and reproduction in any medium, provided the original work is properly cited.

Expanded View Figures

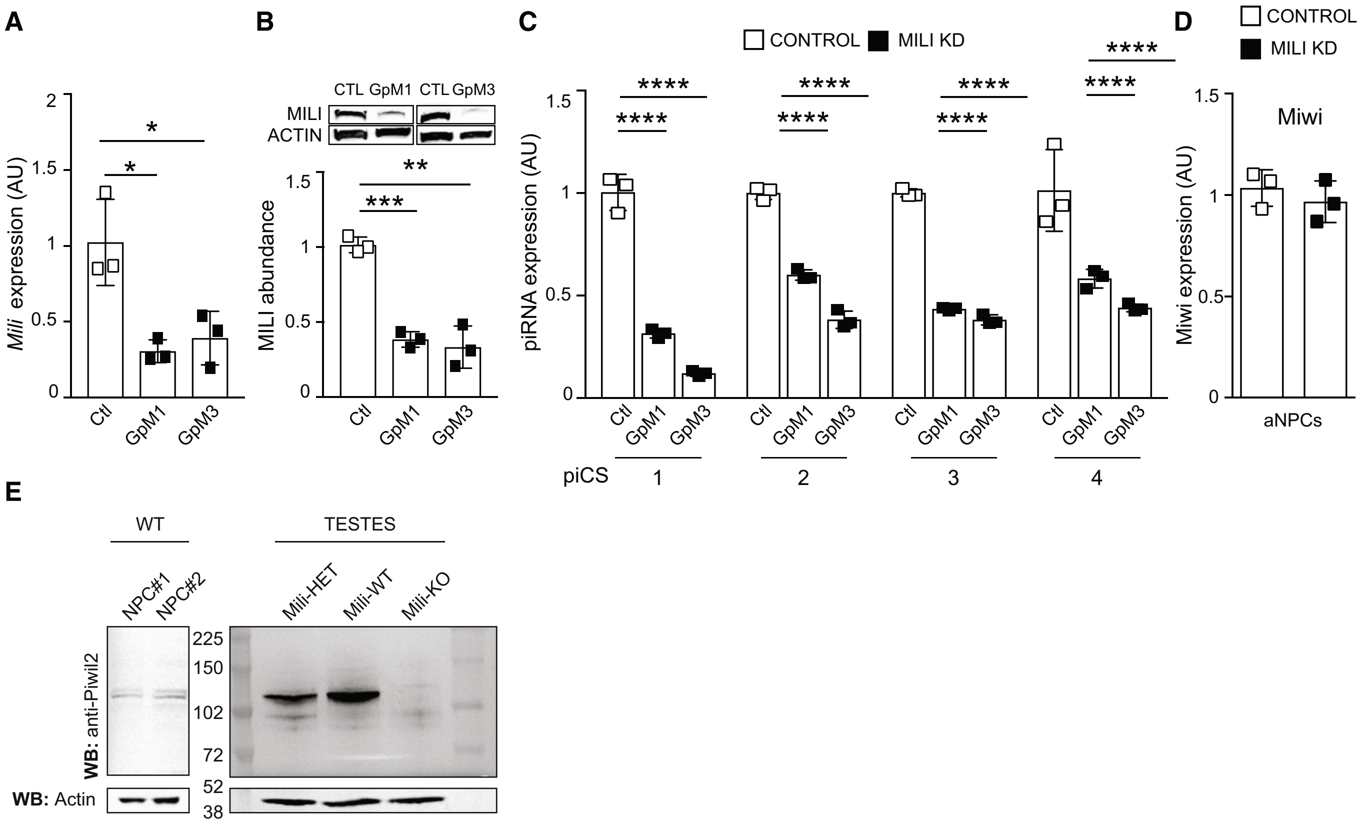

Figure EV1. Mili KD depletes piRNAs and does not affect Miwi. Relative to Fig 2.

A, B *Mili* mRNA expression (A); western blot (B, inset) and quantification of Mili protein abundance (B, bar graph) in lysates from aNPCs upon transfection with control GapmeR (Ctl) or two different GapmeRs (GpM1, GpM3) targeting *Mili*.

C Expression of transcripts of piRNA-cluster consensus sequences (piCS) in control and Mili KD (two independent GapmeRs) aNPCs.

D Relative expression of *Miwi* transcript in undifferentiated aNPCs transduced with viruses transcribing a Scrambled short hairpin (Control) and short hairpin against *Mili* (Mili KD).

E Western blot of Mili protein in lysates from aNPCs ($n = 2$ biological replicates) and from testes of mice wildtype (WT), heterozygous (HET) or knockout (KO) for the *Mili* gene.

Data information: data are expressed as mean ± SEM, $n = 3$ biological replicates (A–C). *$P < 0.05$, **$P < 0.01$, ***$P < 0.001$, ****$P < 0.0001$, as assessed by the two-tailed Student's *t*-test (A, B) or One-way ANOVA, *post-hoc* Bonferroni (C).
Source data are available online for this figure.

© 2022 The Authors

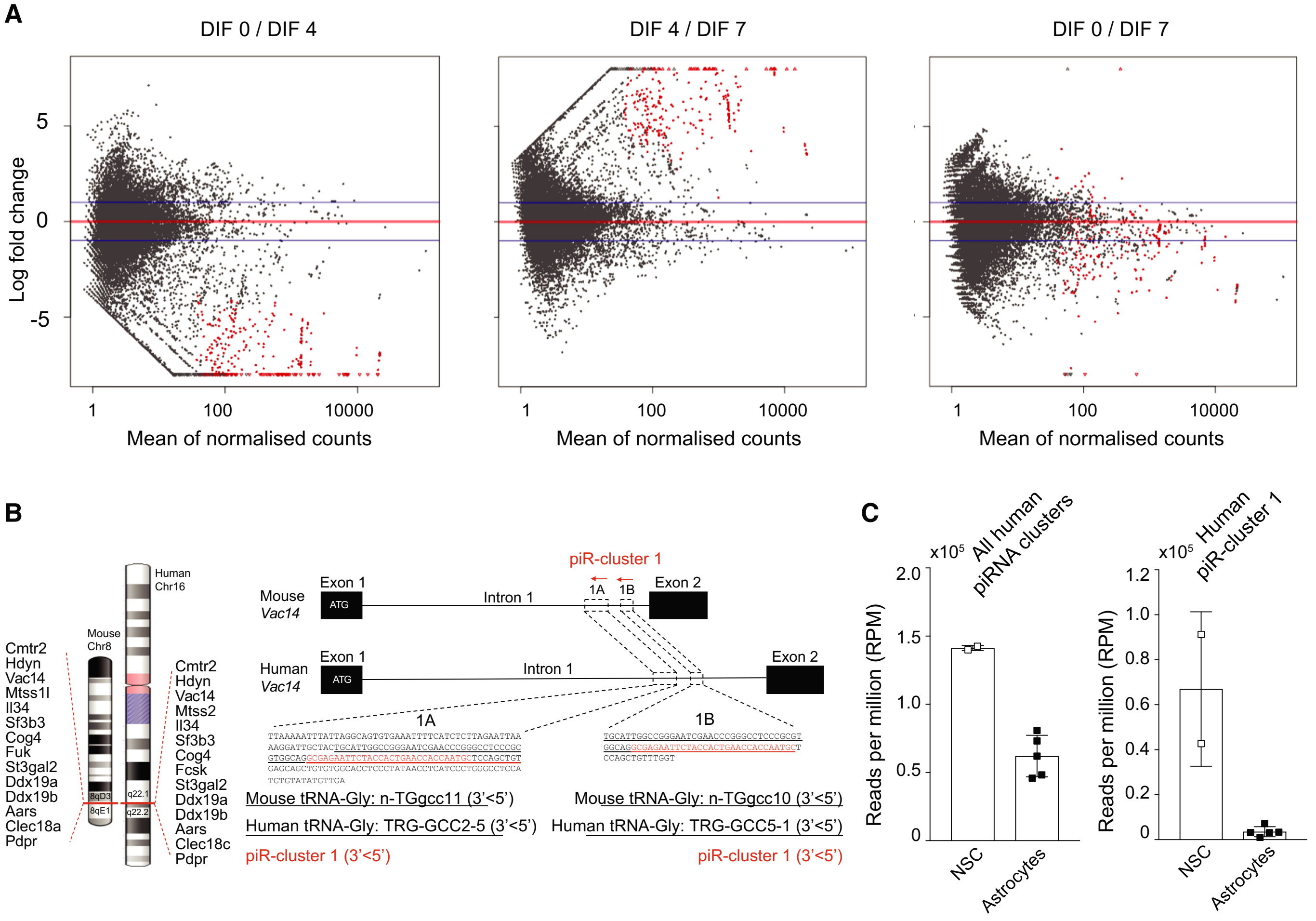

Figure EV2. Expression of piRNAs in mouse and human NSC and progeny. Relative to Fig 3.

A Pairwise comparison of 298 piRNA clusters differentially expressed in undifferentiated aNPCs (DIF0) or neuroblasts upon viral-induced neurogenesis (DIF4-7). $n = 2$ biological replicates.

B Chromosomal location of piR-cluster 1 in mouse and human; (Right) genomic location and sequences (underlined red text) of piR-cluster 1 corresponding to tRNAGly genes (underlined black text).

C Expression of piRNA clusters (left) and piR-cluster 1 (right) in human NSC and astrocytes. $n = 2$ biological replicates of human NSCs; $n = 5$ biological replicates of human astrocytes.

Data information: data in C are expressed as mean ± SEM.

 © 2022 The Authors

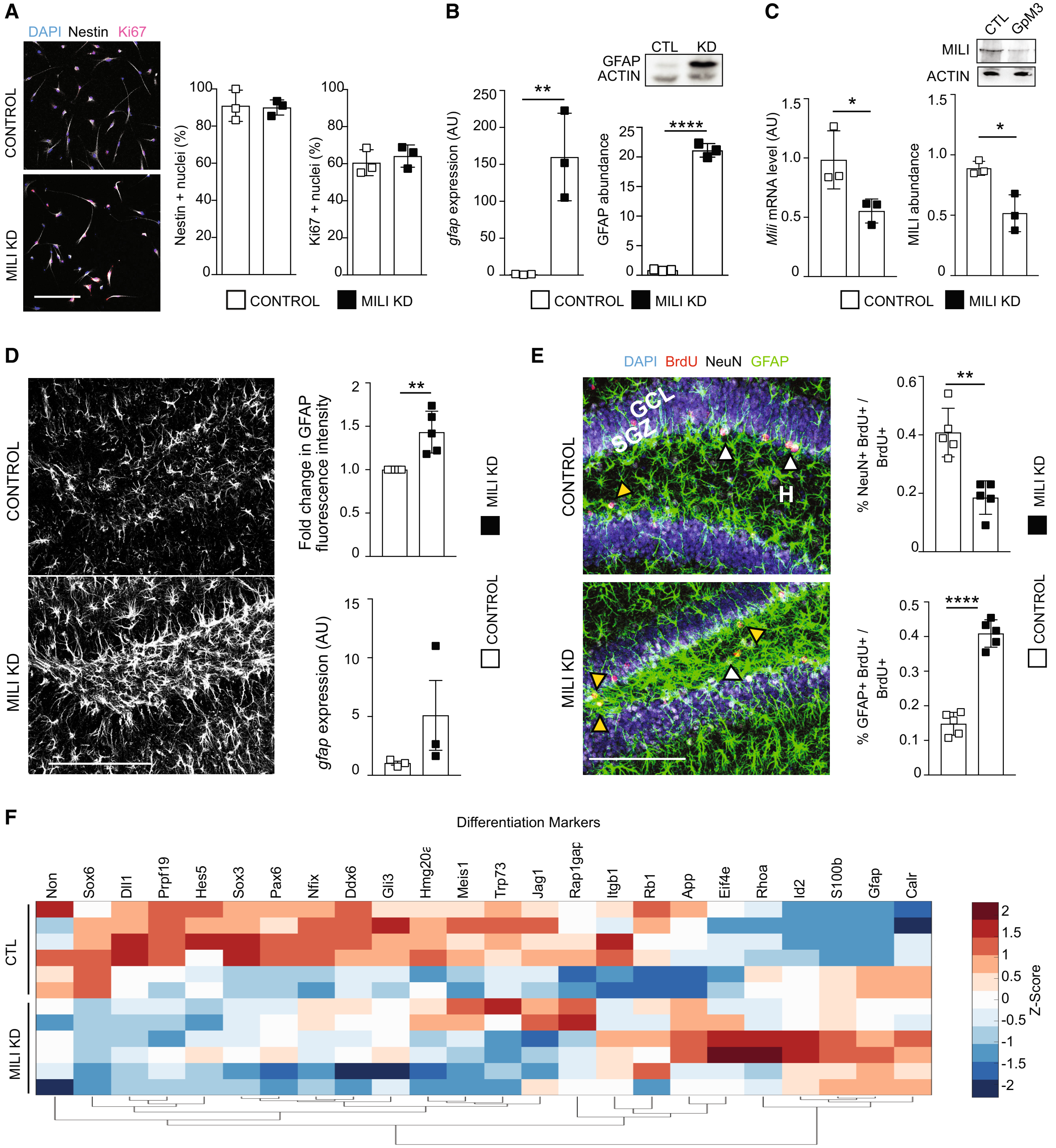
A
DAPI Nestin Ki67
CONTROL
MILI KD
Nestin + nuclei (%)
Ki67 + nuclei (%)
CONTROL MILI KD
B
CTL KD
GFAP
ACTIN
gfap expression (AU)
GFAP abundance
**
****
CONTROL MILI KD
C
CTL GpM3
MILI
ACTIN
Mili mRNA level (AU)
MILI abundance
*
CONTROL MILI KD
D
CONTROL
MILI KD
Fold change in GFAP fluorescence intensity
gfap expression (AU)
**
E
DAPI BrdU NeuN GFAP
GCL
SGZ
H
CONTROL
MILI KD
% NeuN+ BrdU+ / BrdU+
% GFAP+ BrdU+ / BrdU+
**
****
F
Differentiation Markers
Non
Sox6
Dll1
Prpf19
Hes5
Sox3
Pax6
Nfix
Ddx6
Gli3
Hmg20a
Meis1
Trp73
Jag1
Rap1gap
Itgb1
Rb1
App
Eif4e
Rhoa
Id2
S100b
Gfap
Calr
CTL
MILI KD
Z-Score
2
1.5
1
0.5
0
-0.5
-1
-1.5
-2

Figure EV3.

Figure EV3. Depletion of Mili and piRNAs does not alter stemness and proliferation and induces the expression of genes involved in astrogliogenesis. Related to Fig 4.

A (left) Confocal microscopy images of undifferentiated aNPCs transduced *in vitro* with viruses transcribing a Scrambled (Control) and shMILI (Mili KD), immunostained with anti-Nestin (white), or anti-Ki67 (purple) antibodies and stained for nuclear DNA with DAPI (blue); (right) Percentage of Nestin or $Ki67^{+}$ cells. over total cells.

B *Gfap* mRNA expression (left graph), western blot (inset), and quantification of Gfap protein abundance (right graph) in lysates from control and Mili KD neuroblasts at DIF7.

C *Mili* mRNA expression (left graph), western blot (inset), and quantification of Mili protein abundance (right graph) in lysates from mouse hippocampi 48 h after the injection of scrambled (Control) or GapmeR3 against *Mili* (Mili KD).

D Representative immunofluorescence micrograph of postnatal hippocampal sections immunostained for GFAP; (top right) quantification of GFAP fluorescence intensity level (top) in hippocampal section 30 dpi of GapmeR3 (Mili KD) compared with scrambled (Control). Expression of *Gfap* mRNA (bottom right) in the DG from mouse hippocampi 48 h after the injection of scrambled (Control) or GapmeR3 (Mili KD).

E (left) Representative immunofluorescence micrograph of postnatal hippocampal sections immunostained for GFAP (green), BrdU (red), NeuN (white), and nuclear DNA (blue) at 30 dpi of scrambled (Control) or GapmeR3 against Mili (Mili KD); (right) percentages of $NeuN^{+}BrdU^{+}$ (white arrowheads), or $GFAP^{+}BrdU^{+}$ (yellow arrowheads) double-positive cells over total $BrdU^{+}$ cells.

F RNA seq. expression data of genes encoding for proteins involved in astrogliogenesis and regulation of neuronal fate in Mili KD neuroblasts at DIF7, compared with Scrambled control. Expression heatmap correlation plots were computed by the k-means clustering method. Scale bar indicates *Z*-scores.

Data information: data are expressed as mean ± SEM, $n = 3$ (A–C, F) and $n = 5$ (D, E) biological replicates (in F each biological replicate was sequenced with two separate flow cells). *$P < 0.05$, **$P < 0.01$, ****$P < 0.0001$, as assessed by the two-tailed Student's *t*-test. GCL, granular cell layer, SGZ, subgranular zone. The scale bars represent 50 μm (A) and 100 μm (D, E).
Source data are available online for this figure.

 © 2022 The Authors

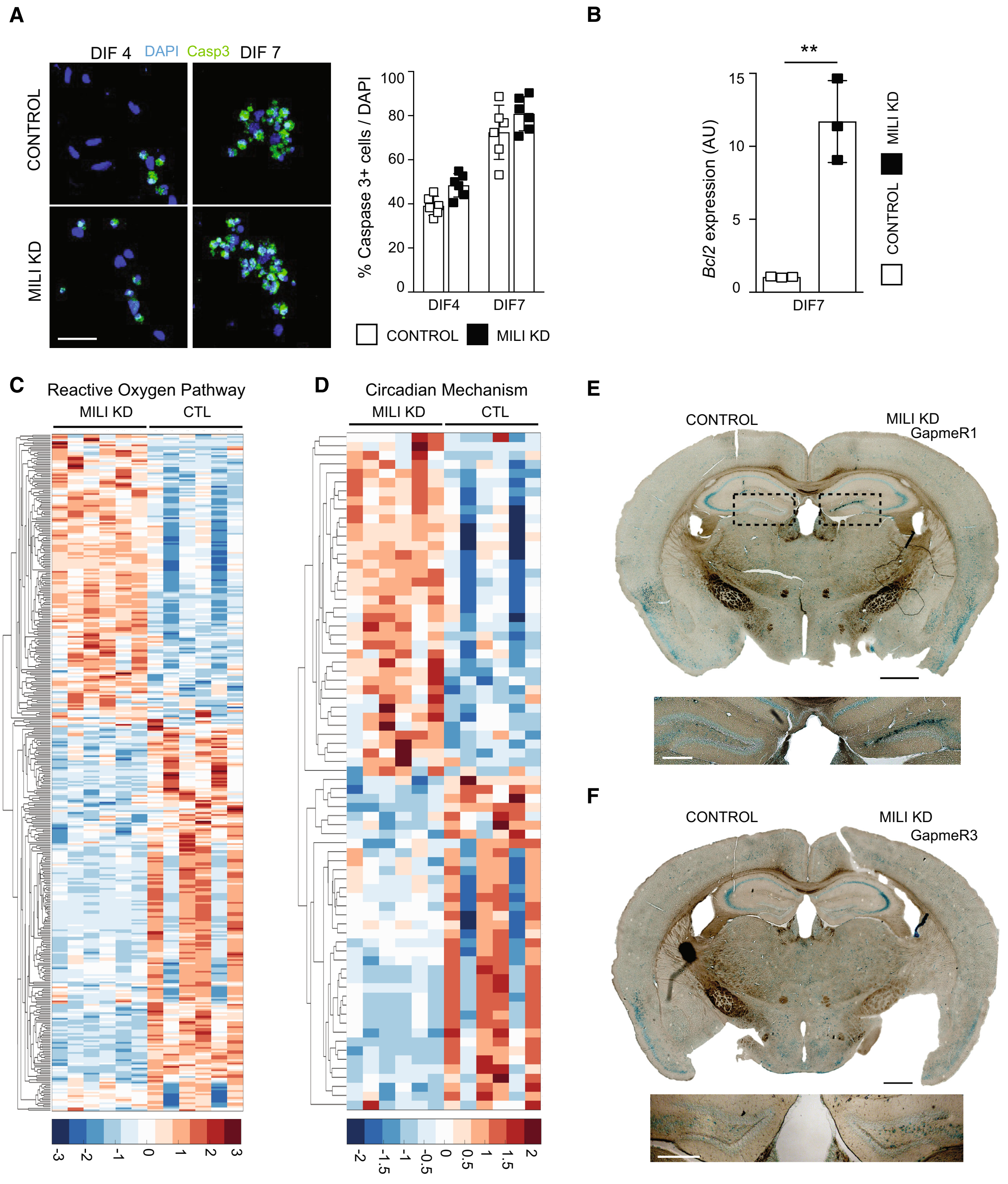
A
DIF 4
DAPI
Casp3
DIF 7
CONTROL
MILI KD
% Caspase 3+ cells / DAPI
100
80
60
40
20
0
DIF4
DIF7
CONTROL
MILI KD
B
**
Bcl2 expression (AU)
15
10
5
0
DIF7
CONTROL
MILI KD
C
Reactive Oxygen Pathway
MILI KD
CTL
-3
-2
-1
0
1
2
3
D
Circadian Mechanism
MILI KD
CTL
-2
-1.5
-1
-0.5
0
0.5
1
1.5
2
E
CONTROL
MILI KD
GapmeR1
F
CONTROL
MILI KD
GapmeR3

Figure EV4.

Figure EV4. Depletion of Mili and piRNAs does not lead to apoptosis and alters the expression of genes involved in inflammatory responses. Related to Fig 5.

A Representative fluorescence micrographs of control or Mili KD neuroblasts 4 or 7 days after spontaneous differentiation (DIF4, 7), immunostained with anti-cleaved caspase-3 (green) and for nuclear DNA with DAPI (blue). (Right) Percentage of cleaved Caspase-3^+ cells over total cells.

B *Bcl2* mRNA expression level in control or Mili KD neuroblasts at DIF7.

C, D Heatmap of differentially expressed transcripts in RNA seq from Mili KD neuroblasts, encoding proteins involved in ROS production (C) or circadian regulation (D). Target genes are listed in Dataset EV4. Expression heatmap correlation plots were computed by the k-means clustering method. Scale bar indicates *Z*-scores.

E, F Representative light-microscopy images of the ß-galactosidase staining in postnatal hippocampal sections, 30 dpi of scrambled (Control, left hemisphere) and GapmeR1 (E) or GapmeR3 (F) against *Mili* (Mili KD, right hemispheres). Dashed box in E indicates the areas shown in Fig 5. Bottom panels in (E, F) are higher magnification of the hippocampi shown in top panels.

Data information: data are expressed as mean ± SEM, $n = 6$ (A) and $n = 3$ (B–D) biological replicates (in C and D each biological replicate was sequenced with two separate flow cells). $^{**}P < 0.01$, as assessed by the two-tailed Student's *t*-test. The scale bars represent 50 µm (A), 1 mm (E, F top) and 500 µm (E, F bottom).

© 2022 The Authors

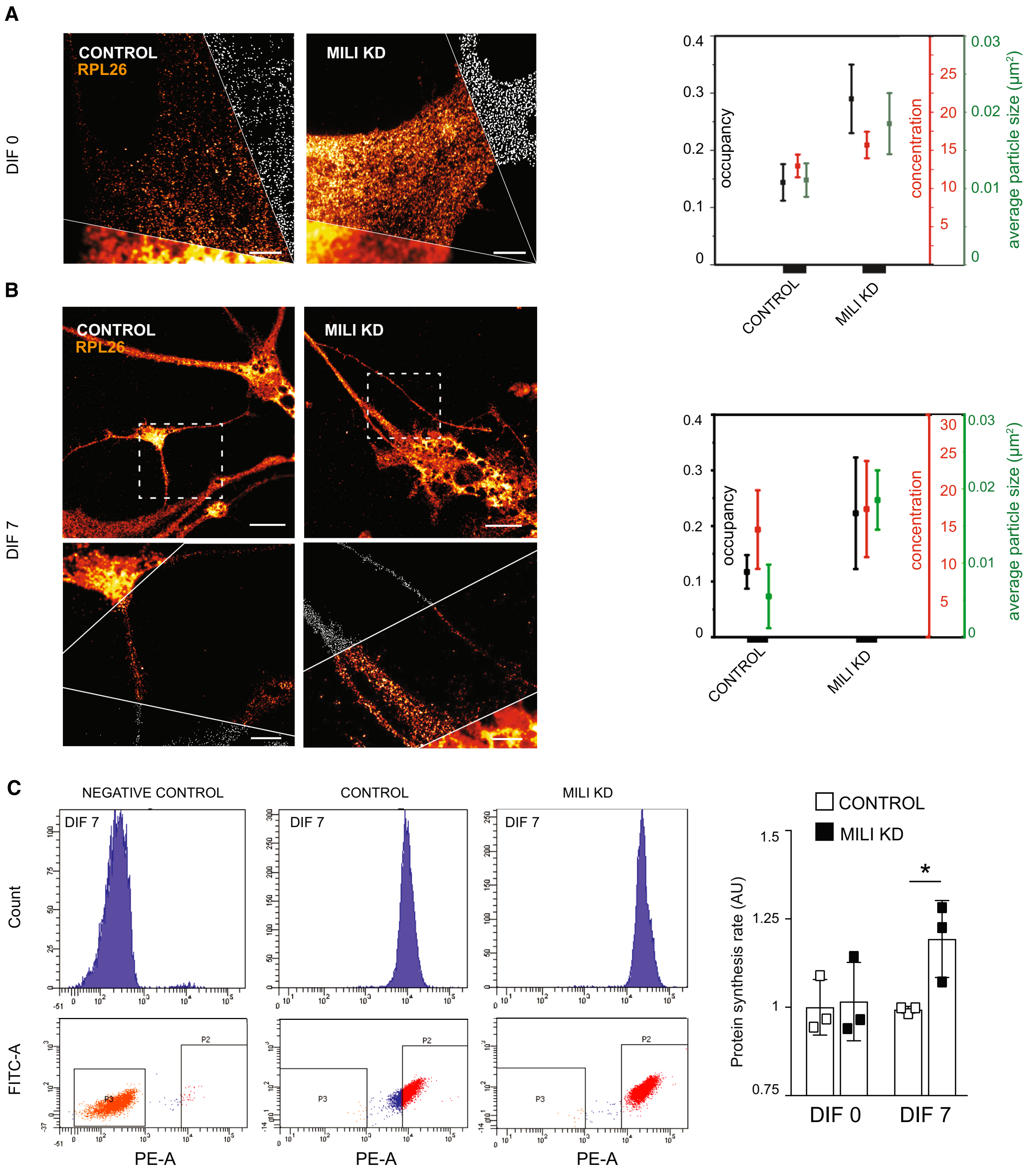
A
DIF 0
CONTROL
RPL26
MILI KD
occupancy
concentration
average particle size (μm²)
CONTROL
MILI KD
B
DIF 7
CONTROL
RPL26
MILI KD
occupancy
concentration
average particle size (μm²)
CONTROL
MILI KD
C
NEGATIVE CONTROL
CONTROL
MILI KD
DIF 7
Count
FITC-A
PE-A
P2
P3
CONTROL
MILI KD
Protein synthesis rate (AU)
DIF 0
DIF 7

Figure EV5.

◀ **Figure EV5. Depletion of Mili and piRNAs enhances polysome assembly and results in higher protein synthesis upon differentiation.**

A, B Representative micrographs (Middle cut: g-STED nanoscopy; Bottom: Confocal; Top: analysis) of control and Mili KD undifferentiated aNPCs (DIF0) and neuroblasts (DIF7) immunostained for the ribosomal protein RPL26. (Right) Normalized distributions of the occupancy, concentration and average particle size of each polyribosome particle in the indicated cells.

C Protein synthesis rate (right) as determined by OPP incorporation assay with flow cytometry (left) in control and Mili KD undifferentiated aNPCs (DIF0) and neuroblasts (DIF7).

Data information: data are expressed as mean ± SEM, $n = 3$ biological replicates. *$P < 0.05$, as assessed by the two-tailed Student's t-test. The scale bars represent 2 μm (A) and 10 μm (B).

 © 2022 The Authors
