# Supplementary material for: Piwil2 (Mili) sustains neurogenesis and prevents cellular senescence in the postnatal hippocampus
Source: EMBO Rep. 2022 Dec 6;24(2):e53801. doi: 10.15252/embr.202153801 (PMC9900342; doi:10.15252/embr.202153801)
Supplement: Supplementary file 12 [file EMBR-24-e53801-s003.pdf]

EMBO reports

Piwil2 (Mili) sustains neurogenesis and prevents cellular senescence in the postnatal hippocampus

Caterina Gasperini, Kiril Tuntevski, Silvia Beatini, Roberta Pelizzoli, Amanda Lo Van, Damiano Mangoni, Rosa Cossu, Giovanni Pascarella, Paolo Bianchini, Pascal Bielefeld, Margherita Scarpato, Meritxell Pons Espinal, Remo Sanges, Alberto Diaspro, Carlos Fitzsimons, Piero Carninci, Stefano Gustincich, and Davide De Pietri Tonelli
DOI: 10.15252/embr.202153801

Corresponding author(s): Davide De Pietri Tonelli (davide.depietri@iit.it)

Review Timeline:

Submission Date:	12th Aug 21
Editorial Decision:	14th Oct 21
Revision Received:	7th Oct 22
Editorial Decision:	19th Oct 22
Revision Received:	25th Oct 22
Accepted:	10th Nov 22

Editor: Martina Rembold

Transaction Report: This manuscript was transferred to EMBO reports following peer review at The EMBO Journal.

(Note: With the exception of the correction of typographical or spelling errors that could be a source of ambiguity, letters and reports are not edited. Depending on transfer agreements, referee reports obtained elsewhere may or may not be included in this compilation. Referee reports are anonymous unless the Referee chooses to sign their reports.)

1st Editorial Decision — 14th Oct 2021

Dear Davide,

Thank you for the submission of your revised manuscript to EMBO Reports. I have already forwarded you the referee reports we have received (included again below) and you have provided feedback on these.

The referees acknowledge that the revised manuscript has been improved but referee 2 and 3 are both concerned that the pulldown of Mili was not able to precipitate piRNAs from neural progenitors. As you know, I have discussed this issue further with the referees and with the team. We note that your data support a role for Mili in neuronal differentiation but whether this phenotype depends on piRNAs remains uncertain since the data on piRNAs are correlative in nature. As the study stands, we have thus the situation that an interesting, but ultimately knockdown-based phenotype cannot convincingly be linked to a mechanism. Please note that we have specifically asked you to address the interaction between piRNAs and MILI (referee 3, point 1), when we invited revision for EMBO reports, while we have not insisted on the analysis of MILI KO mice. The use of knockdown approaches instead of a knockout mouse can be discussed in the manuscript.
Yet, given the controversial nature of the field, we feel that further evidence must be provided whether Mili acts here via piRNAs, before the study can be considered for publication in EMBO Reports.

You have provided feedback on the referee concerns and indicated that you aim to optimize the IP conditions on one hand and try alternative binding assays on the other hand and we have decided to give you the chance to further revise your study and to address this point, i.e., whether MILI interacts with piRNAs in aNPCs.

We realize that these experiments are time consuming and that the outcome is uncertain. While further evidence whether or not MILI acts via piRNAs in aNPCs will be essential for publication in EMBO reports, I would like to inform you at this point that I have discussed your study with Eric Sawey, Executive Editor of Life Science Alliance (http://www.life-science-alliance.org/) who is in principle interested in publishing your work with minor revisions. In case you are interested in this option, I encourage you to discuss the required revision directly with Eric by contacting him at e.sawey@life-science-alliance.org.

In case you decide to engage in the revision for EMBO Reports, as discussed, please consider the following editorial points when you submit your revised manuscript:

- Please supply all figures as individual production quality files as .eps, .tif, .jpg (one file per figure).
Please download our Figure Preparation Guidelines (figure preparation pdf) from our Author Guidelines pages https://www.embopress.org/page/journal/14693178/authorguide for more info on how to prepare your figures.

- Data availability section: Please add the accession numbers for the small RNA seq data and the RNA seq data and links that resolve to the datasets on the ENA and GEO database, respectively.

- Please change the header "Declaration of Interests" to "Conflict of interest"

- Please add all information on funding into the relevant fields in our online submission system.

- Please add callouts to Fig. 6F, and to the panels of the EV figures.

- Please rename Tables S1-S4 to Dataset EV# and upload these individually with the file type Data Set. The file name and legend should be added as a new sheet.

- Table S5 could be changed into a Reagent table, that can be typeset within the methods section. You can download a Word or Excel template for this table from our Guide to Authors under "Structured Methods".
https://www.embopress.org/page/journal/14693178/authorguide
If you prefer to keep it as Appendix table, then you need to upload it as pdf called Appendix, with a title page and a table of content.

- Please submit Figure EV1 and Appendix Fig S1 as source data and split the data into one file per figure.

- Fig. 4C and EV2B: Please double-check the composition of the Western blots. The blots shown for actin seem not to match the source data in both cases. Please also reduce the contrast modification for the Mili blots in the figure.

- Fig. EV3B: It appears that the incorrect bands have been boxed in the source data. I think you show the second lane for the CTL and the fourth lane for shMILI (inverted in the figure). If this is correct, please indicate the splice site in the figure with a stippled line.

- Source data for Fig. 1: Please correct the labeling of the source data. What is labeled with (A) in the source data actually

belongs to panel (B) etc.
I also note that the bands have been compressed in the x-axis in Fig. 1C.

- We noted that the images shown in Fig. 5G are shown again in Fig. EV4E, as stated in the legend of Fig. EV4E. Please note this duplication also in the legend of 5G and please specify whether the subpanels in EV4E are the same as in 5G (boxed regions).

- Please add vertical space between pairs of blots in all figures.

- Please improve image quality and reduce contrast settings.

- I attach to this email a related manuscript file with comments by our data editors. Please address all comments and upload a revised file with tracked changes with your final manuscript submission.

- Finally, EMBO reports papers are accompanied online by A) a short (1-2 sentences) summary of the findings and their significance, B) 2-3 bullet points highlighting key results and C) a synopsis image that is 550x200-600 pixels large (width x height) in .png format. You can either show a model or key data in the synopsis image. Please note that the size is rather small and that text needs to be readable at the final size. Please send us this information along with the revised manuscript.

I look forward to seeing a revised form of your manuscript when it is ready. Please use this link to submit your revision:
https://embor.msubmit.net/cgi-bin/main.plex

Kind regards,

Martina

Martina Rembold, PhD
Senior Editor
EMBO reports

****

Referee #1:

The authors have responded to most of my comments and the manuscript is improved.

Referee #2:

De Tonelli et al. now provide a revised version of their manuscript. Although not all of the concerns raised by the reviewers were addressed, in my opinion some of the key issues were resolved and the manuscript is significantly improved. Therefore, I can support publication of this manuscript. However, since neither the links to piRNAs nor to translational control have been firmly established, the manuscript might be better suited as a short report.

Specific comments:
- Functional effects reported with Mili KD have now been replicated using an independent inhibitory GapMer. Although the use of the Mili KO (as suggested by reviewer 1) would arguably be a cleaner approach, I understand the problems with establishing a new mouse line during the pandemic.
- The role of piRNAs downstream of Mili has been toned down, which reflects the lack of conclusive data regarding an involvement of piRNA dysregulation in stem cell differentiation.
- The mechanistic results on translational control downstream of Mili have also been toned down and put into context.
- Most of my additional comments have been satisfactorily addressed, either with new data or a better presentation of already existing data.
- Comment 2 made by referee 3 is an important one, and it is still not clear to me why pulldown of Mili was not able to precipitate piRNAs from neural progenitors (in comparison to testes). It might be worth optimizing the pulldown conditions further, e.g. by using different salt concentrations in the buffers.

Referee #3:

The paper has been improved. However, I still think that if this manuscript is to rise to the level required for publication in EMBO rep/EMBO J, the authors should examine aNSC in Mili KO mice. I do understand the contingency due to SARS-Cov2. However,

even so, publishing this work in journals like EMBO rep/EMBO J would require characterizations of aNSC in PIWI KO mice.

It is also disappointing that the authors did not address convincingly the question of whether piRNAs and MILI function in aNPC. The authors stated that they have enough materials to obtain 25 µg RNA. If so, the authors should perform IP experiments with α-MILI antibody to confirm piRNAs expressed in aNPC are loaded onto MILI.

Referee #1:
The authors have responded to most of my comments and the manuscript is improved.

Author's answer
We thank the referee for insightful comments and constructive feedbacks, which certainly helped us to improve the quality of this work.

Referee #2:
De Tonelli et al. now provide a revised version of their manuscript. Although not all of the concerns raised by the reviewers were addressed, in my opinion some of the key issues were resolved and the manuscript is significantly improved. Therefore, I can support publication of this manuscript. [….].

Author's answer
We thank the reviewer for insightful comments and constructive feedbacks, which certainly helped us to improve the quality of this work. Answers to specific comments follows.

Comment #1
- Functional effects reported with Mili KD have now been replicated using an independent inhibitory GapMer. Although the use of the Mili KO (as suggested by reviewer 1) would arguably be a cleaner approach, I understand the problems with establishing a new mouse line during the pandemic.
Author's answer
In order to address this issue, we added a sentence in the discussion in which we state that: "…While some of these conclusions require further longitudinal investigations in Mili knockout mice (ideally, bearing Lox-P sites to selectively restrict the deletion of Mili to distinct NSC subpopulations), this study underpins a possible involvement of the piRNA pathway in brain plasticity and aging."

Comment #2
- …it is still not clear to me why pulldown of Mili was not able to precipitate piRNAs from neural progenitors (in comparison to testes). It might be worth optimizing the pulldown conditions further, e.g. by using different salt concentrations in the buffers.
Author's answer
We agree with the referee this is an important point. After buffer optimization and extensive antibody testing (~10 different anti-Mili ab), we now show that endogenous Mili and piRNAs form complexes in aNPCs (see new panels G-I in Fig 2).
Briefly, by processing aNPC lysates for co-immunoprecipitation (IP), we find that Mili (Fig. 2 G; specificity of the new anti-Mili Ab used in the IP in the Appendix Fig S2), binds to RNAs which have a peak size distribution of 25 nt (Fig. 2 H), in agreement with the known size of Mili-bound piRNAs in the mouse testis (Ding et al, 2017, full reference enclosed in the manuscript). Moreover, we find that the most abundant piRNA-cluster consensus sequences (piCS) of endogenous piRNAs are enriched in the Mili-IP compared to the control IP (Fig. 2I). These results, indicate that Mili and piRNAs form complexes in aNPCs, and further corroborate the idea that Mili likely acts here via piRNAs.

Additional Comments
- The role of piRNAs downstream of Mili has been toned down, which reflects the lack of conclusive data regarding an involvement of piRNA dysregulation in stem cell differentiation.
- The mechanistic results on translational control downstream of Mili have also been toned down and put into context.
- Most of my additional comments have been satisfactorily addressed, either with new data or a better presentation of already existing data.
Author's answer
In order to further address these points, we added a sentence in the discussion in which we state that: "….given this level of complexity, the mechanisms mediating gene-regulatory functions of the Mili-piRNA complexes in our model certainly warrant further investigation"

Referee #3:
Comment #1
The paper has been improved. However, I still think that if this manuscript is to rise to the level required for publication in EMBO rep/EMBO J, the authors should examine aNSC in Mili KO mice. I do understand the contingency due to SARS-Cov2. However, even so, publishing this work in journals like EMBO rep/EMBO J would require characterizations of aNSC in PIWI KO mice.
Author's answer
We are delighted to see that this referee acknowledges that the paper has been improved. To address the point related to Mili KO mice, we added a sentence in the discussion in which we state that: "…While some of these conclusions require further longitudinal investigations in Mili knockout mice (ideally, bearing Lox-P sites to selectively restrict the deletion of Mili to distinct NSC subpopulations), this study underpins a possible involvement of the piRNA pathway in brain plasticity and aging."

Comment #2
It is also disappointing that the authors did not address convincingly the question of whether piRNAs and MILI function in aNPC. The authors stated that they have enough materials to obtain 25 µg RNA. If so, the authors should perform IP experiments with α-MILI antibody to confirm piRNAs expressed in aNPC are loaded onto MILI.
of the starting material to obtain enough RNA.

Author's answer

We agree with the referee this is an important point. After buffer optimization and extensive antibody testing (~10 different anti-Mili ab), we now show that endogenous Mili and piRNAs form complexes in aNPCs (see new panels G-I in Fig 2).

Briefly, by processing aNPC lysates for co-immunoprecipitation (IP), we find that Mili (Fig. 2 G; specificity of the new anti-Mili Ab used in the IP in the Appendix Fig S2), binds to RNAs which have a peak size distribution of 25 nt (Fig. 2 H), in agreement with the known size of Mili-bound piRNAs in the mouse testis (Ding et al, 2017, full reference enclosed in the manuscript). Moreover, we find that the most abundant piRNA-cluster consensus sequences (piCS) of endogenous piRNAs are enriched in the Mili-IP compared to the control IP (Fig. 2I). These results, indicate that Mili and piRNAs form complexes in aNPCs, and further corroborate the idea that Mili likely acts here via piRNAs.

1st Revision - Editorial Decision 19th Oct 2022

Dear Davide,

Thank you for the submission of your revised manuscript to EMBO reports. The new data have been evaluated by former referee 2 (report copied below), who now supports publication of your manuscript in EMBO reports.

Before I can accept the manuscript, I need you to address some editorial points below:

1) Datasets:
- Dataset EV1 - EV4: Please remove the Dataset legends from the manuscript file and add them to a separate Tab in the Dataset .xls file called 'Legend'. The legend must also state the name of the dataset, e.g. Dataset EV1.
- For Dataset EV1, please specify whether n=3 refers to technical or biological replicates.

2) Appendix figures and source data:

- You have legends for Appendix Fig. S1 and Appendix Fig. S2 in the manuscript, but these data are actually included and will be published as source data. These legends need to be removed from the manuscript file. Instead, please relabel the source data, i.e., label the blot currently shown in panel (A) as (Fig. 1B) since it is the source data for Figure 1B. Or you write 'Full Western blot for Figure 1B" or similar. We do not need a legend for source data but it should be clear by looking at the images what they display.

- The same applies to the source data for Figure 2, Figure EV1, EV2 and Figure 4. Please note that we need one file per figure. I.e., one file for Figure 1, one file for Figure 2 etc. Then the main figure source data files are ZIPed together and the EV figure source data files are zipped together and uploaded as file type 'Source data".

- The data shown in Appendix Figure S2 (source data) for Fig. 2G are actually not source data but independent data to validate the antibody used for the IP. Please include these data in the main manuscript, e.g., in an EV figure.

- Figure 4C: please reduce the contrast settings to more accurately reflect the data shown in the source data file. The actin bands appear not to match between figure and source data. In the source data the actin band for CTL appears stronger than the one for GpM1, in the figure panel the opposite pattern is seen.

- Figure EV2B, GFAP Western blot. If you compare your figure panel and the source data the bands do not match. Th KD band shown in the figure is the second lane in the shMILI blot in the source data but mirrored and the CTL band is the first lane of the second CTL blot in your source data, again mirrored. Thus, you took lanes 4 and 5 from this blot and then mirrored these bands in the figure panel. Please correct the source data labeling.

3) Please upload Appendix Table S1 as Table 1.

4) 'Data and code availability' section: Please add links that resolve to the datasets on the ENA and GEO database, respectively. Please change the header to 'Data availability'.

5) Please change the header "Conflict of interest" to "Disclosure and Competing Interests Statement". For more information about this statement see
https://www.embopress.org/page/journal/14693178/authorguide#conflictsofinterest

6) Reagent Data Tables: Please use the Word or Excel template for our "Reagents and Tools table". You can download it from our website. Go to https://www.embopress.org/page/journal/14693178/authorguide
and search for 'Structured methods'. Then upload the table as file type 'Reagent table' in our system.

7) I attach to this email a related manuscript file with comments by our data editors. Please address all comments and upload a revised file with tracked changes with your final manuscript submission.

8) Please send the synopsis image in either .png or .jpg format

We look forward to seeing a final version of your manuscript as soon as possible.

With kind regards,

Martina

Martina Rembold, PhD
Senior Editor
EMBO reports

****

Referee #2:

The authors now performed the requested Mili-IP experiments and obtained convincing results which support an interaction of Mili with endogenous piRNAs. I therefore can now recommend publication.

The authors have addressed all minor editorial requests.

Dr. Davide De Pietri Tonelli
Fondazione Istituto Italiano di tecnologia
Neuroscience and brain technologies
Via Morego 30
Genoa 16163
Italy

Dear Davide,

I am very pleased to accept your manuscript for publication in the next available issue of EMBO reports. Thank you for your contribution to our journal.

At the end of this email I include important information about how to proceed.
There are two sections:
(1) THINGS TO DO NOW and
(2) a section on a newly launched funding scheme that allows you to publish Open Access in EMBO reports at no cost for you. See also the recent press release at https://www.embo.org/press-releases/increasing-participation-in-the-embo-programmes-across-europe/)

Please ensure that you take the time to read the information and complete and return the necessary forms to allow us to publish your manuscript as quickly as possible. If you need any assistance with (2) please let us know.

As part of the EMBO publication's Transparent Editorial Process, EMBO reports publishes online a Review Process File to accompany accepted manuscripts. As you are aware, this File will be published in conjunction with your paper and will include the referee reports, your point-by-point response and all pertinent correspondence relating to the manuscript.

If you do NOT want this File to be published, please inform the editorial office within 2 days, if you have not done so already, otherwise the File will be published by default [contact: emboreports@embo.org]. If you do opt out, the Review Process File link will point to the following statement: "No Review Process File is available with this article, as the authors have chosen not to make the review process public in this case." Please note that the author checklist will still be published even if you opt out of the transparent process.

Thank you again for your contribution to EMBO reports and congratulations on a successful publication. Please consider us again in the future for your most exciting work.

Kind regards,

Martina

Martina Rembold, PhD
Senior Editor
EMBO reports

****

(1) THINGS TO DO NOW:

Once your article has been received by Wiley for production, the corresponding author will receive an email from Wiley's Author Services system which will ask them to log in and will present them with the appropriate license for completion.

You will receive proofs by e-mail approximately 2-3 weeks after all relevant files have been sent to our Production Office; you should return your corrections within 2 days of receiving the proofs.

Please inform us if there is likely to be any difficulty in reaching you at the above address at that time. Failure to meet our deadlines may result in a delay of publication, or publication without your corrections.

All further communications concerning your paper should quote reference number EMBOR-2021-53801V3 and be addressed to emboreports@wiley.com.

Should you be planning a Press Release on your article, please get in contact with emboreports@wiley.com as early as possible, in order to coordinate publication and release dates.

****

(2) Open Access publication at no cost for authors in Italy

EMBO has recently launched funding and support options for life scientists in eleven different countries, among which is Italy (see the recent press release on embo.org - https://www.embo.org/press-releases/increasing-participation-in-the-embo-programmes-across-europe/). EMBO supports Open Access publication at no cost for authors in Italy, provided that they are not covered by a transformative agreement. I think in your case you are covered by a transformative agreement but the funding for it has been exhausted. Therefore, you qualify for the option that EMBO takes over the cost.

Please follow the instructions below:

Author services: EMBJ/EMBR
To qualify for EMBO to pay the article processing charges (APCs) for your manuscript:

- Sign-in to your Wiley Author Services account as instructed - you will receive a separate email with this information.
- Select the open access option and indicate that you do not qualify for a Transformative Agreement and that the author/author's institute will pay.
- Now choose that you pay per invoice and provide the payment details (either your own or your institute's) requested but giving the EMBO email address <publishing@embo.org> rather than your or your institute's email address.

The full process for completion of payment details in author services for hybrid journals is detailed at:
https://authorservices.wiley.com/author-resources/Journal-Authors/open-access/how-to-order-hybrid-open-access.html

EMBO PRESS

YOU MUST COMPLETE ALL CELLS WITH A PINK BACKGROUND ➔

PLEASE NOTE THAT THIS CHECKLIST WILL BE PUBLISHED ALONGSIDE YOUR PAPER

Corresponding Author Name: Davide De Pietri Tonelli
Journal Submitted to: EMBO Reports
Manuscript Number: EMBOR-2021-53801V1

Reporting Checklist For Life Sciences Articles (Rev. June 2017)

This checklist is used to ensure good reporting standards and to improve the reproducibility of published results. These guidelines are consistent with the Principles and Guidelines for Reporting Preclinical Research issued by the NIH in 2014. Please follow the journal's authorship guidelines in preparing your manuscript.

A- Figures
1. Data
The data shown in figures should satisfy the following conditions:

- the data were obtained and processed according to the field's best practice and are presented to reflect the results of the experiments in an accurate and unbiased manner.
- figure panels include only data points, measurements or observations that can be compared to each other in a scientifically meaningful way.
- graphs include clearly labeled error bars for independent experiments and sample sizes. Unless justified, error bars should not be shown for technical replicates.
- if n< 5, the individual data points from each experiment should be plotted and any statistical test employed should be justified
- Source Data should be included to report the data underlying graphs. Please follow the guidelines set out in the author ship guidelines on Data Presentation.

2. Captions

Each figure caption should contain the following information, for each panel where they are relevant:

- a specification of the experimental system investigated (eg cell line, species name).
- the assay(s) and method(s) used to carry out the reported observations and measurements
- an explicit mention of the biological and chemical entity(ies) that are being measured.
- an explicit mention of the biological and chemical entity(ies) that are altered/varied/perturbed in a controlled manner.

- the exact sample size (n) for each experimental group/condition, given as a number, not a range;
- a description of the sample collection allowing the reader to understand whether the samples represent technical or biological replicates (including how many animals, litters, cultures, etc.).
- a statement of how many times the experiment shown was independently replicated in the laboratory.
- definitions of statistical methods and measures:
  - common tests, such as t-test (please specify whether paired vs. unpaired), simple χ^2 tests, Wilcoxon and Mann-Whitney tests, can be unambiguously identified by name only, but more complex techniques should be described in the methods section;
  - are tests one-sided or two-sided?
  - are there adjustments for multiple comparisons?
  - exact statistical test results, e.g., P values = x but not P values < x;
  - definition of 'center values' as median or average;
  - definition of error bars as s.d. or s.e.m.

Any descriptions too long for the figure legend should be included in the methods section and/or with the source data.

In the pink boxes below, please ensure that the answers to the following questions are reported in the manuscript itself. Every question should be answered. If the question is not relevant to your research, please write NA (non applicable). We encourage you to include a specific subsection in the methods section for statistics, reagents, animal models and human subjects.

USEFUL LINKS FOR COMPLETING THIS FORM

http://www.antibodypedia.com
http://1degreebio.org
http://www.equator-network.org/reporting-guidelines/improving-bioscience-research-repor
http://grants.nih.gov/grants/olaw/olaw.htm
http://www.mrc.ac.uk/Ourresearch/Ethicsresearchguidance/Useofanimals/index.htm
http://ClinicalTrials.gov
http://www.consort-statement.org
http://www.consort-statement.org/checklists/view/32-consort/66-title
http://www.equator-network.org/reporting-guidelines/reporting-recommendations-for-tumo
http://datadryad.org
http://figshare.com
http://www.ncbi.nlm.nih.gov/gap
http://www.ebi.ac.uk/ega
http://biomodels.net/
http://biomodels.net/miriam/
http://jjj.biochem.sun.ac.za
https://osp.od.nih.gov/biosafety-biosecurity-and-emerging-biotechnology/
http://www.selectagents.gov/

B- Statistics and general methods

	Please fill out these boxes ➔ (Do not worry if you cannot see all your text once you press return)
1.a. How was the sample size chosen to ensure adequate power to detect a pre-specified effect size?	For the sake of reproducibility and consistency, we collected at least 3 repeated trials performed in different preparations and from at least 3 different experiments.
1.b. For animal studies, include a statement about sample size estimate even if no statistical methods were used.	At least 3 repeated measures were performed from different litters and from at least 3 (for molecular analysis) and 5 (for histology) different animals .
2. Describe inclusion/exclusion criteria if samples or animals were excluded from the analysis. Were the criteria pre-established?	NA
3. Were any steps taken to minimize the effects of subjective bias when allocating animals/samples to treatment (e.g. randomization procedure)? If yes, please describe.	NA
For animal studies, include a statement about randomization even if no randomization was used.	No randomization procedure has been done.
4.a. Were any steps taken to minimize the effects of subjective bias during group allocation or/and when assessing results (e.g. blinding of the investigator)? If yes please describe.	NA
4.b. For animal studies, include a statement about blinding even if no blinding was done	Researchers remained blinded throughout histological assessments. Groups were un-blinded at the end of each experiment upon statistical analysis.
5. For every figure, are statistical tests justified as appropriate?	Yes. Means between two groups were compared with two-tailed, unpaired Student's t- test. Comparisons of means from multiple groups with each other or against one control group were analyzed with one-way ANOVA followed by Bonferroni post hoc test.
Do the data meet the assumptions of the tests (e.g., normal distribution)? Describe any methods used to assess it.	The distribution of data in each set of experiments was tested for normality using D'Agostino-Pearson omnibus test or Shapiro-Wilk test.
Is there an estimate of variation within each group of data?	No estimate has been done. Variation among population means was computed and expressed as standard error of the mean.

Is the variance similar between the groups that are being statistically compared?	Equal variance has not been considered for statistical tests.

C- Reagents

6. To show that antibodies were profiled for use in the system under study (assay and species), provide a citation, catalog number and/or clone number, supplementary information or reference to an antibody validation profile. e.g., Antibodypedia (see link list at top right), 1DegreeBio (see link list at top right).	MILI antibody (Mouse, Santa Cruz, sc-37734; Rabbit polyclonal, kind gift of Dr. Hannon: Aravin et al., 2007 DOI: 10.1126/science.1142612); MIWI antibody (Rabbit polyclonal, kind gift of Dr. Hannon); ACTIN antibody (Rabbit, Abcam, ab13970); GADPH antibody (Rabbit, Santa Cruz, sc-25778); GFAP antibody (Rabbit; Dako, Z-0334); BrdU antibody (rat, Abcam, ab6326); KI67 antibody (rabbit, Abcam, ab15580); GFAP antibody (rabbit, Dako, Z-0334); NeuN antibody (mouse, Millipore, MAB377); RPL26 antibody (rabbit, Abcam, ab59567); Nestin antibody (mouse, Millipore, MAB353); Cleaved Caspase-3 (rabbit, Cell Signaling Technology, 9664).
7. Identify the source of cell lines and report if they were recently authenticated (e.g., by STR profiling) and tested for mycoplasma contamination.	aNPCs were isolated from the dentate gyrus of adult mice and expanded as previously described (Pons-Espinal et al., 2017, doi: 10.1016/j.stemcr.2017.02.012).

* for all hyperlinks, please see the table at the top right of the document

D- Animal Models

8. Report species, strain, gender, age of animals and genetic modification status where applicable. Please detail housing and husbandry conditions and the source of animals.	C57BL/6 and Td-Tomato flox/wt knock-in reporter mice (Jackson Laboratory stock number 007908), were housed at Istituto Italiano di Tecnologia (IIT); Nestin-GFP mice for the Kainic Acid experiments were housed at the Swammerdam Institute for Life Sciences, University of Amsterdam. All animal procedures were approved by IIT animal use committee and the Italian Ministry of health, or by the Commission for Animal Welfare at University of Amsterdam (DEC protocol 4925, AVD1110020184925), respectively and conducted in accordance with the Guide for the Care and Use of Laboratory Animals of the European Community Council Directives. All mice were group-housed under a 12-h light-dark cycle in a temperature and humidity-controlled environment with ad libitum access to food and water.
9. For experiments involving live vertebrates, include a statement of compliance with ethical regulations and identify the committee(s) approving the experiments.	Italian Ministry of Health and IIT Animal Use Committee approved the experiments, in accordance with the Guide for the Care and Use of Laboratory Animals of the European Community Council Directives.
10. We recommend consulting the ARRIVE guidelines (see link list at top right) (PLoS Biol. 8(6), e1000412, 2010) to ensure that other relevant aspects of animal studies are adequately reported. See author guidelines, under 'Reporting Guidelines'. See also: NIH (see link list at top right) and MRC (see link list at top right) recommendations. Please confirm compliance.	Td-Tomato flox/wt knock-in reporter mice: Madisen et al, 2010, doi: 10.1038/nn.2467. Nestin-GFP mice: Mignone et al, 2004, doi: 10.1002/cne.10964.

E- Human Subjects

11. Identify the committee(s) approving the study protocol.	NA
12. Include a statement confirming that informed consent was obtained from all subjects and that the experiments conformed to the principles set out in the WMA Declaration of Helsinki and the Department of Health and Human Services Belmont Report.	NA
13. For publication of patient photos, include a statement confirming that consent to publish was obtained.	NA
14. Report any restrictions on the availability (and/or on the use) of human data or samples.	NA
15. Report the clinical trial registration number (at ClinicalTrials.gov or equivalent), where applicable.	NA
16. For phase II and III randomized controlled trials, please refer to the CONSORT flow diagram (see link list at top right) and submit the CONSORT checklist (see link list at top right) with your submission. See author guidelines, under 'Reporting Guidelines'. Please confirm you have submitted this list.	NA
17. For tumor marker prognostic studies, we recommend that you follow the REMARK reporting guidelines (see link list at top right). See author guidelines, under 'Reporting Guidelines'. Please confirm you have followed these guidelines.	NA

F- Data Accessibility

18: Provide a "Data Availability" section at the end of the Materials & Methods, listing the accession codes for data generated in this study and deposited in a public database (e.g. RNA-Seq data: Gene Expression Omnibus GSE39462, Proteomics data: PRIDE PXD000208 etc.) Please refer to our author guidelines for 'Data Deposition'.<br><br>Data deposition in a public repository is mandatory for:<br>a. Protein, DNA and RNA sequences<br>b. Macromolecular structures<br>c. Crystallographic data for small molecules<br>d. Functional genomics data<br>e. Proteomics and molecular interactions	The small RNA sequencing data from this publication have been submitted to the ENA database and assigned the identifier PRJEB40241.The deep sequencing data from this publication have been submitted to the NCBI GEO database and assigned the identifier GSE182848.
19. Deposition is strongly recommended for any datasets that are central and integral to the study; please consider the journal's data policy. If no structured public repository exists for a given data type, we encourage the provision of datasets in the manuscript as a Supplementary Document (see author guidelines under 'Expanded View' or in unstructured repositories such as Dryad (see link list at top right) or Figshare (see link list at top right).	NA
20. Access to human clinical and genomic datasets should be provided with as few restrictions as possible while respecting ethical obligations to the patients and relevant medical and legal issues. If practically possible and compatible with the individual consent agreement used in the study, such data should be deposited in one of the major public access-controlled repositories such as dbGAP (see link list at top right) or EGA (see link list at top right).	NA
21. Computational models that are central and integral to a study should be shared without restrictions and provided in a machine-readable form. The relevant accession numbers or links should be provided. When possible, standardized format (SBML, CellML) should be used instead of scripts (e.g. MATLAB). Authors are strongly encouraged to follow the MIRIAM guidelines (see link list at top right) and deposit their model in a public database such as Biomodels (see link list at top right) or JWS Online (see link list at top right). If computer source code is provided with the paper, it should be deposited in a public repository or included in supplementary information.	NA

G- Dual use research of concern

22. Could your study fall under dual use research restrictions? Please check biosecurity documents (see link list at top right) and list of select agents and toxins (APHIS/CDC) (see link list at top right). According to our biosecurity guidelines, provide a statement only if it could.	No.
